# An Enhanced Red-Billed Blue Magpie Optimizer Based on Superior Data Driven for Numerical Optimization Problems

**DOI:** 10.3390/biomimetics10110780

**Published:** 2025-11-16

**Authors:** Siyan Li, Lei Kou

**Affiliations:** 1 Media and Communication, University of Westminster, London NW1 5LS, UK; lisiyanwk@126.com; 2 School of Qilu Transportation, Shandong University, Jinan 250061, China

**Keywords:** Red-Billed Blue Magpie Optimizer, swarm intelligence, engineering optimization, CEC 2017 test suite, dominant group driven

## Abstract

The Red-Billed Blue Magpie Optimizer (RBMO) is a recently introduced swarm-based meta-heuristic that has shown strong potential in engineering optimization but remains under-explored. To address its inherent limitations, this paper proposes an Enhanced RBMO (ERBMO) that synergistically incorporates two key strategies: a dominant-group-based two-stage covariance-driven strategy that captures evolutionary trends to improve population quality while reinforcing global exploration, and a Powell mechanism (PM) that eliminates dimensional stagnation and markedly strengthens convergence. Extensive experiments on the CEC 2017 benchmark suite demonstrate that ERBMO outperforms ten basic and improved algorithms in global exploration, local convergence accuracy and robustness, attaining Friedman ranks of 1.931, 1.621, 1.345 and 1.276 at 10D, 30D, 50D and 100D, respectively. Furthermore, empirical studies on practical engineering design problems confirm the algorithm’s capability to consistently deliver high-quality solutions, highlighting its broad applicability to real-world constrained optimization tasks. In future work, we will deploy the algorithm for real-world tasks such as UAV path-planning and resource-scheduling problems.

## 1. Introduction

Solving an optimization problem amounts to identifying the set of decision variables that minimizes or maximizes a given objective while satisfying prescribed constraints [1]. The recent explosion of science and technology has spawned increasingly complex tasks whose objective functions are high-dimensional, non-differentiable, non-convex and computationally expensive [2]. These characteristics often render traditional techniques ineffective or impractical because classical methods rely on rigid mathematical structures that demand exact analytical expressions of both the objective and the feasible region; they locate the optimum through direct or indirect calculations confined to a limited area [3]. In contrast, meta-heuristics deliver feasible, near-optimal solutions within a reasonable time or under restricted computational budgets by explicitly trading solution accuracy for runtime efficiency [4]. This trade-off has made them a promising alternative and has led to successful applications in path planning [5], job-shop scheduling [6], image segmentation [7], feature selection [8] and many other domains.

Swarm intelligence (SI) algorithms form a major branch of meta-heuristics that abstract the collective behavior of natural animal societies. By extracting social interaction models observed in different species, researchers have derived a variety of SI optimizers. Among them, Particle Swarm Optimization (PSO) [9]—patterned after the foraging flights of bird flocks—introduces velocity- and position-based update rules and has become one of the most intensively studied methods. Ant Colony Optimization (ACO) [10], another cornerstone algorithm, reproduces the pheromone trail laying and following behavior of foraging ants. Continued SI research has produced further nature-inspired schemes such as the Grey Wolf Optimizer (GWO) [11], Whale Optimization Algorithm (WOA) [12] and Harris Hawks Optimization (HHO) [13], all of which have been extensively analyzed and applied to diverse optimization tasks. More recently, emerging population-based algorithms—including Tuna Swarm Optimization (TSO) [14], the Parrot Optimizer (PO) [15], the Snow Geese Algorithm (SGA) [16] and the Crayfish Optimization Algorithm (COA) [17]—have expanded the SI repertoire with novel biological metaphors and updated search strategies.

**Figure 1:**
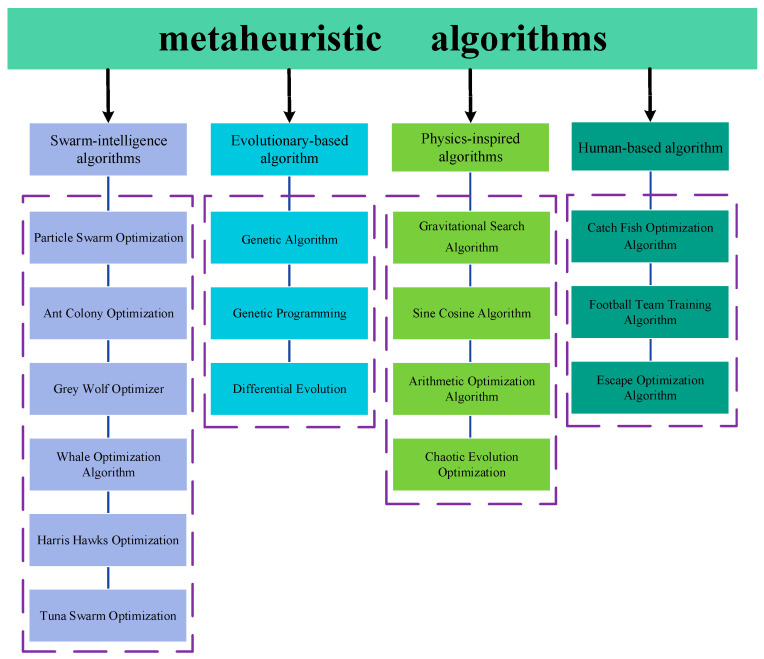
The classification of meta-heuristic algorithms.

Beyond the vigorous development of swarm intelligence algorithms, other categories of meta-heuristics continue to broaden their influence. Evolutionary-based methods such as the Genetic Algorithm (GA) [18], Genetic Programming (GP) [19] and Differential Evolution (DE) [20] are still intensively studied and applied across diverse domains. Physics-inspired algorithms—including Simulated Annealing (SA) [21], the Polar Lights Optimizer (PLO) [22] and the Gravitational Search Algorithm (GSA) [23]—model natural forces or physical phenomena, while mathematics-driven approaches like the Sine Cosine Algorithm (SCA) [24], Arithmetic Optimization Algorithm (AOA) [25] and Chaotic Evolution Optimization (CEO) [26] exploit trigonometric, arithmetic or chaotic operators. Additionally, human-based meta-heuristics such as the Catch Fish Optimization Algorithm (CFOA) [27], Football Team Training Algorithm (FTTA) [28] and Escape Optimization Algorithm (EOA) [29] are attracting increasing attention by emulating human learning, decision-making and social interaction behaviors. The classification of meta-heuristic algorithms is shown in Figure 1.

The Red-Billed Blue Magpie Optimizer (RBMO) is a novel swarm intelligence algorithm proposed by Fu et al. in 2024, inspired by the cooperative foraging behavior of the red-billed blue magpie [30]. Its effectiveness has been validated on benchmark suites, engineering-constrained problems and UAV path-planning tasks. Moreover, RBMO has been successfully applied to the optimal sizing of photovoltaic-plus-storage systems [31], neural network hyper-parameter tuning [32], target–trajectory prediction [33] and fault feature extraction [34]. Although RBMO has been applied to various optimization tasks, it still struggles with complex landscapes: its limited exploration–exploitation balance weakens population diversity and frequently causes premature convergence. Moreover, the No-Free-Lunch (NFL) theorem states that no single algorithm can excel across all problems [35]. Ye et al. strengthened exploration through a boundary-handling mechanism and balanced exploitation by embedding the search process in a guided framework [36]. Kong et al. boosted global reach and stability by generating the initial population with a logistic chaotic map [37]. Liu et al. let individual memory direct the search trajectory, achieving an adaptive trade-off between exploitation and exploration [32].

Consequently, this paper proposes an enhanced variant, ERBMO, which incorporates two complementary boosting techniques to overcome the deficiencies of the original RBMO. To thoroughly evaluate the proposed ERBMO algorithm, we conducted extensive experiments on the CEC 2017 test suite, the CEC 2022 test suite and a set of real-world engineering design problems. The results show that ERBMO consistently produces high-quality solutions across different scenarios. The core contributions of this study are summarized as follows:

(1) The ERBMO is proposed, integrating two enhancement techniques: a dominant-group-based two-stage covariance-driven strategy and a Powell mechanism.
(2) The dominant-group-based two-stage covariance-driven strategy captures effective information from dominant individuals to enhance the global search capability and adaptability to complex landscapes.
(3) The Powell mechanism further refines the search around the best solution, accelerating convergence and improving the final solution quality.
(4) Comprehensive evaluations, using the CEC 2017 test suite, and engineering design problems demonstrate that ERBMO outperforms other advanced algorithms in terms of convergence and robustness, with statistical validation confirming its consistent superiority across various optimization challenges.

The remainder of the paper is organized as follows. Section 2 reviews the biological inspiration and mathematical foundation of the basic RBMO. Section 3 details the proposed enhancement strategies and derives their mathematical models. Section 4 presents the comprehensive experimental results and discussions of the CEC 2017 test suite. Section 5 demonstrates the practical applicability of ERBMO through a variety of engineering design problems. Section 6 provides an in-depth performance analysis and discussion of all experiments. Finally, Section 7 summarizes the contributions and outlines future research directions.

## 2. Red-Billed Blue Magpie Optimizer

The Red-Billed Blue Magpie Optimizer (RBMO) is a swarm intelligence-based meta-heuristic proposed by Fu et al. [30], inspired by the foraging strategies of the red-billed blue magpie. By emulating the bird’s natural behaviors of searching, chasing, attacking prey and catching food, the algorithm constructs a set of corresponding search operators. A detailed description of the operators for each phase is presented below.

### 2.1. Initialization

As a population-based swarm intelligence algorithm, RBMO adopts the conventional initialization scheme. Within a *D*-dimensional search space bounded by *lb* and *ub*, the algorithm stochastically generates *N* candidate solutions—termed agents—according to Equation (1); these agents collectively constitute the initial RBMO population.

(1) $$X_{i}^{ini}=lb+rand\left(1,D\right)\times \left(ub-lb\right),i=1,2,\ldots ,N$$

where $X_{i}^{ini}$ denotes the initial position of the *i*-th red-billed blue magpie agent, and $rand\in {\left(0,1\right)}^{D}$ is a *D*-dimensional random vector drawn from a uniform distribution.

### 2.2. Search for Food

In the RBMO algorithm, each red-billed blue magpie agent employs two distinct predation strategies. Equation (2) models the search tactic used when targeting small prey, whereas Equation (3) describes the cooperative strategy adopted for hunting large prey.

(2) $$X_{i}^{new}=X_{i}^{old}+\left(\frac{1}{p}\times \sum_{m=1}^{p}X_{m}^{old}-X_{r1}^{old}\right)\times rand$$

(3) $$X_{i}^{new}=X_{i}^{old}+\left(\frac{1}{q}\times \sum_{m=1}^{q}X_{m}^{old}-X_{r2}^{old}\right)\times rand$$

where $X_{i}^{old}$ denotes the current position of the *i*-th agent, $X_{i}^{new}$ is its updated position and p is the number of agents that hunt in a small group (randomly selected between 2 and 5). $X_{m}^{old}$ represents the *m*-th agent randomly chosen from the current population when foraging in a small group, whereas q is the number of agents involved in large-group food searching (randomly selected between 2 and *N*). $X_{r1}^{old}$ and $X_{r2}^{old}$ are two additional agents randomly selected from the present population.

### 2.3. Attacking Prey

Once the prey is located, the red-billed blue magpie employs two distinct attack tactics—Equation (4) for small prey and Equation (5) for large prey—detailed below.

(4) $$X_{i}^{new}=X_{food}+CF\times \left(\frac{1}{p}\times \sum_{m=1}^{p}X_{m}^{old}-X_{i}^{old}\right)\times randn$$

(5) $$X_{i}^{new}=X_{food}+CF\times \left(\frac{1}{q}\times \sum_{m=1}^{q}X_{m}^{old}-X_{i}^{old}\right)\times randn$$

In the equations, $X_{food}$ denotes the position of the food source, representing the globally best agent. CF is dynamically updated according to Equation (6), while randn is a random scalar drawn from a standard normal distribution with a mean of 0 and variance of 1.

(6) $$CF={\left(1-\frac{FEs}{FEsMax}\right)}^{\left(\frac{2\times FEs}{FEsMax}\right)}$$

In the equation, FEs and FEsMax denote the current number of function evaluations and the maximum number of function evaluations, respectively.

### 2.4. Food Storage

After the search-and-attack phase, RBMO determines which candidate solutions survive into the next iteration by means of a deterministic survival rule: the newly generated position is accepted only if it yields a better (or equally good) objective value than its predecessor; otherwise, the previous position is retained.

(7) $$X_{i}^{next}=\left\{\begin{array}{c} X_{i}^{new},\;fitness_{i}^{new}<fitness_{i}^{old}\\ X_{i}^{old},\;fitness_{i}^{new}\geq fitness_{i}^{old} \end{array}\right.$$

In the equation, $X_{i}^{next}$ denotes the position of the *i*-th agent advanced to the next iteration, while $fitness_{i}^{old}$ and $fitness_{i}^{new}$ represent the fitness values of the *i*-th agent before and after the update, respectively. It should be noted that RBMO executes operators A and B sequentially in every iteration. For either operator, the probability of selecting the corresponding search strategy is set to 0.5.

## 3. The Proposed Enhanced Red-Billed Blue Magpie Optimizer

As a novel swarm intelligence method, RBMO has demonstrated competitive performance in its original publication and has been applied to several domains. Nevertheless, it still suffers from two limitations: (i) an insufficient global search capability, which leads to the premature loss of population diversity, and (ii) a weak local search ability, resulting in a slow convergence. To alleviate these deficiencies, two complementary mechanisms are introduced in this paper.

### 3.1. Dominant-Group-Based Two-Stage Covariance-Driven Strategy

Meta-heuristic algorithms rely on an intensive global search phase in the early stage to locate promising regions, followed by a refined local search phase to obtain solutions of higher precision. In RBMO, however, the “food-searching” (exploration) and “prey-attacking” (exploitation) operators are executed simultaneously in every single iteration. This permanent coupling causes the population to converge prematurely, so the algorithm is easily trapped in local optima during the latter search period—a drawback that becomes especially pronounced in high-dimensional landscapes with complex topologies. Moreover, although RBMO introduces information from different agents, it ignores the quality of these agents, so the guiding direction is not necessarily improved. To strengthen RBMO’s exploration ability and its capacity to escape from local optima, this work proposes a dominant-group-based two-stage covariance-driven strategy (DTC). The core of DTC lies in exploiting the guidance of a dominant subpopulation to steer the swarm toward promising regions and to enhance the exploration capacity. To fully utilize the information carried by these elite individuals, a covariance matrix is constructed on the fly. This matrix captures the shape and orientation of the current search distribution, enabling the algorithm to perform efficient, adaptive sampling of the solution space. The mathematical model of DTC is given below.

(8) $$Cov=\frac{1}{P}\sum_{i=1}^{P}\left(X_{i}^{P}-X_{w}\right)\times {\left(X_{i}^{P}-X_{w}\right)}^{T},\;X_{i}^{P}\in P_{d}$$

(9) $$X_{w}=\sum_{i=1}^{P}\omega_{i}\times X_{i}^{P},\;X_{i}^{P}\in P_{d}$$

(10) $$\omega_{i}=\ln\left(P+1\right)/\left(\sum_{i=1}^{P}\left(\ln\left(P+1\right)-\ln\left(i\right)\right)\right)$$

where Cov denotes the covariance matrix of the dominant subgroup, and $X_{w}$ represents its weighted centroid. Each agent in the dominant subgroup is assigned a weight coefficient $\omega_{i}$, which scales its contribution to the collective guidance. By incorporating these weights, the influence of individual agents is differentiated, enabling the swarm to orient itself more effectively toward promising regions of the search space. P is the number of the dominant group. $X_{i}^{P}$ is the agent in the dominant group. After obtaining Cov, the DTC devises two complementary search operators. Exploration phase—to guarantee a strong global coverage, Equation (11) amplifies the exploratory power by steering the search along the principal directions revealed by the dominant subgroup. Local-optimum escape—Equation (12) reinforces the ability to jump out of local optima by injecting information from both the best-so-far agent and a randomly chosen agent.

(11) $$X_{i}^{new}=X_{w}+g_{i},\;g_{i}\sim N\left(0,Cov\right)$$

(12) $$X_{i}^{new}=\frac{\left(X_{r1}^{old}+X_{w}+X_{food}\right)}{3}+g_{i},\;g_{i}\sim N\left(0,Cov\right)$$

**Figure 2:**
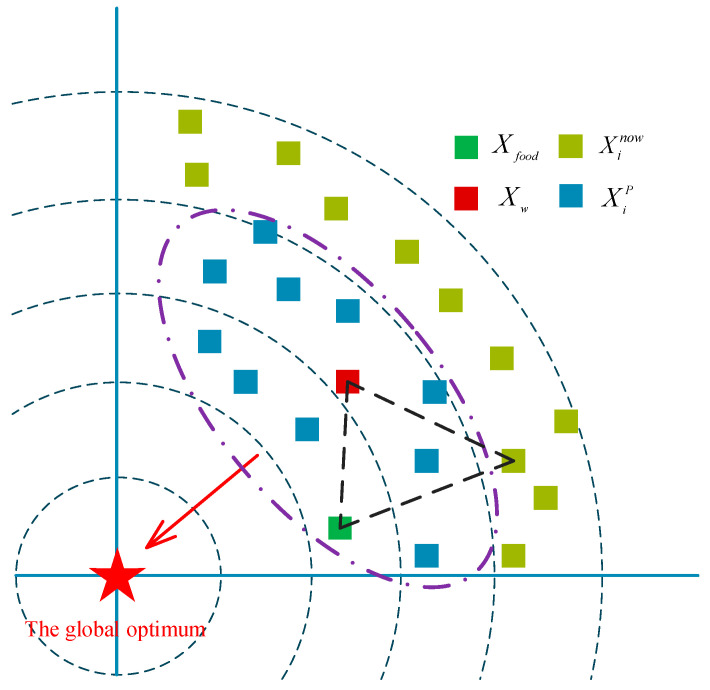
The schematic of DTC.

As illustrated in Figure 2, the dominant subgroup propels the swarm toward promising regions, the best agent accelerates convergence, and a randomly selected individual is employed to diversify search directions, thereby mitigating the risk of premature convergence to local optima.

### 3.2. Powell Mechanism

The RBMO algorithm lacks a mechanism that geometrically and adaptively refines solution trajectories, which limits its ability to perform fine-grained movements around promising regions and thus blunts the sharpness of convergence in the later exploitation phase. To overcome this weakness, this paper incorporates Powell’s Mechanism (PM). As a powerful derivative-free local search technique, PM accelerates convergence by constructing and reusing conjugate directions. Embedding this mechanism into ERBMO equips the algorithm with an enhanced capacity to locally exploit the best regions discovered during the global search.

The procedure consists of three consecutive stages: basic search, acceleration search and adjustment search. In each iteration, the basic search starts from the current position and performs successive one-dimensional minimizations along the existing directions to generate a new position vector. The acceleration search then computes the difference between two consecutive position vectors to obtain a direction that is closer to the optimum and replaces one of the original search directions with it. Finally, the adjustment search substitutes the current direction set with the newly acquired conjugate direction for the next iteration. This cycle repeats until a sufficiently accurate solution is obtained. The detailed implementation of the Powell mechanism is described as follows.

Step 1: Initialization: Choose a starting point $\gamma^{\left(0\right)}$ and D mutually independent search directions; specify a convergence tolerance Err>0 and initialize k=0.

Step 2: Basic search: Compute $\delta_{i}$ using Equation (13), then successively generate new base points $\gamma^{\left(1\right)}$, $\gamma^{\left(2\right)}$, …, $\gamma^{\left(D\right)}$ along the respective dimensions, as specified in Equation (14).

(13) $$F\left(\gamma^{\left(i\right)}+\delta_{i}\times d^{\left(i\right)}\right)=\min\left(F\left(\gamma^{\left(i\right)}+\delta_{i}\times d^{\left(i\right)}\right)\right)$$

(14) $$\gamma^{\left(i+1\right)}=\gamma^{\left(i\right)}+\delta_{i}\times d^{\left(i\right)},i=0,1,\ldots ,D-1$$

where $\delta_{i}$ represents the set of step lengths along each axis. If a component of $\delta_{i}$ is negative, a one-dimensional line search is conducted along the corresponding direction. When i<D−1, the index ii is incremented by 1 and Step 2 is revisited; otherwise, the algorithm proceeds to Step 3.

Step 3: Acceleration search: Compute the acceleration direction $d^{\left(D\right)}=\gamma^{\left(D\right)}-\gamma^{\left(0\right)}$. If the termination criterion on $\left\|d^{\left(D\right)}\right\|<Err$ is satisfied, exit; otherwise, proceed to Step 4.

Step 4: Compute the maximum-descent index tl using Equation (15). If Equation (16) is satisfied, the search directions for the next cycle remain unchanged; set $\gamma^{\left(0\right)}=\gamma^{\left(D\right)}$ and k=k+1 and proceed to Step 2. Otherwise, Step 5 is executed.

(15) $$F\left(\gamma^{\left(tl\right)}\right)-F\left(\gamma^{\left(tl+1\right)}\right)=\underset{0\leq i\leq D-1}{\max}\left\{F\left(\gamma^{\left(i\right)}\right)-F\left(\gamma^{\left(i+1\right)}\right)\right\}$$

(16) $$F\left(\gamma^{\left(0\right)}\right)-2\times F\left(\gamma^{\left(D\right)}\right)+F\left(2\times \gamma^{\left(D\right)}-\gamma^{\left(0\right)}\right)\geq 2\times \left(F\left(\gamma^{\left(tl\right)}\right)-F\left(\gamma^{\left(tl+1\right)}\right)\right)$$

Step 5: Adjusted search: Set $\gamma^{\left(tl+i\right)}=\gamma^{\left(tl+i+1\right)}$ to ensure the newly generated exploration directions remain linearly independent, then compute $\delta_{D}$ via Equation (14). Set $\gamma^{\left(0\right)}=\gamma^{\left(D+1\right)}=\gamma^{\left(D\right)}+\delta_{D}\times d^{\left(D\right)}$ and k=k+1 and proceed to Step 2. The PM method is used to further excavate promising regions, so PM is performed when FEs>0.9×FEsMax.

### 3.3. Implementation Steps of Proposed ERBMO Algorithm

**Figure 3:**
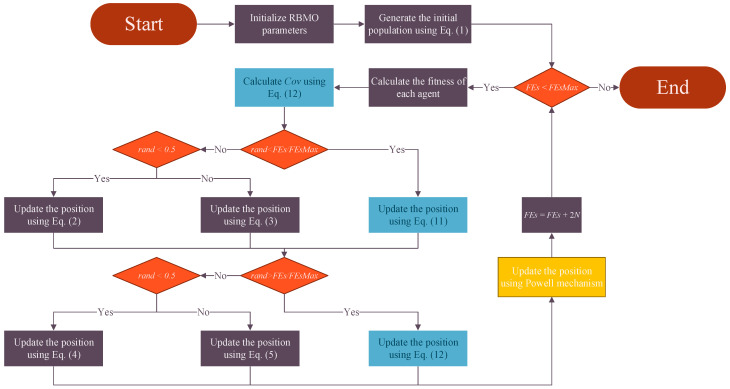
The flowchart of ERBMO.

Summarizing the above, the pseudo-code and flowchart of the ERBMO proposed in this paper are shown in Algorithm 1 and Figure 3.
**Algorithm 1:** Pseudo-code of ERBMO1: Initialize the RBMO parameters 2: Initialize the population *X* using Equation (1) 3: **While** *FEs* < *FEsMax* 4: Construct Cov using Equation (8)//**DTC** 5: **For**
*i* = 1: *N* **do** 6:  **//Exploration//** 7:  **If** *rand* < FEs/FEsMax
**Then**
8:       Update position of *i*th agent using Equation (11)//**DTC** 9:  **Eles** 10:    **If** rand < 0.5 **Then** 11:       Update position of *i*th agent using Equation (2)//Search for food 12:   **Eles** 13:      Update position of *i*th agent using Equation (3)//Search for food 14:   **End if** 15:   **End if** 16:  **//Exploitation//** 17:  **If** *rand* > FEs/FEsMax
**Then** 18:      Update position of *i*th agent using Equation (12)//**DTC** 19:  **Eles** 20:    **If** rand < 0.5 **Then** 21:      Update position of *i*th agent using Equation (4)//Attacking prey 22:   **Eles** 23:      Update position of *i*th agent using Equation (5)//Attacking prey 24:   **End if** 25:   **End if** 26: **End for** 27: *FEs* = *FEs* + 2*N* 28: **If** *FEs* > 0.9×FEsMax
**Then** 29: Update position of *X_food_* (the best agent) using Powell mechanism//**PM** 30: **End if** 31: **End while** 32: Return the best solution *X*_food_

Time complexity serves as a key indicator of algorithmic performance, reflecting both the intricacy and the computational expense of a method. For population-based optimizers, it is primarily governed by three factors: population size *N*, problem dimension *D* and the number of iterations *T*. In the baseline RBMO, the overall complexity is dictated by (i) population initialization and (ii) position updating. Consequently, the time complexity of RBMO can be expressed as follows.

$$O\left(RBMO\right)=O\left(initialization\right)+O\left(position\;updating\right)=O\left(N\times D\right)+O\left(2T\times N\times D\right)=O\left(\left(2T+1\right)\times N\times D\right)$$

For ERBMO, the population initialization routine remains identical to the original RBMO, so no extra cost is incurred. DTC is embedded into the existing search cycle rather than appended as an additional phase; consequently, the 2*N* position updates performed per iteration are shared among all operators, leaving the asymptotic cost of the update stage unchanged. The PM is invoked only on the current best agent; if it is executed *T*_1_ times during the entire run, its complexity is $O\left(T_{1}\times N\times D\right)$. Hence, the overall time complexity of ERBMO is given below.

$$O\left(ERBMO\right)=O\left(initialization\right)+O\left(position\;updating\right)=O\left(N\times D\right)+O\left(2T\times N\times D+T_{1}\times N\times D\right)=O\left(\left(2T+T_{1}+1\right)\times N\times D\right)$$

Although the theoretical time complexity of ERBMO is higher than that of the basic RBMO, the subsequent experimental results reveal that this modest increase in runtime is accompanied by a substantial gain in solution quality, making the trade-off fully acceptable. Moreover, all runs in this study are terminated once a prescribed maximum number of function evaluations is reached; this criterion eliminates any bias that could arise from the additional position updates performed within a single iteration, ensuring a fair comparison among algorithms.

## 4. Performance Analysis Using Benchmark Test Functions

Section 4 presents the experimental results of the proposed ERBMO on a suite of benchmark functions. Three sets of experiments were conducted to verify its effectiveness. First, a parameter sensitivity analysis was performed to determine the optimal parameter configuration of ERBMO. Second, ablation studies were carried out to quantify the contribution of each individual improvement strategy. Finally, ERBMO was compared with both basic and enhanced algorithms from different categories. The remainder of this section is organized as follows. Section 4.1 describes the benchmark functions. Section 4.2 details the experimental settings and the algorithms used for comparison. The parameter sensitivity analysis and the ablation study are presented in Section 4.3 and Section 4.4, respectively. The comprehensive comparison between ERBMO and the selected algorithms is provided in Section 4.5.

### 4.1. Review of the CEC 2017 Test Suite

This section assesses the performance of the proposed ERBMO algorithm using the CEC 2017 test suite (Dimensions = 10, 30, 50, 100). The CEC 2017 set comprises unimodal functions (F1, F3; F2 officially removed), multimodal functions (F4–F10), hybrid functions (F11–F20) and composite functions (F21–F30). Unimodal functions, which possess a single global optimum, are employed to quantify the exploitation capability and convergence speed. Multimodal, hybrid and composition functions, all characterized by multiple local optima, serve to examine the global exploration capacity and the ability to escape sub-optimal regions. Detailed specifications of the entire test suite are provided in Table 1.

### 4.2. Experimental Setup

The proposed ERBMO was benchmarked against ten state-of-the-art meta-heuristics representative of five algorithmic families: (a) evolutionary—AE [38] and LSHADE-SPACMA [39]; (b) physics-based—SAO [40] and ACGRIME [41]; (c) mathematics-based—QIO [42] and EPSCA [43]; (d) human-inspired—CFOA [27] and ISGTOA [44]; and (e) swarm intelligence—MPA [45] and EOSMA [46]. LSHADE-SPACMA serves as a state-of-the-art differential evolution variant, ACGRIME, EPSCA and ISGTOA represent distinct algorithmic classes whose superiority has been verified in their respective articles and EOSMA is a swarm-based enhancement that has demonstrated a competitive performance against IMODE, MadDE and LSHADE-cnEpSin. This selection covers both basic and enhanced variants, providing a comprehensive assessment of ERBMO’s relative superiority. The parameters of the comparison algorithm were determined by consulting the relevant literature, as shown in Table 2. Each function was run independently for 30 trials in order to guarantee a fair comparison. The maximum number of function evaluations (MaxFEs) was set to 1000× dimension. The best value (Min), standard deviation (Std) and mean fitness value (Mean) of the 30 trials were used in the statistical analysis. A core AMD R9 7945HX (2.5 GHz) CPU and 32 GB of RAM were used to conduct the research on MATLAB 2021b running on a Windows 11 operating system.

### 4.3. Parameter Sensitivity Analysis

In ERBMO, the size P of the elite pool governs the reliability of the estimated covariance matrix. An excessively large P incorporates low-fitness individuals and dilutes the search gradient, whereas an overly small P yields a statistically unreliable estimate. A parameter sensitivity study was therefore conducted to determine the most suitable value. Recognizing that the CEC 2017 suite spans multiple dimensions, we tested six linearly scaled settings: 5D, 10D, 15D, 20D, 25D and 30D.

**Figure 4:**
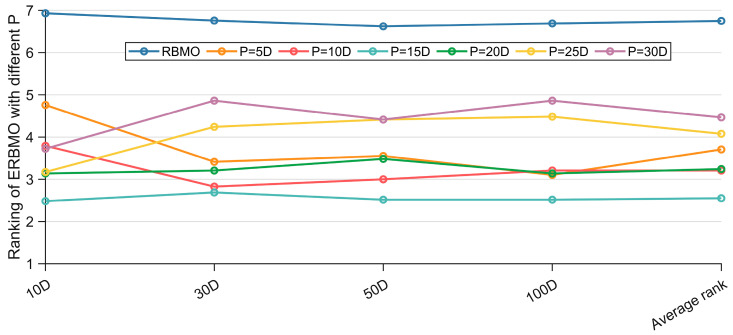
Ranking of ERBMO with different P.

Figure 4 reports the Friedman ranks obtained by basic RBMO and ERBMO with six dominant group sizes on the CEC 2017 suite; for brevity only, the statistical summaries are reported here and the full numerical results are provided in Table A1, Table A2, Table A3 and Table A4 of Appendix A. The Friedman test is conducted at a significance level of α = 0.05. Two observations are immediate. First, every ERBMO variant, regardless of P, consistently outperforms the original RBMO across all dimensions, confirming the usefulness of the proposed enhancements. Second, performance degrades when P is either too large (25D, 30D) or too small (5D). An excessive P introduces inferior solutions and corrupts the covariance estimate, whereas an insufficient P provides inadequate statistical support. ERBMO with P = 15D achieves the lowest average rank on every dimension and is therefore adopted in the remainder of this study.

### 4.4. Ablation Study

To quantify the individual contribution of each component, an ablation study was conducted on the CEC 2017 suite at four dimensionalities, comparing RBMO, the original algorithm DRBMO (RBMO + DTC only), PRBMO (RBMO + PM only) and ERBMO (RBMO + both); for brevity only, the statistical summaries are reported here and the full numerical results are provided in Table A5, Table A6, Table A7 and Table A8 of Appendix A.

**Figure 5:**
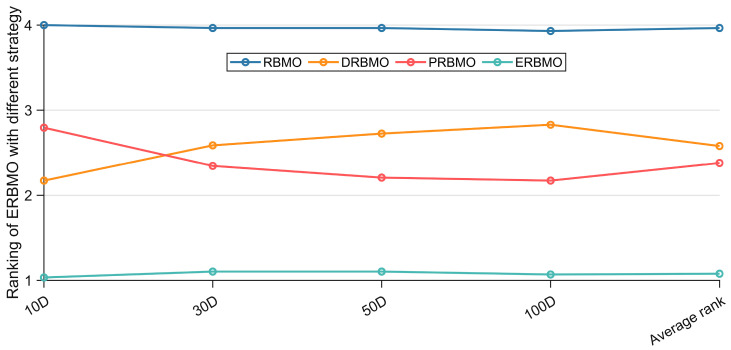
Ranking of ERBMO with different strategies.

Table 3 summarizes the Friedman ranks for RBMO, its ablated variants and ERBMO; all *p*-values in the last column are below 0.05, confirming significant differences among the configurations. Figure 5 depicts the corresponding rankings, from which three conclusions are drawn. First, ERBMO equipped with both enhancements consistently occupies the first rank, indicating that DTC and PM reinforce rather than hinder each other. Second, the single-strategy variants DRBMO and PRBMO both outperform the baseline RBMO, verifying the individual efficacy of each modification. Third, DRBMO surpasses PRBMO only at 10D and falls behind at higher dimensions, implying that the ability of the covariance matrix to capture variable interdependencies weakens as dimensionality increases, consequently degrading the benefit delivered by DTC.

The Nemenyi post hoc test evaluates the pairwise significance of the Friedman ranks. Algorithms whose average ranks differ by less than the critical difference value (CD) are linked by a horizontal bar, indicating no statistically significant discrepancy. The critical difference value (*CDV*) was calculated according to Equation (17), where M represents the number of algorithms and K represents the number of functions tested.

(17) $$CDV=q_{a}\times \sqrt{\frac{K\left(K+1\right)}{6M}}$$

**Figure 6:**
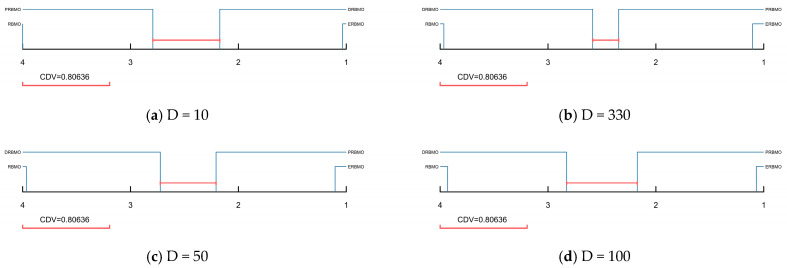
Nemenyi post hoc test of ERBMO with different strategies.

Figure 6 presents the CD diagrams for RBMO, DRBMO, PRBMO and ERBMO at 10D, 30D, 50D and 100D. As illustrated in Figure 5, ERBMO is isolated from all other algorithms, indicating statistically significant differences with both the ablated variants and the baseline RBMO. DRBMO and PRBMO are connected by the CD bar, suggesting that the individual contributions of the two enhancement strategies are statistically indistinguishable. RBMO remains unconnected to any competitor, confirming its significant inferiority. Consequently, the Friedman and Nemenyi tests jointly demonstrate that each proposed modification is effective and that their combination further amplifies the optimization capability of RBMO.

### 4.5. Comparative Evaluation with Other Algorithms

**Figure 7:**
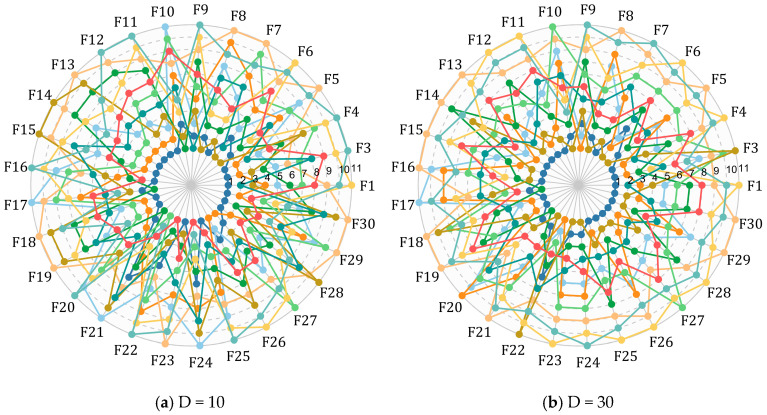

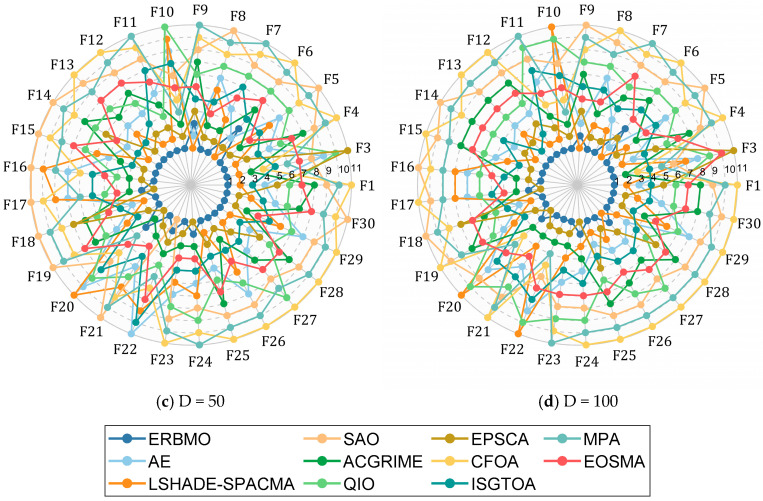
Ranking radar chart of ERBMO and competing algorithms.

This subsection benchmarks ERBMO against a diverse set of baseline and enhanced algorithms; the complete numerical results are provided in Table A9, Table A10, Table A11 and Table A12 of Appendix A, while only statistical summaries and visual comparisons are presented here for brevity. To visualize the relative performance of ERBMO and its competitors across the 29 CEC 2017 functions, a radar chart is constructed in Figure 7. Each algorithm is represented by a closed curve formed by its ranks on every function; smaller ranks yield a smaller enclosed area. The ERBMO curve consistently lies closest to the origin, indicating the best aggregate behavior. Subsequent subsections provide detailed convergence, robustness and statistical analyses (Friedman, Nemenyi and Wilcoxon rank-sum tests) to quantify this advantage.

**Figure 8:**
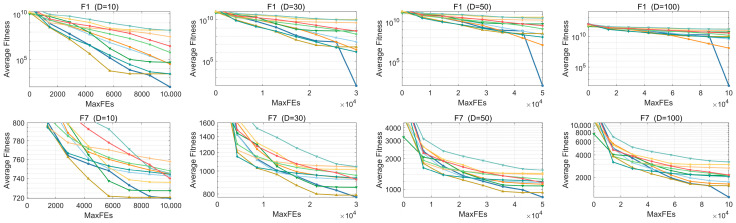

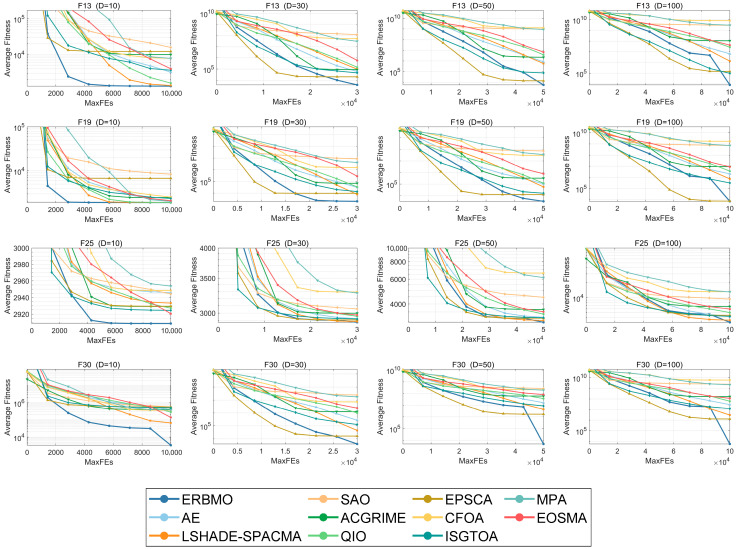
Convergence curves of ERBMO and competing algorithms.

Figure 8 illustrates the convergence behavior of ERBMO and its competitors on six representative functions: unimodal F1, multimodal F7, hybrid F13 and F19 and composition F25 and F30. For F1, ERBMO attains the highest accuracy, although its initial slope is moderate; the late-phase acceleration is produced by the PM-guided local refinement and the DTC-captured descent direction. For the multimodal F7, ERBMO, ACGRIME and EPSCA share a similar early convergence rate, yet only ERBMO continues to decrease and finally delivers the best value, indicating that DTC enlarges the search scope and avoids premature stagnation, while PM further exploits the most promising basins. For the hybrid F13 and F19, ERBMO converges fastest at 10D; at higher dimensions, the early stage is deliberately slowed because DTC enlarges the sample cloud, but a sharp drop reappears once PM concentrates the search on the reduced promising region. Finally, for the composition landscapes F25 and F30, ERBMO exhibits the steadiest and most persistent downward trajectory, confirming that the combined strategies enable the algorithm to track the composite ridges and descend continuously toward the global optimum. Collectively, DTC governs global exploration and escape, whereas PM performs fine-scale exploitation; their synergy equips ERBMO with a sustained and adaptable convergence capacity across varying function topologies.

**Figure 9:**
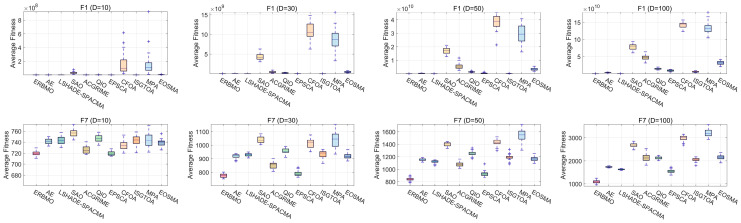

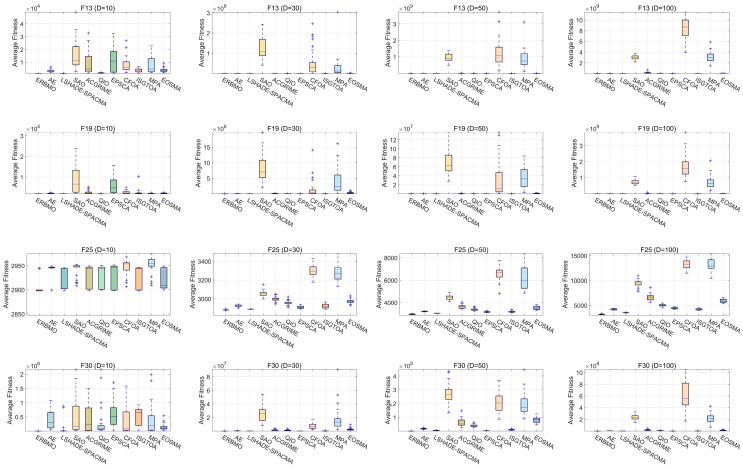
Boxplots of ERBMO and competing algorithms.

Robustness is essential for meta-heuristics because real-world deployment demands consistent performance. Figure 9 compares the solution distributions of ERBMO and its competitors through box-and-whisker diagrams that simultaneously expose the median accuracy, inter-quartile spread and outlier frequency. Lower and narrower boxes indicate both high precision and high stability; outliers and tall boxes immediately reveal volatility or failure runs. Across all six representative functions, ERBMO produces the most compact boxes with the fewest outliers and consistently occupies the lowest positions, demonstrating a superior and steady optimization performance under independent trials.

**Figure 10:**
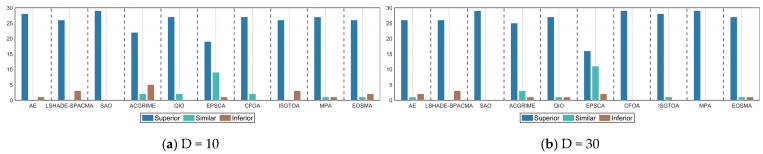

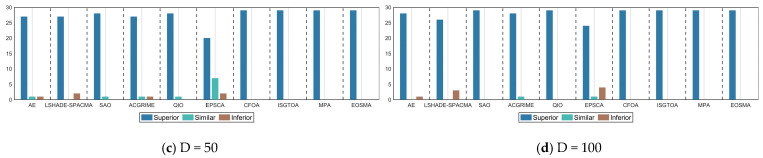
The number of “+/=/−” obtained by ERBMO and competing algorithms.

The statistical significance of the accuracy differences between ERBMO and each competitor was assessed with the Wilcoxon rank-sum test at α = 0.05. Table 4 summarizes the outcomes using the “+/=/−” notation, where “+” indicates that ERBMO is significantly superior, “=” denotes no significant difference and “−” signifies that ERBMO is significantly inferior. The aggregated results, visualized in Figure 10, show that ERBMO obtains more “+” than “−” against every rival across all four dimensionalities of the CEC 2017 suite, confirming its consistent advantage. Detailed pairwise comparisons are elaborated below.

For the 10D functions, the ERBMO algorithm records win/tie/loss counts of 28/0/1, 26/0/3, 29/0/0, 22/2/5, 27/2/0, 19/9/1, 27/2/0, 26/0/3, 27/1/1 and 26/1/2 against AE, LSHADE-SPACMA, SAO, ACGRIME, QIO EPSC, CFO, ISGTO, MPA and EOSMA, respectively. Thus, the ERBMO algorithm achieves a statistically significant superiority for at least 19 functions in every pairwise comparison.

For the 30D functions, the ERBMO algorithm records win/tie/loss counts of 26/1/, 26/0/3, 29/0/0, 25/3/1, 27/1/1, 16/11/2, 29/0/0, 28/1/0, 29/0/0 and 27/1/1 against AE, LSHADE-SPACMA, SAO, ACGRIME, QIO EPSC, CFO, ISGTO, MPA and EOSMA, respectively. Thus, the ERBMO algorithm achieves a statistically significant superiority for at least 16 functions in every pairwise comparison.

For the 50D functions, the ERBMO algorithm records win/tie/loss counts of 27/1/1, 27/0/2, 28/1/0, 27/1/1, 28/1/0, 20/7/2, 29/0/0, 29/0/0, 29/0/0 and 29/0/0 against AE, LSHADE-SPACMA, SAO, ACGRIME, QIO EPSC, CFO, ISGTO, MPA and EOSMA, respectively. Thus, the ERBMO algorithm achieves a statistically significant superiority for at least 20 functions in every pairwise comparison.

For the 100D functions, the ERBMO algorithm records win/tie/loss counts of 28/0/1, 26/0/3, 29/0/0, 28/1/0, 29/0/0, 24/1/4, 29/0/0, 29/0/0, 29/0/0 and 29/0/0 against AE, LSHADE-SPACMA, SAO, ACGRIME, QIO EPSC, CFO, ISGTO, MPA and EOSMA, respectively. Thus, the ERBMO algorithm achieves a statistically significant superiority for at least 24 functions in every pairwise comparison.

**Figure 11:**
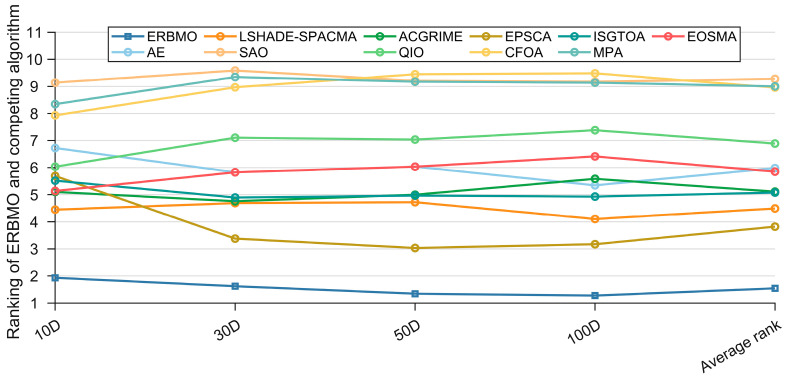
Ranking of ERBMO and competing algorithms.

Having established significant pairwise differences, we next evaluate the overall performance gap via the Friedman test. Figure 11 displays the average Friedman ranks of ERBMO and the competing algorithms, and the corresponding test statistics are summarized in Table 5. The *p*-values in the last column confirm significant performance differences among all contenders. ERBMO attains the best average rank of 1.543, followed by EPSCA (3.819) and L-SHADE-SPACMA (4.491), whereas the two basic algorithms MPA and SAO occupy the last two positions. Notably, ERBMO’s rank improves as dimensionality increases; corroborated by the ablation results, this indicates that the rival methods suffer a heavier performance degradation with high-dimensional functions, thereby elevating ERBMO’s relative standing. A detailed Friedman analysis is provided below.

For 10D functions, ERBMO, LSHADE-SPACMA and ACGRIME occupy the top three positions, followed in order by EOSMA, ISGTOA, EPSCA, QIO, AE, CFOA, MPA and SAO. Overall, ERBMO secures the leading position, with a Friedman score of 1.931, outperforming the second-ranked algorithm.

For 30D functions, ERBMO, EPSCA and LSHADE-SPACMA occupy the top three positions, followed in order by ACGRIME, ISGTOA, AE, EOSMA, QIO, CFOA, MPA and SAO. Overall, ERBMO secures the leading position, with a Friedman score of 1.621, outperforming the second-ranked algorithm.

For 50D functions, ERBMO, EPSCA and LSHADE-SPACMA occupy the top three positions, followed in order by ACGRIME, ISGTOA, AE, EOSMA, QIO, CFOA, MPA and SAO. Overall, ERBMO secures the leading position, with a Friedman score of 1.345, outperforming the second-ranked algorithm.

For 100D functions, ERBMO, EPSCA and LSHADE-SPACMA occupy the top three positions, followed in order by ISGTOA, AE, ACGRIME, EOSMA, QIO, MPA, SAO and CFOA. Overall, ERBMO secures the leading position, with a Friedman score of 1.276, outperforming the second-ranked algorithm.

**Figure 12:**
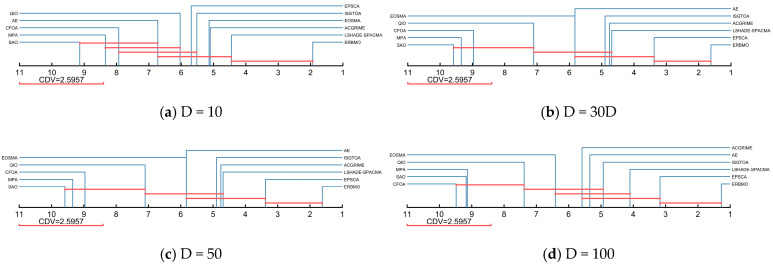
Nemenyi post hoc test of ERBMO and competing algorithms.

According to the Nemenyi post hoc test illustrated in Figure 12, ERBMO is linked to EPSCA at 30D, 50D and 100D, indicating no significant difference in these cases, and to LSHADE-SPACMA at 10D, implying a comparable performance only in low-dimensional instances. In contrast, ERBMO is separated from all remaining competitors by CDV, confirming its statistical superiority. Overall, ERBMO delivers consistently strong results against both basic and improved algorithms across the dimensional range.

## 5. Performance Analysis Using Engineering Optimization Problems

To appraise the practical utility and reliability of ERBMO, seven well-established constrained optimization problems, detailed in Table 6, are employed. Each case involves the simultaneous tuning of several continuous variables subject to stress, deflection or geometric restrictions, while the goal is to minimize the mass or production cost. Following common practice, each constrained problem is converted into an unconstrained equivalent by a static penalty framework: whenever any constraint is violated, a large positive term is added to the fitness value, so infeasible individuals are automatically driven out of the search population during evolution. To ensure impartiality, all engineering cases are tackled with identical algorithmic settings: the population size equals the value advised in the source literature, the optimization budget is capped at 1000 × D function evaluations and 30 independent runs are executed per problem to guarantee statistical reliability. Table 7 summarizes the results of ERBMO and its rivals, together with the outcomes of Friedman’s and Wilcoxon’s rank-sum tests.

Table 7 shows that ERBMO delivers the best overall performance, achieving the lowest average Friedman rank of 1.143. Specifically, it attains the best mean value for RW01–RW04 and RW06–RW07, and ranks second for RW05, slightly behind LSHADE-SPACMA. Moreover, the Wilcoxon rank-sum test indicates that ERBMO is significantly superior to every competitor in at least six engineering problems. These results firmly demonstrate the effectiveness and robustness of ERBMO in tackling constrained optimizations tasks.

## 6. Discussion

The ERBMO algorithm achieves a balanced intensification of exploitation and exploration through the synergy of DTC and PM, while preserving the ability to escape local optima. In both the CEC 2017 suite and real-world engineering problems, this synergy translates into measurable gains in optimization efficiency. PM injects fine-grained local search, whereas DTC accurately tracks the evolutionary gradient, giving ERBMO a clear edge on unimodal functions. By injecting diverse individuals, DTC enlarges the search scope and, guided by the detected trend, improves the solution quality; PM subsequently refines the most promising regions, allowing ERBMO to maintain a high search efficiency for complex landscapes. As dimensionality increases, however, DTC’s capacity to capture variable interdependencies weakens, so the DTC-only variant degrades. The sensitivity analysis confirms that an excessively large elite pool slows the search, whereas an overly small one fails to represent the evolutionary direction; the adopted 15D offers the best compromise. Statistically, Friedman, Nemenyi and Wilcoxon tests consistently corroborate the superiority of ERBMO, and the engineering case studies further attest to its practical applicability. Both DTC and PM are designed as self-contained, plug-and-play modules: the dominant-group covariance strategy can be transplanted into any population-based optimizer to enrich its exploration pattern, while the Powell mechanism—essentially a deterministic local searcher—can be seamlessly invoked whenever the host algorithm reveals weak exploitation, affording an immediate refinement boost without altering the original structure.

## 7. Conclusions

To compensate for the insufficient local exploitation and global exploration of the original RBMO, as well as to alleviate the loss of population diversity in the later stages of evolution, this paper proposes an Enhanced RBMO (ERBMO). The algorithm integrates a dominant-group-based two-stage covariance-driven strategy (DTC) for global search and a Powell mechanism (PM) for fine-scale exploitation. DTC dynamically injects diverse individuals to expand the search scope and capture evolutionary trends, while PM performs the in-depth refinement of promising regions; their synergy realizes an adaptive balance between exploration and exploitation. Comparative experiments on the CEC 2017 benchmark suite against ten representative meta-heuristic algorithms show that ERBMO achieves a superior convergence accuracy and stability across various landscapes. Engineering cases further verify its ability to consistently deliver high-quality feasible solutions, demonstrating a significant practical potential.

Nevertheless, ERBMO still presents the following limitations: Although the DTC parameter has been linearly scaled with dimension, its universality across a broader problem domain remains to be verified. In high-dimensional scenarios, DTC’s capacity to capture variable correlations decreases, reducing its guidance efficiency. As a local search operator, PM still has room for further enhancement. In summary, ERBMO is not a “final” algorithm, and future work will focus on the following: parameter self-adaptation, introducing reinforcement learning or deep learning to enable an online self-adaptation of the elite pool size *P* and penalty coefficients; expanded applications, developing binary, multi-objective and dynamic variants, and applying them to wider fields such as UAV path planning and supply chain optimization; and cross-domain technology fusion, exploring the use of large language models or knowledge graphs to automatically generate or refine search operators, thereby continuously improving global and local search efficiency. Moreover, in many domains, machine learning techniques are the primary tools for problem solving—examples include hybrid deep learning models for classification [47,48], Long Short-Term Memory networks for motion pattern recognition [49] and feed-forward neural networks for human health monitoring [50]. The performance of all these approaches is heavily influenced by their hyper-parameter settings. Consequently, we plan to employ the proposed optimizer to tune the hyper-parameters of such machine learning methods in our future work.

## Figures and Tables

**Table 1:** CEC 2017 test suite detailed introduction.

Type	No.	Functions Name	Min
Unimodal functions	F1	Shifted and Rotated Bent Cigar Function	100
	F3	Shifted and Rotated Zakharov Function	300
Multimodal functions	F4	Shifted and Rotated Rosenbrock’s Function	400
	F5	Shifted and Rotated Rastrigin’s Function	500
	F6	Shifted and Rotated Expanded Scaffer’s F6 Function	600
	F7	Shifted and Rotated Lunacek Bi_Rastrigin Function	700
	F8	Shifted and Rotated Non-Continuous Rastrigin’s Function	800
	F9	Shifted and Rotated Levy Function	900
	F10	Shifted and Rotated Schwefel’s Function	1000
Hybrid functions	F11	Hybrid Function 1 (N=3)	1100
	F12	Hybrid Function 2 (N = 3)	1200
	F13	Hybrid Function 3 (N = 3)	1300
	F14	Hybrid Function 4 (N = 4)	1400
	F15	Hybrid Function 5 (N = 4)	1500
	F16	Hybrid Function 6 (N = 4)	1600
	F17	Hybrid Function 6 (N = 5)	1700
	F18	Hybrid Function 6 (N = 5)	1800
	F19	Hybrid Function 6 (N = 5)	1900
	F20	Hybrid Function 6 (N = 6)	2000
Composition functions	F21	Composition Function 1 (N = 3)	2100
	F22	Composition Function 2 (N = 3)	2200
	F23	Composition Function 3 (N = 4)	2300
	F24	Composition Function 4 (N = 4)	2400
	F25	Composition Function 5 (N = 5)	2500
	F26	Composition Function 6 (N = 5)	2600
	F27	Composition Function 7 (N = 6)	2700
	F28	Composition Function 8 (N = 6)	2800
	F29	Composition Function 9 (N = 3)	2900
	F30	Composition Function 10 (N = 3)	3000

**Table 2:** Parameter setting of each algorithm.

Algorithm Setting	
ERBMO	N=30D,δ=0.2,a=10,S=3000,Ar=30D
RBMO	N=150,δ=0.2
AE	N=30,a=4
LSHADE-SPACMA	N=18D,H=6,F=0.5,CR=0.5
SAO	N=30,a=0.35,β=5/7
ACGRIME	N=30,a=4,w=5
QIO	N=30,a=0.7,b=0.15
EPSCA	N=30,a=2,c=90
CFOA	N=30
ISGTOA	N=50,λ=2
MPA	N=30,FADs=0.2,P=0.5
EOSMA	$N=30,a_{1}=2,a_{2}=1,GP=0.5,z=0.7$

**Table 3:** Friedman test results of ERBMO with different strategies.

Test Suite	Dimension	RBMO	DRBMO	PRBMO	ERBMO	*p*-Value
CEC 2017	10	4.000	2.172	2.793	1.034	3.25E-17
	30	3.966	2.586	2.345	1.103	1.71E-15
	50	3.966	2.724	2.207	1.103	6.96E-16
	100	3.931	2.828	2.172	1.069	3.62E-16
Average rank		3.966	2.578	2.379	1.078	
Overall rank		4	3	2	1	

**Table 4:** Wilcoxon rank-sum test results of ERBMO and competing algorithms.

ERBMO vs. (+/=/−)	Dimension	AE	LSHADE-SPACMA	SAO	ACGRIME	QIO	EPSCA	CFOA	ISGTOA	MPA	EOSMA
CEC 2017 test suite	10D	28/0/1	26/0/3	29/0/0	22/2/5	27/2/0	19/9/1	27/2/0	26/0/3	27/1/1	26/1/2
	30D	26/1/2	26/0/3	29/0/0	25/3/1	27/1/1	16/11/2	29/0/0	28/1/0	29/0/0	27/1/1
	50D	27/1/1	27/0/2	28/1/0	27/1/1	28/1/0	20/7/2	29/0/0	29/0/0	29/0/0	29/0/0
	100D	28/0/1	26/0/3	29/0/0	28/1/0	29/0/0	24/1/4	29/0/0	29/0/0	29/0/0	29/0/0

**Table 5:** Friedman test results of ERBMO and competing algorithms.

Test Suite	Dimension	ERBMO	AE	LSHADE-SPACMA	SAO	ACGRIME	QIO	EPSCA	CFOA	ISGTOA	MPA	EOSMA	*p*-Value
CEC 2017	10	1.931	6.724	4.448	9.138	5.103	6.034	5.690	7.931	5.517	8.345	5.138	2.57E−18
	30	1.621	5.828	4.690	9.586	4.759	7.103	3.379	8.966	4.897	9.345	5.828	2.27E−31
	50	1.345	6.034	4.724	9.207	5.000	7.034	3.034	9.448	4.966	9.172	6.034	6.43E−33
	100	1.276	5.345	4.103	9.172	5.586	7.379	3.172	9.483	4.931	9.138	6.414	3.57E−34
Average rank		1.543	5.983	4.491	9.276	5.112	6.888	3.819	8.957	5.078	9.000	5.853	
Overall rank		1	7	3	10	4	8	2	9	5	11	6	

**Table 6:** Details of engineering design optimization.

Problem	Name	D	g	h
RW01	Tension/compression spring design problem	3	3	0
RW02	Pressure vessel design problem	4	4	0
RW03	Three-bar truss design problem	2	3	0
RW04	Welded beam design problem	4	5	0
RW05	Gear train design problem	4	1	1
RW06	Cantilever beam design problem	5	1	0
RW07	Step-cone pulley problem	5	8	3

**Table 7:** Results of ERBMO and competing algorithms for engineering design optimization.

No.	Index	ERBMO	AE	LSHADE-SPACMA	SAO	ACGRIME	QIO	EPSCA	CFOA	ISGTOA	MPA	EOSMA
RW1	Best	1.2665E−02	1.2706E−02	1.2678E−02	1.2684E−02	1.2666E−02	1.2739E−02	1.2668E−02	1.2689E−02	1.2684E−02	1.2669E−02	1.2668E−02
	Mean	1.2789E−02	1.2894E−02	1.2802E−02	1.3560E−02	1.3570E−02	1.2992E−02	1.4256E−02	1.2832E−02	1.2889E−02	1.3210E−02	1.2843E−02
	Std	2.7743E−04	1.2984E−04	1.6821E−04	1.0424E−03	1.0615E−03	1.7389E−04	1.9198E−03	1.0249E−04	1.7816E−04	6.0253E−04	2.4980E−04
	Rank	1	6	2	9	10	7	11	3	5	8	4
RW2	Best	5.8699E+03	5.9464E+03	6.3145E+03	5.8702E+03	5.9176E+03	5.9953E+03	5.8701E+03	5.9155E+03	5.8822E+03	5.9294E+03	6.0259E+03
	Mean	5.9842E+03	6.1549E+03	6.6197E+03	6.3589E+03	6.5246E+03	6.3715E+03	6.3287E+03	8.6562E+03	6.2676E+03	8.1353E+03	6.4180E+03
	Std	1.9772E+02	1.3423E+02	3.6078E+02	4.4269E+02	4.2925E+02	2.3362E+02	4.9684E+02	4.8209E+03	3.8940E+02	1.8246E+03	2.8060E+02
	Rank	1	2	9	5	8	6	4	11	3	10	7
RW3	Best	2.6389E+02	2.6389E+02	2.6389E+02	2.6389E+02	2.6389E+02	2.6389E+02	2.6389E+02	2.6389E+02	2.6389E+02	2.6389E+02	2.6389E+02
	Mean	2.6389E+02	2.6389E+02	2.6389E+02	2.6393E+02	2.6428E+02	2.6389E+02	2.6407E+02	2.6392E+02	2.6389E+02	2.6404E+02	2.6390E+02
	Std	9.8456E−14	5.0411E−05	7.4121E−09	1.6411E−01	9.4923E−01	1.5163E−04	4.5541E−01	4.4870E−02	1.3866E−03	1.8984E−01	1.5036E−02
	Rank	1	3	2	8	11	4	10	7	5	9	6
RW4	Best	1.6928E+00	1.6976E+00	1.6957E+00	1.6941E+00	1.7013E+00	1.7053E+00	1.6929E+00	1.7049E+00	1.6974E+00	1.7144E+00	1.6970E+00
	Mean	1.6936E+00	1.7017E+00	1.7014E+00	1.7081E+00	1.9321E+00	1.7361E+00	1.7558E+00	1.9629E+00	1.7112E+00	1.9481E+00	1.7228E+00
	Std	2.1452E−03	3.1584E−03	5.4591E−03	3.0299E−02	2.5899E−01	2.4166E−02	1.0001E−01	1.9417E−01	8.7424E−03	1.7113E−01	3.0107E−02
	Rank	1	3	2	4	9	7	8	11	5	10	6
RW5	Best	2.7009E−12	2.7009E−12	2.7009E−12	2.3078E−11	2.7009E−12	2.7009E−12	2.3078E−11	2.3078E−11	2.7009E−12	2.3078E−11	2.7009E−12
	Mean	2.8642E−10	2.8945E−10	1.1750E−10	3.5673E−09	3.8317E−09	8.9422E−10	1.0833E−08	1.4412E−09	9.4150E−10	6.5431E−09	7.0914E−10
	Std	5.6256E−10	6.3717E−10	4.2655E−10	6.3578E−09	6.6441E−09	1.0004E−09	3.2254E−08	2.4419E−09	1.0243E−09	8.1094E−09	7.7716E−10
	Rank	2	3	1	8	9	5	11	7	6	10	4
RW6	Best	1.3400E+00	1.3401E+00	1.3401E+00	1.3401E+00	1.3401E+00	1.3401E+00	1.3400E+00	1.3472E+00	1.3405E+00	1.4232E+00	1.3402E+00
	Mean	1.3400E+00	1.3407E+00	1.3414E+00	1.3411E+00	1.3426E+00	1.3424E+00	1.3404E+00	2.0288E+00	1.3421E+00	1.7357E+00	1.3438E+00
	Std	2.1173E−04	3.7131E−04	9.0396E−04	8.1788E−04	3.2244E−03	1.6931E−03	5.5173E−04	4.8095E−01	1.3801E−03	2.3342E−01	3.8196E−03
	Rank	1	3	5	4	8	7	2	11	6	10	9
RW7	Best	1.6086E+01	1.6275E+01	1.6110E+01	1.6166E+01	1.6378E+01	1.6422E+01	1.6086E+01	1.6290E+01	1.6120E+01	1.6244E+01	1.6471E+01
	Mean	1.6136E+01	1.6537E+01	1.6205E+01	1.6869E+01	1.6819E+01	1.6719E+01	1.6473E+01	2.1216E+01	1.6751E+01	1.7836E+01	1.6974E+01
	Std	1.8206E−01	1.4733E−01	1.2652E−01	2.4615E−01	1.9114E−01	1.8381E−01	2.3611E−01	7.0197E+00	2.4848E−01	3.6928E+00	3.0791E−01
	Rank	1	4	2	8	7	5	3	11	6	10	9
Friedman Rank		1.143	3.429	3.286	6.571	8.857	5.857	7.000	8.714	5.143	9.571	6.429
Wilcoxon rank−sum test (+/=/−)		N/A	6/1/0	6/1/0	7/0/0	7/0/0	7/0/0	7/0/0	7/0/0	7/0/0	7/0/0	6/1/0

## Data Availability

The data are provided within the manuscript.

## Appendix A

**Table A1:** Experimental results of ERBMO with different *P* (D = 10).

No.	Index	RBMO	P = 5D	P = 10D	P = 15D	P = 20D	P = 25D	P = 30D
F1	Best	4.8595E+04	4.8595E+04	1.0763E+02	9.4699E+02	3.9914E+03	1.9278E+04	8.4037E+03
	Avg	1.9123E+06	1.9123E+06	6.1492E+03	1.7517E+04	4.8399E+05	4.9668E+05	7.9832E+06
	Std	4.3461E+06	4.3461E+06	7.6330E+03	4.2886E+04	7.7810E+05	5.5473E+05	9.6802E+06
	Rank	5	5	1	2	3	4	6
F2	Best	3.0242E+02	3.0242E+02	3.0000E+02	3.0001E+02	3.0024E+02	3.0190E+02	3.0086E+02
	Avg	3.0229E+03	3.0229E+03	4.0358E+02	3.0347E+02	7.9393E+02	3.5153E+02	3.9217E+02
	Std	3.7665E+03	3.7665E+03	2.3704E+02	1.0456E+01	1.8443E+03	1.2587E+02	1.4431E+02
	Rank	6	6	4	1	5	2	3
F3	Best	4.0058E+02	4.0058E+02	4.0180E+02	4.0346E+02	4.0486E+02	4.0285E+02	4.0504E+02
	Avg	4.1157E+02	4.1157E+02	4.0516E+02	4.0503E+02	4.0560E+02	4.0605E+02	4.0717E+02
	Std	2.4411E+01	2.4411E+01	1.8355E+00	9.7697E−01	6.1410E−01	1.3397E+00	2.2543E+00
	Rank	6	6	2	1	3	4	5
F4	Best	5.0612E+02	5.0612E+02	5.0301E+02	5.1582E+02	5.1504E+02	5.0594E+02	5.1237E+02
	Avg	5.2157E+02	5.2157E+02	5.1216E+02	5.2575E+02	5.2907E+02	5.2476E+02	5.2828E+02
	Std	8.6368E+00	8.6368E+00	6.4400E+00	5.6800E+00	7.4053E+00	7.5196E+00	9.2499E+00
	Rank	2	2	1	4	6	3	5
F5	Best	6.0295E+02	6.0295E+02	6.0001E+02	6.0018E+02	6.0081E+02	6.0061E+02	6.0202E+02
	Avg	6.0909E+02	6.0909E+02	6.0202E+02	6.0043E+02	6.0183E+02	6.0218E+02	6.0454E+02
	Std	4.5711E+00	4.5711E+00	3.3227E+00	3.6358E−01	7.7587E−01	1.2081E+00	1.6609E+00
	Rank	6	6	3	1	2	4	5
F6	Best	7.1924E+02	7.1924E+02	7.1620E+02	7.2143E+02	7.3322E+02	7.2249E+02	7.2952E+02
	Avg	7.3863E+02	7.3863E+02	7.2902E+02	7.3799E+02	7.4284E+02	7.4257E+02	7.4279E+02
	Std	1.2719E+01	1.2719E+01	1.0369E+01	7.6167E+00	5.5781E+00	8.9606E+00	1.0005E+01
	Rank	3	3	1	2	6	4	5
F7	Best	8.0340E+02	8.0340E+02	8.0510E+02	8.1230E+02	8.1695E+02	8.0941E+02	8.2126E+02
	Avg	8.1352E+02	8.1352E+02	8.1147E+02	8.2517E+02	8.2651E+02	8.2581E+02	8.3387E+02
	Std	6.1569E+00	6.1569E+00	5.0650E+00	5.5161E+00	7.0147E+00	9.7016E+00	8.8162E+00
	Rank	2	2	1	3	5	4	6
F8	Best	9.1818E+02	9.1818E+02	9.0000E+02	9.0000E+02	9.0030E+02	9.0003E+02	9.0150E+02
	Avg	1.0713E+03	1.0713E+03	9.0940E+02	9.0073E+02	9.1313E+02	9.0675E+02	9.0810E+02
	Std	1.5316E+02	1.5316E+02	2.3728E+01	1.8366E+00	4.6510E+01	6.5648E+00	9.6271E+00
	Rank	6	6	4	1	5	2	3
F9	Best	1.4081E+03	1.4081E+03	1.6475E+03	1.7746E+03	2.1070E+03	1.9598E+03	1.5376E+03
	Avg	2.1563E+03	2.1563E+03	2.1775E+03	2.2668E+03	2.4329E+03	2.4066E+03	2.2734E+03
	Std	4.1512E+02	4.1512E+02	3.3064E+02	2.8930E+02	2.5572E+02	2.8808E+02	3.0560E+02
	Rank	1	1	2	3	6	5	4
F10	Best	1.1071E+03	1.1071E+03	1.1048E+03	1.1035E+03	1.1069E+03	1.1068E+03	1.1069E+03
	Avg	1.1681E+03	1.1681E+03	1.1226E+03	1.1155E+03	1.1132E+03	1.1257E+03	1.1241E+03
	Std	5.9867E+01	5.9867E+01	1.3765E+01	1.2798E+01	4.7963E+00	4.2826E+01	1.3995E+01
	Rank	6	6	3	2	1	5	4
F11	Best	2.3525E+03	2.3525E+03	1.3422E+03	1.5929E+03	2.8385E+03	3.6268E+03	3.6692E+03
	Avg	1.6594E+06	1.6594E+06	5.8179E+03	4.5646E+05	1.6254E+05	2.1813E+04	1.2329E+05
	Std	5.4052E+06	5.4052E+06	8.1754E+03	1.7416E+06	5.6720E+05	1.9068E+04	2.0884E+05
	Rank	6	6	1	5	4	2	3
F12	Best	1.3787E+03	1.3787E+03	1.3516E+03	1.3372E+03	1.3406E+03	1.3669E+03	1.6952E+03
	Avg	2.4121E+03	2.4121E+03	1.6045E+03	1.5285E+03	1.5196E+03	1.7626E+03	3.2664E+03
	Std	1.1576E+03	1.1576E+03	3.5796E+02	2.2942E+02	2.1092E+02	2.6638E+02	2.9867E+03
	Rank	5	5	3	2	1	4	6
F13	Best	1.4230E+03	1.4230E+03	1.4041E+03	1.4164E+03	1.4192E+03	1.4225E+03	1.4245E+03
	Avg	1.4371E+03	1.4371E+03	1.4261E+03	1.4281E+03	1.4287E+03	1.4290E+03	1.4385E+03
	Std	1.0260E+01	1.0260E+01	9.9050E+00	6.9769E+00	3.9787E+00	4.6537E+00	7.2611E+00
	Rank	5	5	1	2	3	4	6
F14	Best	1.5063E+03	1.5063E+03	1.5002E+03	1.5032E+03	1.5091E+03	1.5095E+03	1.5089E+03
	Avg	1.6518E+03	1.6518E+03	1.5285E+03	1.5204E+03	1.5216E+03	1.5234E+03	1.5526E+03
	Std	1.5990E+02	1.5990E+02	1.7736E+01	1.8269E+01	1.0545E+01	9.5723E+00	4.2394E+01
	Rank	6	6	4	1	2	3	5
F15	Best	1.6023E+03	1.6023E+03	1.6049E+03	1.6176E+03	1.6545E+03	1.6148E+03	1.6136E+03
	Avg	1.7508E+03	1.7508E+03	1.6427E+03	1.6565E+03	1.7184E+03	1.6937E+03	1.7261E+03
	Std	2.6290E+02	2.6290E+02	5.7058E+01	3.1351E+01	5.3756E+01	7.0774E+01	7.2044E+01
	Rank	6	6	1	2	4	3	5
F16	Best	1.7261E+03	1.7261E+03	1.7287E+03	1.7485E+03	1.7472E+03	1.7590E+03	1.7683E+03
	Avg	1.7576E+03	1.7576E+03	1.7640E+03	1.7701E+03	1.7858E+03	1.7780E+03	1.7929E+03
	Std	4.0995E+01	4.0995E+01	1.8687E+01	1.4271E+01	2.1839E+01	1.4121E+01	2.0678E+01
	Rank	1	1	2	3	5	4	6
F17	Best	1.8450E+03	1.8450E+03	1.8279E+03	1.8271E+03	1.8555E+03	1.9132E+03	1.9449E+03
	Avg	6.4087E+03	6.4087E+03	1.8997E+03	1.8840E+03	1.9201E+03	2.0905E+03	3.1689E+03
	Std	7.0102E+03	7.0102E+03	8.1512E+01	8.6589E+01	7.1315E+01	1.4029E+02	2.0460E+03
	Rank	6	6	2	1	3	4	5
F18	Best	1.9064E+03	1.9064E+03	1.9025E+03	1.9040E+03	1.9053E+03	1.9059E+03	1.9071E+03
	Avg	2.0149E+03	2.0149E+03	1.9076E+03	1.9061E+03	1.9073E+03	1.9097E+03	1.9189E+03
	Std	3.4349E+02	3.4349E+02	5.3305E+00	2.0609E+00	1.3595E+00	2.3615E+00	9.3895E+00
	Rank	6	6	3	1	2	4	5
F19	Best	2.0227E+03	2.0227E+03	2.0264E+03	2.0591E+03	2.0740E+03	2.0266E+03	2.0700E+03
	Avg	2.0862E+03	2.0862E+03	2.0718E+03	2.0955E+03	2.1293E+03	2.1215E+03	2.1232E+03
	Std	4.6870E+01	4.6870E+01	2.9628E+01	3.5544E+01	3.6273E+01	4.2822E+01	4.0521E+01
	Rank	2	2	1	3	6	4	5
F20	Best	2.2062E+03	2.2062E+03	2.2056E+03	2.2066E+03	2.2044E+03	2.2089E+03	2.2059E+03
	Avg	2.2872E+03	2.2872E+03	2.3081E+03	2.2943E+03	2.2911E+03	2.2939E+03	2.2802E+03
	Std	4.8298E+01	4.8298E+01	2.9635E+01	4.1245E+01	5.7039E+01	4.7961E+01	5.2861E+01
	Rank	2	2	6	5	3	4	1
F21	Best	2.2431E+03	2.2431E+03	2.3000E+03	2.2121E+03	2.2265E+03	2.2157E+03	2.2172E+03
	Avg	2.3085E+03	2.3085E+03	2.3028E+03	2.2846E+03	2.3037E+03	2.2876E+03	2.3099E+03
	Std	2.0642E+01	2.0642E+01	3.3049E+00	3.3724E+01	2.1425E+01	3.5405E+01	2.7207E+01
	Rank	5	5	3	1	4	2	6
F22	Best	2.6059E+03	2.6059E+03	2.6053E+03	2.6074E+03	2.6160E+03	2.6156E+03	2.6234E+03
	Avg	2.6319E+03	2.6319E+03	2.6192E+03	2.6225E+03	2.6286E+03	2.6301E+03	2.6329E+03
	Std	2.5433E+01	2.5433E+01	2.4610E+01	1.2546E+01	7.2161E+00	1.4344E+01	7.2166E+00
	Rank	5	5	1	2	3	4	6
F23	Best	2.7347E+03	2.7347E+03	2.7295E+03	2.6808E+03	2.5246E+03	2.6111E+03	2.5780E+03
	Avg	2.7513E+03	2.7513E+03	2.7385E+03	2.7449E+03	2.7381E+03	2.7373E+03	2.7449E+03
	Std	1.8318E+01	1.8318E+01	7.4868E+00	1.8709E+01	6.0590E+01	4.4556E+01	5.0939E+01
	Rank	6	6	3	4	2	1	5
F24	Best	2.8984E+03	2.8984E+03	2.8978E+03	2.8977E+03	2.8980E+03	2.8990E+03	2.8983E+03
	Avg	2.9514E+03	2.9514E+03	2.9346E+03	2.9286E+03	2.9213E+03	2.9340E+03	2.9360E+03
	Std	4.2770E+01	4.2770E+01	2.0191E+01	3.5185E+01	3.5084E+01	1.9936E+01	1.9592E+01
	Rank	6	6	4	2	1	3	5
F25	Best	2.9572E+03	2.9572E+03	2.9000E+03	2.9002E+03	2.9027E+03	2.9024E+03	2.9113E+03
	Avg	3.1784E+03	3.1784E+03	2.9908E+03	2.9230E+03	2.9129E+03	2.9270E+03	2.9430E+03
	Std	1.2692E+02	1.2692E+02	1.0853E+02	3.9557E+01	1.2724E+01	2.8823E+01	2.6207E+01
	Rank	6	6	5	2	1	3	4
F26	Best	3.0909E+03	3.0909E+03	3.0894E+03	3.0894E+03	3.0909E+03	3.0911E+03	3.0929E+03
	Avg	3.1142E+03	3.1142E+03	3.1005E+03	3.0991E+03	3.1041E+03	3.0970E+03	3.0994E+03
	Std	2.6483E+01	2.6483E+01	1.5908E+01	1.6515E+01	2.4439E+01	4.1964E+00	7.7354E+00
	Rank	6	6	4	2	5	1	3
F27	Best	3.2654E+03	3.2654E+03	3.1778E+03	3.1687E+03	3.1550E+03	3.1859E+03	3.3073E+03
	Avg	3.4302E+03	3.4302E+03	3.3868E+03	3.3706E+03	3.3722E+03	3.3821E+03	3.4074E+03
	Std	6.8223E+01	6.8223E+01	8.1829E+01	9.8072E+01	8.3396E+01	6.6498E+01	3.0778E+01
	Rank	6	6	4	1	2	3	5
F28	Best	3.1360E+03	3.1360E+03	3.1509E+03	3.1556E+03	3.1782E+03	3.1633E+03	3.1534E+03
	Avg	3.2112E+03	3.2112E+03	3.1934E+03	3.2025E+03	3.2137E+03	3.2209E+03	3.2122E+03
	Std	6.0581E+01	6.0581E+01	3.3077E+01	3.2331E+01	2.1876E+01	3.3264E+01	3.2422E+01
	Rank	3	3	1	2	5	6	4
F29	Best	6.2676E+03	6.2676E+03	3.6511E+03	3.7610E+03	3.7521E+03	3.6571E+03	4.0333E+03
	Avg	4.1484E+05	4.1484E+05	3.2335E+05	6.3152E+05	5.8273E+05	2.8142E+05	3.4140E+05
	Std	3.5198E+05	3.5198E+05	4.9806E+05	1.0372E+06	1.1270E+06	4.6953E+05	5.1143E+05
	Rank	4	4	2	6	5	1	3

**Table A2:** Experimental results of ERBMO with different *P* (D = 30).

No.	Index	RBMO	P = 5D	P = 10D	P = 15D	P = 20D	P = 25D	P = 30D
F1	Best	4.8595E+04	4.8595E+04	1.0763E+02	9.4699E+02	3.9914E+03	1.9278E+04	8.4037E+03
	Avg	1.9123E+06	1.9123E+06	6.1492E+03	1.7517E+04	4.8399E+05	4.9668E+05	7.9832E+06
	Std	4.3461E+06	4.3461E+06	7.6330E+03	4.2886E+04	7.7810E+05	5.5473E+05	9.6802E+06
	Rank	5	5	1	2	3	4	6
F2	Best	3.0242E+02	3.0242E+02	3.0000E+02	3.0001E+02	3.0024E+02	3.0190E+02	3.0086E+02
	Avg	3.0229E+03	3.0229E+03	4.0358E+02	3.0347E+02	7.9393E+02	3.5153E+02	3.9217E+02
	Std	3.7665E+03	3.7665E+03	2.3704E+02	1.0456E+01	1.8443E+03	1.2587E+02	1.4431E+02
	Rank	6	6	4	1	5	2	3
F3	Best	4.0058E+02	4.0058E+02	4.0180E+02	4.0346E+02	4.0486E+02	4.0285E+02	4.0504E+02
	Avg	4.1157E+02	4.1157E+02	4.0516E+02	4.0503E+02	4.0560E+02	4.0605E+02	4.0717E+02
	Std	2.4411E+01	2.4411E+01	1.8355E+00	9.7697E−01	6.1410E−01	1.3397E+00	2.2543E+00
	Rank	6	6	2	1	3	4	5
F4	Best	5.0612E+02	5.0612E+02	5.0301E+02	5.1582E+02	5.1504E+02	5.0594E+02	5.1237E+02
	Avg	5.2157E+02	5.2157E+02	5.1216E+02	5.2575E+02	5.2907E+02	5.2476E+02	5.2828E+02
	Std	8.6368E+00	8.6368E+00	6.4400E+00	5.6800E+00	7.4053E+00	7.5196E+00	9.2499E+00
	Rank	2	2	1	4	6	3	5
F5	Best	6.0295E+02	6.0295E+02	6.0001E+02	6.0018E+02	6.0081E+02	6.0061E+02	6.0202E+02
	Avg	6.0909E+02	6.0909E+02	6.0202E+02	6.0043E+02	6.0183E+02	6.0218E+02	6.0454E+02
	Std	4.5711E+00	4.5711E+00	3.3227E+00	3.6358E−01	7.7587E−01	1.2081E+00	1.6609E+00
	Rank	6	6	3	1	2	4	5
F6	Best	7.1924E+02	7.1924E+02	7.1620E+02	7.2143E+02	7.3322E+02	7.2249E+02	7.2952E+02
	Avg	7.3863E+02	7.3863E+02	7.2902E+02	7.3799E+02	7.4284E+02	7.4257E+02	7.4279E+02
	Std	1.2719E+01	1.2719E+01	1.0369E+01	7.6167E+00	5.5781E+00	8.9606E+00	1.0005E+01
	Rank	3	3	1	2	6	4	5
F7	Best	8.0340E+02	8.0340E+02	8.0510E+02	8.1230E+02	8.1695E+02	8.0941E+02	8.2126E+02
	Avg	8.1352E+02	8.1352E+02	8.1147E+02	8.2517E+02	8.2651E+02	8.2581E+02	8.3387E+02
	Std	6.1569E+00	6.1569E+00	5.0650E+00	5.5161E+00	7.0147E+00	9.7016E+00	8.8162E+00
	Rank	2	2	1	3	5	4	6
F8	Best	9.1818E+02	9.1818E+02	9.0000E+02	9.0000E+02	9.0030E+02	9.0003E+02	9.0150E+02
	Avg	1.0713E+03	1.0713E+03	9.0940E+02	9.0073E+02	9.1313E+02	9.0675E+02	9.0810E+02
	Std	1.5316E+02	1.5316E+02	2.3728E+01	1.8366E+00	4.6510E+01	6.5648E+00	9.6271E+00
	Rank	6	6	4	1	5	2	3
F9	Best	1.4081E+03	1.4081E+03	1.6475E+03	1.7746E+03	2.1070E+03	1.9598E+03	1.5376E+03
	Avg	2.1563E+03	2.1563E+03	2.1775E+03	2.2668E+03	2.4329E+03	2.4066E+03	2.2734E+03
	Std	4.1512E+02	4.1512E+02	3.3064E+02	2.8930E+02	2.5572E+02	2.8808E+02	3.0560E+02
	Rank	1	1	2	3	6	5	4
F10	Best	1.1071E+03	1.1071E+03	1.1048E+03	1.1035E+03	1.1069E+03	1.1068E+03	1.1069E+03
	Avg	1.1681E+03	1.1681E+03	1.1226E+03	1.1155E+03	1.1132E+03	1.1257E+03	1.1241E+03
	Std	5.9867E+01	5.9867E+01	1.3765E+01	1.2798E+01	4.7963E+00	4.2826E+01	1.3995E+01
	Rank	6	6	3	2	1	5	4
F11	Best	2.3525E+03	2.3525E+03	1.3422E+03	1.5929E+03	2.8385E+03	3.6268E+03	3.6692E+03
	Avg	1.6594E+06	1.6594E+06	5.8179E+03	4.5646E+05	1.6254E+05	2.1813E+04	1.2329E+05
	Std	5.4052E+06	5.4052E+06	8.1754E+03	1.7416E+06	5.6720E+05	1.9068E+04	2.0884E+05
	Rank	6	6	1	5	4	2	3
F12	Best	1.3787E+03	1.3787E+03	1.3516E+03	1.3372E+03	1.3406E+03	1.3669E+03	1.6952E+03
	Avg	2.4121E+03	2.4121E+03	1.6045E+03	1.5285E+03	1.5196E+03	1.7626E+03	3.2664E+03
	Std	1.1576E+03	1.1576E+03	3.5796E+02	2.2942E+02	2.1092E+02	2.6638E+02	2.9867E+03
	Rank	5	5	3	2	1	4	6
F13	Best	1.4230E+03	1.4230E+03	1.4041E+03	1.4164E+03	1.4192E+03	1.4225E+03	1.4245E+03
	Avg	1.4371E+03	1.4371E+03	1.4261E+03	1.4281E+03	1.4287E+03	1.4290E+03	1.4385E+03
	Std	1.0260E+01	1.0260E+01	9.9050E+00	6.9769E+00	3.9787E+00	4.6537E+00	7.2611E+00
	Rank	5	5	1	2	3	4	6
F14	Best	1.5063E+03	1.5063E+03	1.5002E+03	1.5032E+03	1.5091E+03	1.5095E+03	1.5089E+03
	Avg	1.6518E+03	1.6518E+03	1.5285E+03	1.5204E+03	1.5216E+03	1.5234E+03	1.5526E+03
	Std	1.5990E+02	1.5990E+02	1.7736E+01	1.8269E+01	1.0545E+01	9.5723E+00	4.2394E+01
	Rank	6	6	4	1	2	3	5
F15	Best	1.6023E+03	1.6023E+03	1.6049E+03	1.6176E+03	1.6545E+03	1.6148E+03	1.6136E+03
	Avg	1.7508E+03	1.7508E+03	1.6427E+03	1.6565E+03	1.7184E+03	1.6937E+03	1.7261E+03
	Std	2.6290E+02	2.6290E+02	5.7058E+01	3.1351E+01	5.3756E+01	7.0774E+01	7.2044E+01
	Rank	6	6	1	2	4	3	5
F16	Best	1.7261E+03	1.7261E+03	1.7287E+03	1.7485E+03	1.7472E+03	1.7590E+03	1.7683E+03
	Avg	1.7576E+03	1.7576E+03	1.7640E+03	1.7701E+03	1.7858E+03	1.7780E+03	1.7929E+03
	Std	4.0995E+01	4.0995E+01	1.8687E+01	1.4271E+01	2.1839E+01	1.4121E+01	2.0678E+01
	Rank	1	1	2	3	5	4	6
F17	Best	1.8450E+03	1.8450E+03	1.8279E+03	1.8271E+03	1.8555E+03	1.9132E+03	1.9449E+03
	Avg	6.4087E+03	6.4087E+03	1.8997E+03	1.8840E+03	1.9201E+03	2.0905E+03	3.1689E+03
	Std	7.0102E+03	7.0102E+03	8.1512E+01	8.6589E+01	7.1315E+01	1.4029E+02	2.0460E+03
	Rank	6	6	2	1	3	4	5
F18	Best	1.9064E+03	1.9064E+03	1.9025E+03	1.9040E+03	1.9053E+03	1.9059E+03	1.9071E+03
	Avg	2.0149E+03	2.0149E+03	1.9076E+03	1.9061E+03	1.9073E+03	1.9097E+03	1.9189E+03
	Std	3.4349E+02	3.4349E+02	5.3305E+00	2.0609E+00	1.3595E+00	2.3615E+00	9.3895E+00
	Rank	6	6	3	1	2	4	5
F19	Best	2.0227E+03	2.0227E+03	2.0264E+03	2.0591E+03	2.0740E+03	2.0266E+03	2.0700E+03
	Avg	2.0862E+03	2.0862E+03	2.0718E+03	2.0955E+03	2.1293E+03	2.1215E+03	2.1232E+03
	Std	4.6870E+01	4.6870E+01	2.9628E+01	3.5544E+01	3.6273E+01	4.2822E+01	4.0521E+01
	Rank	2	2	1	3	6	4	5
F20	Best	2.2062E+03	2.2062E+03	2.2056E+03	2.2066E+03	2.2044E+03	2.2089E+03	2.2059E+03
	Avg	2.2872E+03	2.2872E+03	2.3081E+03	2.2943E+03	2.2911E+03	2.2939E+03	2.2802E+03
	Std	4.8298E+01	4.8298E+01	2.9635E+01	4.1245E+01	5.7039E+01	4.7961E+01	5.2861E+01
	Rank	2	2	6	5	3	4	1
F21	Best	2.2431E+03	2.2431E+03	2.3000E+03	2.2121E+03	2.2265E+03	2.2157E+03	2.2172E+03
	Avg	2.3085E+03	2.3085E+03	2.3028E+03	2.2846E+03	2.3037E+03	2.2876E+03	2.3099E+03
	Std	2.0642E+01	2.0642E+01	3.3049E+00	3.3724E+01	2.1425E+01	3.5405E+01	2.7207E+01
	Rank	5	5	3	1	4	2	6
F22	Best	2.6059E+03	2.6059E+03	2.6053E+03	2.6074E+03	2.6160E+03	2.6156E+03	2.6234E+03
	Avg	2.6319E+03	2.6319E+03	2.6192E+03	2.6225E+03	2.6286E+03	2.6301E+03	2.6329E+03
	Std	2.5433E+01	2.5433E+01	2.4610E+01	1.2546E+01	7.2161E+00	1.4344E+01	7.2166E+00
	Rank	5	5	1	2	3	4	6
F23	Best	2.7347E+03	2.7347E+03	2.7295E+03	2.6808E+03	2.5246E+03	2.6111E+03	2.5780E+03
	Avg	2.7513E+03	2.7513E+03	2.7385E+03	2.7449E+03	2.7381E+03	2.7373E+03	2.7449E+03
	Std	1.8318E+01	1.8318E+01	7.4868E+00	1.8709E+01	6.0590E+01	4.4556E+01	5.0939E+01
	Rank	6	6	3	4	2	1	5
F24	Best	2.8984E+03	2.8984E+03	2.8978E+03	2.8977E+03	2.8980E+03	2.8990E+03	2.8983E+03
	Avg	2.9514E+03	2.9514E+03	2.9346E+03	2.9286E+03	2.9213E+03	2.9340E+03	2.9360E+03
	Std	4.2770E+01	4.2770E+01	2.0191E+01	3.5185E+01	3.5084E+01	1.9936E+01	1.9592E+01
	Rank	6	6	4	2	1	3	5
F25	Best	2.9572E+03	2.9572E+03	2.9000E+03	2.9002E+03	2.9027E+03	2.9024E+03	2.9113E+03
	Avg	3.1784E+03	3.1784E+03	2.9908E+03	2.9230E+03	2.9129E+03	2.9270E+03	2.9430E+03
	Std	1.2692E+02	1.2692E+02	1.0853E+02	3.9557E+01	1.2724E+01	2.8823E+01	2.6207E+01
	Rank	6	6	5	2	1	3	4
F26	Best	3.0909E+03	3.0909E+03	3.0894E+03	3.0894E+03	3.0909E+03	3.0911E+03	3.0929E+03
	Avg	3.1142E+03	3.1142E+03	3.1005E+03	3.0991E+03	3.1041E+03	3.0970E+03	3.0994E+03
	Std	2.6483E+01	2.6483E+01	1.5908E+01	1.6515E+01	2.4439E+01	4.1964E+00	7.7354E+00
	Rank	6	6	4	2	5	1	3
F27	Best	3.2654E+03	3.2654E+03	3.1778E+03	3.1687E+03	3.1550E+03	3.1859E+03	3.3073E+03
	Avg	3.4302E+03	3.4302E+03	3.3868E+03	3.3706E+03	3.3722E+03	3.3821E+03	3.4074E+03
	Std	6.8223E+01	6.8223E+01	8.1829E+01	9.8072E+01	8.3396E+01	6.6498E+01	3.0778E+01
	Rank	6	6	4	1	2	3	5
F28	Best	3.1360E+03	3.1360E+03	3.1509E+03	3.1556E+03	3.1782E+03	3.1633E+03	3.1534E+03
	Avg	3.2112E+03	3.2112E+03	3.1934E+03	3.2025E+03	3.2137E+03	3.2209E+03	3.2122E+03
	Std	6.0581E+01	6.0581E+01	3.3077E+01	3.2331E+01	2.1876E+01	3.3264E+01	3.2422E+01
	Rank	3	3	1	2	5	6	4
F29	Best	6.2676E+03	6.2676E+03	3.6511E+03	3.7610E+03	3.7521E+03	3.6571E+03	4.0333E+03
	Avg	4.1484E+05	4.1484E+05	3.2335E+05	6.3152E+05	5.8273E+05	2.8142E+05	3.4140E+05
	Std	3.5198E+05	3.5198E+05	4.9806E+05	1.0372E+06	1.1270E+06	4.6953E+05	5.1143E+05
	Rank	4	4	2	6	5	1	3

**Table A3:** Experimental results of ERBMO with different *P* (D = 50).

No.	Index	RBMO	P = 5D	P = 10D	P = 15D	P = 20D	P = 25D	P = 30D
F1	Best	4.8595E+04	4.8595E+04	1.0763E+02	9.4699E+02	3.9914E+03	1.9278E+04	8.4037E+03
	Avg	1.9123E+06	1.9123E+06	6.1492E+03	1.7517E+04	4.8399E+05	4.9668E+05	7.9832E+06
	Std	4.3461E+06	4.3461E+06	7.6330E+03	4.2886E+04	7.7810E+05	5.5473E+05	9.6802E+06
	Rank	5	5	1	2	3	4	6
F2	Best	3.0242E+02	3.0242E+02	3.0000E+02	3.0001E+02	3.0024E+02	3.0190E+02	3.0086E+02
	Avg	3.0229E+03	3.0229E+03	4.0358E+02	3.0347E+02	7.9393E+02	3.5153E+02	3.9217E+02
	Std	3.7665E+03	3.7665E+03	2.3704E+02	1.0456E+01	1.8443E+03	1.2587E+02	1.4431E+02
	Rank	6	6	4	1	5	2	3
F3	Best	4.0058E+02	4.0058E+02	4.0180E+02	4.0346E+02	4.0486E+02	4.0285E+02	4.0504E+02
	Avg	4.1157E+02	4.1157E+02	4.0516E+02	4.0503E+02	4.0560E+02	4.0605E+02	4.0717E+02
	Std	2.4411E+01	2.4411E+01	1.8355E+00	9.7697E−01	6.1410E−01	1.3397E+00	2.2543E+00
	Rank	6	6	2	1	3	4	5
F4	Best	5.0612E+02	5.0612E+02	5.0301E+02	5.1582E+02	5.1504E+02	5.0594E+02	5.1237E+02
	Avg	5.2157E+02	5.2157E+02	5.1216E+02	5.2575E+02	5.2907E+02	5.2476E+02	5.2828E+02
	Std	8.6368E+00	8.6368E+00	6.4400E+00	5.6800E+00	7.4053E+00	7.5196E+00	9.2499E+00
	Rank	2	2	1	4	6	3	5
F5	Best	6.0295E+02	6.0295E+02	6.0001E+02	6.0018E+02	6.0081E+02	6.0061E+02	6.0202E+02
	Avg	6.0909E+02	6.0909E+02	6.0202E+02	6.0043E+02	6.0183E+02	6.0218E+02	6.0454E+02
	Std	4.5711E+00	4.5711E+00	3.3227E+00	3.6358E−01	7.7587E−01	1.2081E+00	1.6609E+00
	Rank	6	6	3	1	2	4	5
F6	Best	7.1924E+02	7.1924E+02	7.1620E+02	7.2143E+02	7.3322E+02	7.2249E+02	7.2952E+02
	Avg	7.3863E+02	7.3863E+02	7.2902E+02	7.3799E+02	7.4284E+02	7.4257E+02	7.4279E+02
	Std	1.2719E+01	1.2719E+01	1.0369E+01	7.6167E+00	5.5781E+00	8.9606E+00	1.0005E+01
	Rank	3	3	1	2	6	4	5
F7	Best	8.0340E+02	8.0340E+02	8.0510E+02	8.1230E+02	8.1695E+02	8.0941E+02	8.2126E+02
	Avg	8.1352E+02	8.1352E+02	8.1147E+02	8.2517E+02	8.2651E+02	8.2581E+02	8.3387E+02
	Std	6.1569E+00	6.1569E+00	5.0650E+00	5.5161E+00	7.0147E+00	9.7016E+00	8.8162E+00
	Rank	2	2	1	3	5	4	6
F8	Best	9.1818E+02	9.1818E+02	9.0000E+02	9.0000E+02	9.0030E+02	9.0003E+02	9.0150E+02
	Avg	1.0713E+03	1.0713E+03	9.0940E+02	9.0073E+02	9.1313E+02	9.0675E+02	9.0810E+02
	Std	1.5316E+02	1.5316E+02	2.3728E+01	1.8366E+00	4.6510E+01	6.5648E+00	9.6271E+00
	Rank	6	6	4	1	5	2	3
F9	Best	1.4081E+03	1.4081E+03	1.6475E+03	1.7746E+03	2.1070E+03	1.9598E+03	1.5376E+03
	Avg	2.1563E+03	2.1563E+03	2.1775E+03	2.2668E+03	2.4329E+03	2.4066E+03	2.2734E+03
	Std	4.1512E+02	4.1512E+02	3.3064E+02	2.8930E+02	2.5572E+02	2.8808E+02	3.0560E+02
	Rank	1	1	2	3	6	5	4
F10	Best	1.1071E+03	1.1071E+03	1.1048E+03	1.1035E+03	1.1069E+03	1.1068E+03	1.1069E+03
	Avg	1.1681E+03	1.1681E+03	1.1226E+03	1.1155E+03	1.1132E+03	1.1257E+03	1.1241E+03
	Std	5.9867E+01	5.9867E+01	1.3765E+01	1.2798E+01	4.7963E+00	4.2826E+01	1.3995E+01
	Rank	6	6	3	2	1	5	4
F11	Best	2.3525E+03	2.3525E+03	1.3422E+03	1.5929E+03	2.8385E+03	3.6268E+03	3.6692E+03
	Avg	1.6594E+06	1.6594E+06	5.8179E+03	4.5646E+05	1.6254E+05	2.1813E+04	1.2329E+05
	Std	5.4052E+06	5.4052E+06	8.1754E+03	1.7416E+06	5.6720E+05	1.9068E+04	2.0884E+05
	Rank	6	6	1	5	4	2	3
F12	Best	1.3787E+03	1.3787E+03	1.3516E+03	1.3372E+03	1.3406E+03	1.3669E+03	1.6952E+03
	Avg	2.4121E+03	2.4121E+03	1.6045E+03	1.5285E+03	1.5196E+03	1.7626E+03	3.2664E+03
	Std	1.1576E+03	1.1576E+03	3.5796E+02	2.2942E+02	2.1092E+02	2.6638E+02	2.9867E+03
	Rank	5	5	3	2	1	4	6
F13	Best	1.4230E+03	1.4230E+03	1.4041E+03	1.4164E+03	1.4192E+03	1.4225E+03	1.4245E+03
	Avg	1.4371E+03	1.4371E+03	1.4261E+03	1.4281E+03	1.4287E+03	1.4290E+03	1.4385E+03
	Std	1.0260E+01	1.0260E+01	9.9050E+00	6.9769E+00	3.9787E+00	4.6537E+00	7.2611E+00
	Rank	5	5	1	2	3	4	6
F14	Best	1.5063E+03	1.5063E+03	1.5002E+03	1.5032E+03	1.5091E+03	1.5095E+03	1.5089E+03
	Avg	1.6518E+03	1.6518E+03	1.5285E+03	1.5204E+03	1.5216E+03	1.5234E+03	1.5526E+03
	Std	1.5990E+02	1.5990E+02	1.7736E+01	1.8269E+01	1.0545E+01	9.5723E+00	4.2394E+01
	Rank	6	6	4	1	2	3	5
F15	Best	1.6023E+03	1.6023E+03	1.6049E+03	1.6176E+03	1.6545E+03	1.6148E+03	1.6136E+03
	Avg	1.7508E+03	1.7508E+03	1.6427E+03	1.6565E+03	1.7184E+03	1.6937E+03	1.7261E+03
	Std	2.6290E+02	2.6290E+02	5.7058E+01	3.1351E+01	5.3756E+01	7.0774E+01	7.2044E+01
	Rank	6	6	1	2	4	3	5
F16	Best	1.7261E+03	1.7261E+03	1.7287E+03	1.7485E+03	1.7472E+03	1.7590E+03	1.7683E+03
	Avg	1.7576E+03	1.7576E+03	1.7640E+03	1.7701E+03	1.7858E+03	1.7780E+03	1.7929E+03
	Std	4.0995E+01	4.0995E+01	1.8687E+01	1.4271E+01	2.1839E+01	1.4121E+01	2.0678E+01
	Rank	1	1	2	3	5	4	6
F17	Best	1.8450E+03	1.8450E+03	1.8279E+03	1.8271E+03	1.8555E+03	1.9132E+03	1.9449E+03
	Avg	6.4087E+03	6.4087E+03	1.8997E+03	1.8840E+03	1.9201E+03	2.0905E+03	3.1689E+03
	Std	7.0102E+03	7.0102E+03	8.1512E+01	8.6589E+01	7.1315E+01	1.4029E+02	2.0460E+03
	Rank	6	6	2	1	3	4	5
F18	Best	1.9064E+03	1.9064E+03	1.9025E+03	1.9040E+03	1.9053E+03	1.9059E+03	1.9071E+03
	Avg	2.0149E+03	2.0149E+03	1.9076E+03	1.9061E+03	1.9073E+03	1.9097E+03	1.9189E+03
	Std	3.4349E+02	3.4349E+02	5.3305E+00	2.0609E+00	1.3595E+00	2.3615E+00	9.3895E+00
	Rank	6	6	3	1	2	4	5
F19	Best	2.0227E+03	2.0227E+03	2.0264E+03	2.0591E+03	2.0740E+03	2.0266E+03	2.0700E+03
	Avg	2.0862E+03	2.0862E+03	2.0718E+03	2.0955E+03	2.1293E+03	2.1215E+03	2.1232E+03
	Std	4.6870E+01	4.6870E+01	2.9628E+01	3.5544E+01	3.6273E+01	4.2822E+01	4.0521E+01
	Rank	2	2	1	3	6	4	5
F20	Best	2.2062E+03	2.2062E+03	2.2056E+03	2.2066E+03	2.2044E+03	2.2089E+03	2.2059E+03
	Avg	2.2872E+03	2.2872E+03	2.3081E+03	2.2943E+03	2.2911E+03	2.2939E+03	2.2802E+03
	Std	4.8298E+01	4.8298E+01	2.9635E+01	4.1245E+01	5.7039E+01	4.7961E+01	5.2861E+01
	Rank	2	2	6	5	3	4	1
F21	Best	2.2431E+03	2.2431E+03	2.3000E+03	2.2121E+03	2.2265E+03	2.2157E+03	2.2172E+03
	Avg	2.3085E+03	2.3085E+03	2.3028E+03	2.2846E+03	2.3037E+03	2.2876E+03	2.3099E+03
	Std	2.0642E+01	2.0642E+01	3.3049E+00	3.3724E+01	2.1425E+01	3.5405E+01	2.7207E+01
	Rank	5	5	3	1	4	2	6
F22	Best	2.6059E+03	2.6059E+03	2.6053E+03	2.6074E+03	2.6160E+03	2.6156E+03	2.6234E+03
	Avg	2.6319E+03	2.6319E+03	2.6192E+03	2.6225E+03	2.6286E+03	2.6301E+03	2.6329E+03
	Std	2.5433E+01	2.5433E+01	2.4610E+01	1.2546E+01	7.2161E+00	1.4344E+01	7.2166E+00
	Rank	5	5	1	2	3	4	6
F23	Best	2.7347E+03	2.7347E+03	2.7295E+03	2.6808E+03	2.5246E+03	2.6111E+03	2.5780E+03
	Avg	2.7513E+03	2.7513E+03	2.7385E+03	2.7449E+03	2.7381E+03	2.7373E+03	2.7449E+03
	Std	1.8318E+01	1.8318E+01	7.4868E+00	1.8709E+01	6.0590E+01	4.4556E+01	5.0939E+01
	Rank	6	6	3	4	2	1	5
F24	Best	2.8984E+03	2.8984E+03	2.8978E+03	2.8977E+03	2.8980E+03	2.8990E+03	2.8983E+03
	Avg	2.9514E+03	2.9514E+03	2.9346E+03	2.9286E+03	2.9213E+03	2.9340E+03	2.9360E+03
	Std	4.2770E+01	4.2770E+01	2.0191E+01	3.5185E+01	3.5084E+01	1.9936E+01	1.9592E+01
	Rank	6	6	4	2	1	3	5
F25	Best	2.9572E+03	2.9572E+03	2.9000E+03	2.9002E+03	2.9027E+03	2.9024E+03	2.9113E+03
	Avg	3.1784E+03	3.1784E+03	2.9908E+03	2.9230E+03	2.9129E+03	2.9270E+03	2.9430E+03
	Std	1.2692E+02	1.2692E+02	1.0853E+02	3.9557E+01	1.2724E+01	2.8823E+01	2.6207E+01
	Rank	6	6	5	2	1	3	4
F26	Best	3.0909E+03	3.0909E+03	3.0894E+03	3.0894E+03	3.0909E+03	3.0911E+03	3.0929E+03
	Avg	3.1142E+03	3.1142E+03	3.1005E+03	3.0991E+03	3.1041E+03	3.0970E+03	3.0994E+03
	Std	2.6483E+01	2.6483E+01	1.5908E+01	1.6515E+01	2.4439E+01	4.1964E+00	7.7354E+00
	Rank	6	6	4	2	5	1	3
F27	Best	3.2654E+03	3.2654E+03	3.1778E+03	3.1687E+03	3.1550E+03	3.1859E+03	3.3073E+03
	Avg	3.4302E+03	3.4302E+03	3.3868E+03	3.3706E+03	3.3722E+03	3.3821E+03	3.4074E+03
	Std	6.8223E+01	6.8223E+01	8.1829E+01	9.8072E+01	8.3396E+01	6.6498E+01	3.0778E+01
	Rank	6	6	4	1	2	3	5
F28	Best	3.1360E+03	3.1360E+03	3.1509E+03	3.1556E+03	3.1782E+03	3.1633E+03	3.1534E+03
	Avg	3.2112E+03	3.2112E+03	3.1934E+03	3.2025E+03	3.2137E+03	3.2209E+03	3.2122E+03
	Std	6.0581E+01	6.0581E+01	3.3077E+01	3.2331E+01	2.1876E+01	3.3264E+01	3.2422E+01
	Rank	3	3	1	2	5	6	4
F29	Best	6.2676E+03	6.2676E+03	3.6511E+03	3.7610E+03	3.7521E+03	3.6571E+03	4.0333E+03
	Avg	4.1484E+05	4.1484E+05	3.2335E+05	6.3152E+05	5.8273E+05	2.8142E+05	3.4140E+05
	Std	3.5198E+05	3.5198E+05	4.9806E+05	1.0372E+06	1.1270E+06	4.6953E+05	5.1143E+05
	Rank	4	4	2	6	5	1	3

**Table A4:** Experimental results of ERBMO with different *P* (D = 100).

No.	Index	RBMO	P = 5D	P = 10D	P = 15D	P = 20D	P = 25D	P = 30D
F1	Best	4.8595E+04	4.8595E+04	1.0763E+02	9.4699E+02	3.9914E+03	1.9278E+04	8.4037E+03
	Avg	1.9123E+06	1.9123E+06	6.1492E+03	1.7517E+04	4.8399E+05	4.9668E+05	7.9832E+06
	Std	4.3461E+06	4.3461E+06	7.6330E+03	4.2886E+04	7.7810E+05	5.5473E+05	9.6802E+06
	Rank	5	5	1	2	3	4	6
F2	Best	3.0242E+02	3.0242E+02	3.0000E+02	3.0001E+02	3.0024E+02	3.0190E+02	3.0086E+02
	Avg	3.0229E+03	3.0229E+03	4.0358E+02	3.0347E+02	7.9393E+02	3.5153E+02	3.9217E+02
	Std	3.7665E+03	3.7665E+03	2.3704E+02	1.0456E+01	1.8443E+03	1.2587E+02	1.4431E+02
	Rank	6	6	4	1	5	2	3
F3	Best	4.0058E+02	4.0058E+02	4.0180E+02	4.0346E+02	4.0486E+02	4.0285E+02	4.0504E+02
	Avg	4.1157E+02	4.1157E+02	4.0516E+02	4.0503E+02	4.0560E+02	4.0605E+02	4.0717E+02
	Std	2.4411E+01	2.4411E+01	1.8355E+00	9.7697E−01	6.1410E−01	1.3397E+00	2.2543E+00
	Rank	6	6	2	1	3	4	5
F4	Best	5.0612E+02	5.0612E+02	5.0301E+02	5.1582E+02	5.1504E+02	5.0594E+02	5.1237E+02
	Avg	5.2157E+02	5.2157E+02	5.1216E+02	5.2575E+02	5.2907E+02	5.2476E+02	5.2828E+02
	Std	8.6368E+00	8.6368E+00	6.4400E+00	5.6800E+00	7.4053E+00	7.5196E+00	9.2499E+00
	Rank	2	2	1	4	6	3	5
F5	Best	6.0295E+02	6.0295E+02	6.0001E+02	6.0018E+02	6.0081E+02	6.0061E+02	6.0202E+02
	Avg	6.0909E+02	6.0909E+02	6.0202E+02	6.0043E+02	6.0183E+02	6.0218E+02	6.0454E+02
	Std	4.5711E+00	4.5711E+00	3.3227E+00	3.6358E−01	7.7587E−01	1.2081E+00	1.6609E+00
	Rank	6	6	3	1	2	4	5
F6	Best	7.1924E+02	7.1924E+02	7.1620E+02	7.2143E+02	7.3322E+02	7.2249E+02	7.2952E+02
	Avg	7.3863E+02	7.3863E+02	7.2902E+02	7.3799E+02	7.4284E+02	7.4257E+02	7.4279E+02
	Std	1.2719E+01	1.2719E+01	1.0369E+01	7.6167E+00	5.5781E+00	8.9606E+00	1.0005E+01
	Rank	3	3	1	2	6	4	5
F7	Best	8.0340E+02	8.0340E+02	8.0510E+02	8.1230E+02	8.1695E+02	8.0941E+02	8.2126E+02
	Avg	8.1352E+02	8.1352E+02	8.1147E+02	8.2517E+02	8.2651E+02	8.2581E+02	8.3387E+02
	Std	6.1569E+00	6.1569E+00	5.0650E+00	5.5161E+00	7.0147E+00	9.7016E+00	8.8162E+00
	Rank	2	2	1	3	5	4	6
F8	Best	9.1818E+02	9.1818E+02	9.0000E+02	9.0000E+02	9.0030E+02	9.0003E+02	9.0150E+02
	Avg	1.0713E+03	1.0713E+03	9.0940E+02	9.0073E+02	9.1313E+02	9.0675E+02	9.0810E+02
	Std	1.5316E+02	1.5316E+02	2.3728E+01	1.8366E+00	4.6510E+01	6.5648E+00	9.6271E+00
	Rank	6	6	4	1	5	2	3
F9	Best	1.4081E+03	1.4081E+03	1.6475E+03	1.7746E+03	2.1070E+03	1.9598E+03	1.5376E+03
	Avg	2.1563E+03	2.1563E+03	2.1775E+03	2.2668E+03	2.4329E+03	2.4066E+03	2.2734E+03
	Std	4.1512E+02	4.1512E+02	3.3064E+02	2.8930E+02	2.5572E+02	2.8808E+02	3.0560E+02
	Rank	1	1	2	3	6	5	4
F10	Best	1.1071E+03	1.1071E+03	1.1048E+03	1.1035E+03	1.1069E+03	1.1068E+03	1.1069E+03
	Avg	1.1681E+03	1.1681E+03	1.1226E+03	1.1155E+03	1.1132E+03	1.1257E+03	1.1241E+03
	Std	5.9867E+01	5.9867E+01	1.3765E+01	1.2798E+01	4.7963E+00	4.2826E+01	1.3995E+01
	Rank	6	6	3	2	1	5	4
F11	Best	2.3525E+03	2.3525E+03	1.3422E+03	1.5929E+03	2.8385E+03	3.6268E+03	3.6692E+03
	Avg	1.6594E+06	1.6594E+06	5.8179E+03	4.5646E+05	1.6254E+05	2.1813E+04	1.2329E+05
	Std	5.4052E+06	5.4052E+06	8.1754E+03	1.7416E+06	5.6720E+05	1.9068E+04	2.0884E+05
	Rank	6	6	1	5	4	2	3
F12	Best	1.3787E+03	1.3787E+03	1.3516E+03	1.3372E+03	1.3406E+03	1.3669E+03	1.6952E+03
	Avg	2.4121E+03	2.4121E+03	1.6045E+03	1.5285E+03	1.5196E+03	1.7626E+03	3.2664E+03
	Std	1.1576E+03	1.1576E+03	3.5796E+02	2.2942E+02	2.1092E+02	2.6638E+02	2.9867E+03
	Rank	5	5	3	2	1	4	6
F13	Best	1.4230E+03	1.4230E+03	1.4041E+03	1.4164E+03	1.4192E+03	1.4225E+03	1.4245E+03
	Avg	1.4371E+03	1.4371E+03	1.4261E+03	1.4281E+03	1.4287E+03	1.4290E+03	1.4385E+03
	Std	1.0260E+01	1.0260E+01	9.9050E+00	6.9769E+00	3.9787E+00	4.6537E+00	7.2611E+00
	Rank	5	5	1	2	3	4	6
F14	Best	1.5063E+03	1.5063E+03	1.5002E+03	1.5032E+03	1.5091E+03	1.5095E+03	1.5089E+03
	Avg	1.6518E+03	1.6518E+03	1.5285E+03	1.5204E+03	1.5216E+03	1.5234E+03	1.5526E+03
	Std	1.5990E+02	1.5990E+02	1.7736E+01	1.8269E+01	1.0545E+01	9.5723E+00	4.2394E+01
	Rank	6	6	4	1	2	3	5
F15	Best	1.6023E+03	1.6023E+03	1.6049E+03	1.6176E+03	1.6545E+03	1.6148E+03	1.6136E+03
	Avg	1.7508E+03	1.7508E+03	1.6427E+03	1.6565E+03	1.7184E+03	1.6937E+03	1.7261E+03
	Std	2.6290E+02	2.6290E+02	5.7058E+01	3.1351E+01	5.3756E+01	7.0774E+01	7.2044E+01
	Rank	6	6	1	2	4	3	5
F16	Best	1.7261E+03	1.7261E+03	1.7287E+03	1.7485E+03	1.7472E+03	1.7590E+03	1.7683E+03
	Avg	1.7576E+03	1.7576E+03	1.7640E+03	1.7701E+03	1.7858E+03	1.7780E+03	1.7929E+03
	Std	4.0995E+01	4.0995E+01	1.8687E+01	1.4271E+01	2.1839E+01	1.4121E+01	2.0678E+01
	Rank	1	1	2	3	5	4	6
F17	Best	1.8450E+03	1.8450E+03	1.8279E+03	1.8271E+03	1.8555E+03	1.9132E+03	1.9449E+03
	Avg	6.4087E+03	6.4087E+03	1.8997E+03	1.8840E+03	1.9201E+03	2.0905E+03	3.1689E+03
	Std	7.0102E+03	7.0102E+03	8.1512E+01	8.6589E+01	7.1315E+01	1.4029E+02	2.0460E+03
	Rank	6	6	2	1	3	4	5
F18	Best	1.9064E+03	1.9064E+03	1.9025E+03	1.9040E+03	1.9053E+03	1.9059E+03	1.9071E+03
	Avg	2.0149E+03	2.0149E+03	1.9076E+03	1.9061E+03	1.9073E+03	1.9097E+03	1.9189E+03
	Std	3.4349E+02	3.4349E+02	5.3305E+00	2.0609E+00	1.3595E+00	2.3615E+00	9.3895E+00
	Rank	6	6	3	1	2	4	5
F19	Best	2.0227E+03	2.0227E+03	2.0264E+03	2.0591E+03	2.0740E+03	2.0266E+03	2.0700E+03
	Avg	2.0862E+03	2.0862E+03	2.0718E+03	2.0955E+03	2.1293E+03	2.1215E+03	2.1232E+03
	Std	4.6870E+01	4.6870E+01	2.9628E+01	3.5544E+01	3.6273E+01	4.2822E+01	4.0521E+01
	Rank	2	2	1	3	6	4	5
F20	Best	2.2062E+03	2.2062E+03	2.2056E+03	2.2066E+03	2.2044E+03	2.2089E+03	2.2059E+03
	Avg	2.2872E+03	2.2872E+03	2.3081E+03	2.2943E+03	2.2911E+03	2.2939E+03	2.2802E+03
	Std	4.8298E+01	4.8298E+01	2.9635E+01	4.1245E+01	5.7039E+01	4.7961E+01	5.2861E+01
	Rank	2	2	6	5	3	4	1
F21	Best	2.2431E+03	2.2431E+03	2.3000E+03	2.2121E+03	2.2265E+03	2.2157E+03	2.2172E+03
	Avg	2.3085E+03	2.3085E+03	2.3028E+03	2.2846E+03	2.3037E+03	2.2876E+03	2.3099E+03
	Std	2.0642E+01	2.0642E+01	3.3049E+00	3.3724E+01	2.1425E+01	3.5405E+01	2.7207E+01
	Rank	5	5	3	1	4	2	6
F22	Best	2.6059E+03	2.6059E+03	2.6053E+03	2.6074E+03	2.6160E+03	2.6156E+03	2.6234E+03
	Avg	2.6319E+03	2.6319E+03	2.6192E+03	2.6225E+03	2.6286E+03	2.6301E+03	2.6329E+03
	Std	2.5433E+01	2.5433E+01	2.4610E+01	1.2546E+01	7.2161E+00	1.4344E+01	7.2166E+00
	Rank	5	5	1	2	3	4	6
F23	Best	2.7347E+03	2.7347E+03	2.7295E+03	2.6808E+03	2.5246E+03	2.6111E+03	2.5780E+03
	Avg	2.7513E+03	2.7513E+03	2.7385E+03	2.7449E+03	2.7381E+03	2.7373E+03	2.7449E+03
	Std	1.8318E+01	1.8318E+01	7.4868E+00	1.8709E+01	6.0590E+01	4.4556E+01	5.0939E+01
	Rank	6	6	3	4	2	1	5
F24	Best	2.8984E+03	2.8984E+03	2.8978E+03	2.8977E+03	2.8980E+03	2.8990E+03	2.8983E+03
	Avg	2.9514E+03	2.9514E+03	2.9346E+03	2.9286E+03	2.9213E+03	2.9340E+03	2.9360E+03
	Std	4.2770E+01	4.2770E+01	2.0191E+01	3.5185E+01	3.5084E+01	1.9936E+01	1.9592E+01
	Rank	6	6	4	2	1	3	5
F25	Best	2.9572E+03	2.9572E+03	2.9000E+03	2.9002E+03	2.9027E+03	2.9024E+03	2.9113E+03
	Avg	3.1784E+03	3.1784E+03	2.9908E+03	2.9230E+03	2.9129E+03	2.9270E+03	2.9430E+03
	Std	1.2692E+02	1.2692E+02	1.0853E+02	3.9557E+01	1.2724E+01	2.8823E+01	2.6207E+01
	Rank	6	6	5	2	1	3	4
F26	Best	3.0909E+03	3.0909E+03	3.0894E+03	3.0894E+03	3.0909E+03	3.0911E+03	3.0929E+03
	Avg	3.1142E+03	3.1142E+03	3.1005E+03	3.0991E+03	3.1041E+03	3.0970E+03	3.0994E+03
	Std	2.6483E+01	2.6483E+01	1.5908E+01	1.6515E+01	2.4439E+01	4.1964E+00	7.7354E+00
	Rank	6	6	4	2	5	1	3
F27	Best	3.2654E+03	3.2654E+03	3.1778E+03	3.1687E+03	3.1550E+03	3.1859E+03	3.3073E+03
	Avg	3.4302E+03	3.4302E+03	3.3868E+03	3.3706E+03	3.3722E+03	3.3821E+03	3.4074E+03
	Std	6.8223E+01	6.8223E+01	8.1829E+01	9.8072E+01	8.3396E+01	6.6498E+01	3.0778E+01
	Rank	6	6	4	1	2	3	5
F28	Best	3.1360E+03	3.1360E+03	3.1509E+03	3.1556E+03	3.1782E+03	3.1633E+03	3.1534E+03
	Avg	3.2112E+03	3.2112E+03	3.1934E+03	3.2025E+03	3.2137E+03	3.2209E+03	3.2122E+03
	Std	6.0581E+01	6.0581E+01	3.3077E+01	3.2331E+01	2.1876E+01	3.3264E+01	3.2422E+01
	Rank	3	3	1	2	5	6	4
F29	Best	6.2676E+03	6.2676E+03	3.6511E+03	3.7610E+03	3.7521E+03	3.6571E+03	4.0333E+03
	Avg	4.1484E+05	4.1484E+05	3.2335E+05	6.3152E+05	5.8273E+05	2.8142E+05	3.4140E+05
	Std	3.5198E+05	3.5198E+05	4.9806E+05	1.0372E+06	1.1270E+06	4.6953E+05	5.1143E+05
	Rank	4	4	2	6	5	1	3

**Table A5:** Experimental results of ERBMO with different strategies (D = 10).

No.	Index	RBMO	DRBMO	PRBMO	ERBMO
F1	Min	7.1002E+03	1.1708E+02	1.0000E+02	1.0000E+02
	Avg	1.1200E+05	1.6527E+03	1.0017E+02	1.0004E+02
	Std	1.4890E+05	1.7843E+03	9.3064E−01	2.4024E−01
	Rank	4	3	2	1
F2	Best	3.0218E+02	3.0000E+02	3.0000E+02	3.0000E+02
	Avg	4.7568E+02	3.0001E+02	3.0000E+02	3.0000E+02
	Std	1.2889E+02	1.1697E−02	1.5404E−10	9.3142E−09
	Rank	4	3	1	2
F3	Best	4.0303E+02	4.0000E+02	4.0000E+02	4.0000E+02
	Avg	4.0953E+02	4.0449E+02	4.0013E+02	4.0000E+02
	Std	1.0640E+01	1.3602E+00	7.2785E−01	1.7662E−12
	Rank	4	3	2	1
F4	Best	5.0505E+02	5.0303E+02	5.0497E+02	5.0298E+02
	Avg	5.1769E+02	5.1138E+02	5.1685E+02	5.1078E+02
	Std	8.5445E+00	5.7685E+00	8.1960E+00	5.6951E+00
	Rank	4	2	3	1
F5	Best	6.0016E+02	6.0004E+02	6.0016E+02	6.0002E+02
	Avg	6.0110E+02	6.0019E+02	6.0103E+02	6.0017E+02
	Std	9.5631E−01	1.0506E−01	8.8274E−01	1.0257E−01
	Rank	4	2	3	1
F6	Best	7.1329E+02	7.1310E+02	7.1286E+02	7.1072E+02
	Avg	7.2679E+02	7.2121E+02	7.2569E+02	7.1951E+02
	Std	6.0918E+00	4.5164E+00	6.0832E+00	4.6670E+00
	Rank	4	2	3	1
F7	Best	8.0972E+02	8.0558E+02	8.0895E+02	8.0398E+02
	Avg	8.1907E+02	8.1154E+02	8.1798E+02	8.1104E+02
	Std	7.7645E+00	4.2171E+00	6.6149E+00	4.4138E+00
	Rank	4	2	3	1
F8	Best	9.0027E+02	9.0000E+02	9.0000E+02	9.0000E+02
	Avg	9.0491E+02	9.0006E+02	9.0440E+02	9.0004E+02
	Std	4.8763E+00	1.0959E−01	4.6523E+00	9.1327E−02
	Rank	4	2	3	1
F9	Best	1.0659E+03	1.1909E+03	1.0219E+03	1.1584E+03
	Avg	1.7605E+03	1.7498E+03	1.6884E+03	1.6804E+03
	Std	2.6692E+02	2.3358E+02	2.5713E+02	2.1666E+02
	Rank	4	3	2	1
F10	Best	1.1028E+03	1.1012E+03	1.1020E+03	1.1000E+03
	Avg	1.1203E+03	1.1051E+03	1.1172E+03	1.1023E+03
	Std	1.6984E+01	1.9034E+00	1.7136E+01	1.5750E+00
	Rank	4	2	3	1
F11	Best	3.0566E+03	1.2142E+03	1.3349E+03	1.2002E+03
	Avg	1.5166E+05	1.4241E+03	1.6757E+03	1.3987E+03
	Std	7.2602E+05	1.5771E+02	2.1927E+02	1.6017E+02
	Rank	4	2	3	1
F12	Best	1.3980E+03	1.3041E+03	1.3405E+03	1.3010E+03
	Avg	1.8712E+03	1.3107E+03	1.6999E+03	1.3070E+03
	Std	2.9972E+02	3.7774E+00	2.4182E+02	4.0517E+00
	Rank	4	2	3	1
F13	Best	1.4192E+03	1.4034E+03	1.4120E+03	1.4015E+03
	Avg	1.4459E+03	1.4144E+03	1.4404E+03	1.4133E+03
	Std	1.2674E+01	8.5524E+00	1.2822E+01	9.1865E+00
	Rank	4	2	3	1
F14	Best	1.5324E+03	1.5006E+03	1.5017E+03	1.5001E+03
	Avg	1.6123E+03	1.5042E+03	1.5653E+03	1.5027E+03
	Std	4.9701E+01	5.4503E+00	4.8430E+01	5.5833E+00
	Rank	4	2	3	1
F15	Best	1.6017E+03	1.6021E+03	1.6012E+03	1.6012E+03
	Avg	1.6619E+03	1.6231E+03	1.6387E+03	1.6106E+03
	Std	6.5476E+01	2.8606E+01	6.3068E+01	2.3817E+01
	Rank	4	2	3	1
F16	Best	1.7240E+03	1.7245E+03	1.7227E+03	1.7212E+03
	Avg	1.7555E+03	1.7439E+03	1.7521E+03	1.7419E+03
	Std	2.1439E+01	1.4873E+01	2.1232E+01	1.5076E+01
	Rank	4	2	3	1
F17	Best	1.9194E+03	1.8025E+03	1.8217E+03	1.8011E+03
	Avg	2.7894E+03	1.8190E+03	1.8908E+03	1.8171E+03
	Std	1.0332E+03	7.8932E+00	7.3157E+01	7.8714E+00
	Rank	4	2	3	1
F18	Best	1.9112E+03	1.9012E+03	1.9057E+03	1.9012E+03
	Avg	1.9351E+03	1.9030E+03	1.9307E+03	1.9028E+03
	Std	1.7186E+01	9.3929E−01	1.8796E+01	9.6429E−01
	Rank	4	2	3	1
F19	Best	2.0217E+03	2.0215E+03	2.0207E+03	2.0209E+03
	Avg	2.0615E+03	2.0441E+03	2.0584E+03	2.0427E+03
	Std	4.3580E+01	1.7493E+01	4.3519E+01	1.7319E+01
	Rank	4	2	3	1
F20	Best	2.2024E+03	2.2000E+03	2.2000E+03	2.2000E+03
	Avg	2.2757E+03	2.2462E+03	2.2732E+03	2.2458E+03
	Std	5.8810E+01	5.7657E+01	6.0122E+01	5.7394E+01
	Rank	4	2	3	1
F21	Best	2.2221E+03	2.2001E+03	2.2200E+03	2.2000E+03
	Avg	2.3009E+03	2.2982E+03	2.3003E+03	2.2979E+03
	Std	1.4971E+01	1.8537E+01	1.5258E+01	1.8491E+01
	Rank	4	2	3	1
F22	Best	2.6048E+03	2.6045E+03	2.6048E+03	2.6044E+03
	Avg	2.6224E+03	2.6179E+03	2.6190E+03	2.6160E+03
	Std	7.6667E+00	7.6482E+00	7.5340E+00	7.5567E+00
	Rank	4	2	3	1
F23	Best	2.5082E+03	2.5002E+03	2.5000E+03	2.5000E+03
	Avg	2.7345E+03	2.7096E+03	2.7319E+03	2.7089E+03
	Std	5.9540E+01	8.3554E+01	6.3397E+01	8.3482E+01
	Rank	4	2	3	1
F24	Best	2.8983E+03	2.8977E+03	2.8980E+03	2.8977E+03
	Avg	2.9372E+03	2.9088E+03	2.9370E+03	2.9088E+03
	Std	2.2128E+01	1.9639E+01	2.2277E+01	1.9632E+01
	Rank	4	2	3	1
F25	Best	2.9007E+03	2.9001E+03	2.8000E+03	2.9000E+03
	Avg	2.9817E+03	2.9031E+03	2.9472E+03	2.9028E+03
	Std	1.2520E+02	1.5661E+01	5.7171E+01	1.5377E+01
	Rank	4	2	3	1
F26	Best	3.0897E+03	3.0890E+03	3.0884E+03	3.0884E+03
	Avg	3.0948E+03	3.0920E+03	3.0932E+03	3.0912E+03
	Std	5.3588E+00	5.3996E+00	4.4834E+00	5.4627E+00
	Rank	4	2	3	1
F27	Best	3.1529E+03	3.1001E+03	3.1000E+03	3.1000E+03
	Avg	3.3255E+03	3.1810E+03	3.2075E+03	3.1355E+03
	Std	1.0843E+02	1.2315E+02	8.1564E+01	6.4819E+01
	Rank	4	2	3	1
F28	Best	3.1438E+03	3.1401E+03	3.1377E+03	3.1362E+03
	Avg	3.1868E+03	3.1676E+03	3.1801E+03	3.1604E+03
	Std	3.2361E+01	2.1425E+01	3.1560E+01	2.0189E+01
	Rank	4	2	3	1
F29	Best	3.7905E+03	3.4075E+03	3.2588E+03	3.2377E+03
	Avg	4.7647E+05	3.1212E+04	3.5650E+03	3.3417E+03
	Std	6.1227E+05	1.4926E+05	6.5659E+02	7.2574E+01
	Rank	4	3	2	1

**Table A6:** Experimental results of ERBMO with different strategies (D = 30).

No.	Index	RBMO	DRBMO	PRBMO	ERBMO
F1	Min	2.7381E+08	3.6999E+06	1.0000E+02	1.0000E+02
	Avg	8.6658E+08	1.9816E+07	1.0001E+02	1.0008E+02
	Std	4.9476E+08	1.2950E+07	6.5667E−02	4.3531E−01
	Rank	4	3	1	2
F2	Best	1.6133E+04	3.8464E+02	3.0000E+02	3.0000E+02
	Avg	2.7655E+04	7.4576E+02	3.0000E+02	3.0000E+02
	Std	5.7764E+03	3.1544E+02	9.6955E−05	6.8726E−06
	Rank	4	3	2	1
F3	Best	5.4586E+02	4.7382E+02	4.0000E+02	4.0000E+02
	Avg	6.2965E+02	5.2062E+02	4.0106E+02	4.0133E+02
	Std	5.5968E+01	1.9350E+01	1.7931E+00	1.9114E+00
	Rank	4	3	1	2
F4	Best	5.9414E+02	5.7438E+02	5.4179E+02	5.2885E+02
	Avg	6.2756E+02	6.0193E+02	5.8640E+02	5.5562E+02
	Std	2.0109E+01	1.3533E+01	2.0996E+01	1.2777E+01
	Rank	4	3	2	1
F5	Best	6.0885E+02	6.0286E+02	6.0792E+02	6.0239E+02
	Avg	6.1664E+02	6.0522E+02	6.1472E+02	6.0441E+02
	Std	4.8735E+00	1.5166E+00	4.6151E+00	1.3659E+00
	Rank	4	2	3	1
F6	Best	8.5194E+02	7.9908E+02	8.1083E+02	7.5036E+02
	Avg	9.0557E+02	8.2608E+02	8.5769E+02	7.7545E+02
	Std	3.2731E+01	1.7591E+01	3.3525E+01	1.5416E+01
	Rank	4	2	3	1
F7	Best	8.9450E+02	8.6485E+02	8.5373E+02	8.2487E+02
	Avg	9.2970E+02	8.9787E+02	8.8848E+02	8.5097E+02
	Std	2.0750E+01	1.8674E+01	2.1967E+01	1.4867E+01
	Rank	4	3	2	1
F8	Best	1.2349E+03	9.1353E+02	1.1657E+03	9.0836E+02
	Avg	1.9336E+03	9.5399E+02	1.6704E+03	9.3898E+02
	Std	3.5516E+02	3.6803E+01	2.6486E+02	2.6321E+01
	Rank	4	2	3	1
F9	Best	4.2557E+03	4.5456E+03	3.3720E+03	3.5113E+03
	Avg	5.7211E+03	6.2292E+03	4.6662E+03	4.8199E+03
	Std	7.8239E+02	6.3952E+02	6.6079E+02	6.8165E+02
	Rank	3	4	1	2
F10	Best	1.3031E+03	1.1510E+03	1.1627E+03	1.1249E+03
	Avg	1.4244E+03	1.1949E+03	1.2455E+03	1.1372E+03
	Std	8.9461E+01	2.7097E+01	5.6090E+01	1.0465E+01
	Rank	4	2	3	1
F11	Best	2.1413E+06	1.2778E+04	1.7580E+03	1.5608E+03
	Avg	2.8943E+07	2.9143E+05	2.9852E+03	2.5382E+03
	Std	3.4161E+07	3.3738E+05	6.8049E+02	4.8067E+02
	Rank	4	3	2	1
F12	Best	2.8591E+04	3.2870E+03	2.2883E+03	1.9505E+03
	Avg	2.2092E+05	6.4935E+03	7.5322E+03	3.7281E+03
	Std	3.2469E+05	2.0132E+03	8.7551E+03	9.3293E+02
	Rank	4	2	3	1
F13	Best	1.6721E+03	1.4422E+03	1.4853E+03	1.4330E+03
	Avg	3.2024E+03	1.4691E+03	1.6090E+03	1.4499E+03
	Std	1.8831E+03	1.3487E+01	1.0815E+02	1.1778E+01
	Rank	4	2	3	1
F14	Best	2.0534E+04	1.6497E+03	1.6378E+03	1.5347E+03
	Avg	5.8458E+04	1.7752E+03	2.8040E+03	1.6192E+03
	Std	2.6361E+04	6.5385E+01	1.7747E+03	4.7566E+01
	Rank	4	2	3	1
F15	Best	2.0231E+03	1.9417E+03	1.7387E+03	1.6147E+03
	Avg	2.8015E+03	2.5645E+03	2.4187E+03	2.1752E+03
	Std	3.8976E+02	3.2524E+02	3.4478E+02	3.0958E+02
	Rank	4	3	2	1
F16	Best	1.9069E+03	1.8151E+03	1.8138E+03	1.7818E+03
	Avg	2.1690E+03	2.0582E+03	2.1102E+03	2.0087E+03
	Std	1.7306E+02	1.4995E+02	1.8338E+02	1.4009E+02
	Rank	4	2	3	1
F17	Best	4.2829E+04	1.8584E+03	2.0207E+03	1.8249E+03
	Avg	1.3088E+05	1.9583E+03	9.2005E+03	1.8878E+03
	Std	5.9776E+04	5.5559E+01	9.6577E+03	3.5274E+01
	Rank	4	2	3	1
F18	Best	2.8416E+03	1.9460E+03	2.1873E+03	1.9321E+03
	Avg	3.3074E+04	1.9771E+03	7.3161E+03	1.9642E+03
	Std	4.0581E+04	2.2063E+01	7.2640E+03	2.0407E+01
	Rank	4	2	3	1
F19	Best	2.1259E+03	2.1814E+03	2.0771E+03	2.1543E+03
	Avg	2.4398E+03	2.4028E+03	2.3900E+03	2.3544E+03
	Std	1.5885E+02	1.2640E+02	1.5131E+02	1.2178E+02
	Rank	4	3	2	1
F20	Best	2.3831E+03	2.3666E+03	2.3501E+03	2.3312E+03
	Avg	2.4276E+03	2.3900E+03	2.3862E+03	2.3497E+03
	Std	1.9662E+01	1.6139E+01	1.8554E+01	1.5804E+01
	Rank	4	3	2	1
F21	Best	2.4273E+03	2.3218E+03	2.3000E+03	2.3000E+03
	Avg	3.6277E+03	2.5279E+03	3.0892E+03	2.4347E+03
	Std	1.8795E+03	9.5503E+02	1.6171E+03	7.3108E+02
	Rank	4	2	3	1
F22	Best	2.7329E+03	2.7104E+03	2.7023E+03	2.6730E+03
	Avg	2.7840E+03	2.7514E+03	2.7404E+03	2.7050E+03
	Std	2.4955E+01	2.1167E+01	2.2654E+01	1.8020E+01
	Rank	4	3	2	1
F23	Best	2.9068E+03	2.8862E+03	2.8717E+03	2.8422E+03
	Avg	2.9497E+03	2.9179E+03	2.9042E+03	2.8723E+03
	Std	2.3477E+01	2.2337E+01	1.8877E+01	1.6534E+01
	Rank	4	3	2	1
F24	Best	2.9403E+03	2.8926E+03	2.8757E+03	2.8754E+03
	Avg	2.9921E+03	2.9070E+03	2.8847E+03	2.8802E+03
	Std	3.9076E+01	1.2482E+01	1.7108E+01	2.4133E+00
	Rank	4	3	2	1
F25	Best	3.7132E+03	3.1102E+03	2.9000E+03	2.9000E+03
	Avg	5.1770E+03	4.3159E+03	4.6323E+03	3.9343E+03
	Std	3.8724E+02	5.0296E+02	4.0693E+02	5.0521E+02
	Rank	4	2	3	1
F26	Best	3.2169E+03	3.2066E+03	3.1645E+03	3.1194E+03
	Avg	3.2462E+03	3.2199E+03	3.1855E+03	3.1724E+03
	Std	1.9480E+01	9.7124E+00	1.3506E+01	1.2133E+01
	Rank	4	3	2	1
F27	Best	3.3389E+03	3.2248E+03	3.1000E+03	3.1000E+03
	Avg	3.4808E+03	3.2661E+03	3.1791E+03	3.1507E+03
	Std	1.8192E+02	2.2817E+01	7.4568E+01	6.0538E+01
	Rank	4	3	2	1
F28	Best	3.5916E+03	3.5233E+03	3.4056E+03	3.2093E+03
	Avg	3.9322E+03	3.7814E+03	3.8097E+03	3.6819E+03
	Std	2.1825E+02	1.6129E+02	2.0788E+02	1.7866E+02
	Rank	4	2	3	1
F29	Best	6.5026E+04	7.1501E+03	3.3978E+03	3.3716E+03
	Avg	5.2510E+05	1.2005E+04	4.2787E+03	3.8919E+03
	Std	3.5853E+05	7.0467E+03	1.2792E+03	5.4254E+02
	Rank	4	3	2	1

**Table A7:** Experimental results of ERBMO with different strategies (D = 50).

No.	Index	RBMO	DRBMO	PRBMO	ERBMO
F1	Min	6.0331E+09	3.3773E+08	1.0000E+02	1.0000E+02
	Avg	9.4115E+09	7.1869E+08	1.0000E+02	1.0061E+02
	Std	1.9344E+09	2.1537E+08	2.4574E−03	2.6101E+00
	Rank	4	3	1	2
F2	Best	5.8864E+04	4.2541E+03	3.0001E+02	3.0000E+02
	Avg	8.8086E+04	1.0378E+04	3.2824E+02	3.0192E+02
	Std	1.5224E+04	3.7998E+03	7.3493E+01	9.9523E+00
	Rank	4	3	2	1
F3	Best	9.9116E+02	6.4432E+02	4.0000E+02	4.0000E+02
	Avg	1.3552E+03	7.2370E+02	4.1023E+02	4.0574E+02
	Std	2.2177E+02	4.0796E+01	1.4169E+01	7.5705E+00
	Rank	4	3	2	1
F4	Best	7.5406E+02	6.9771E+02	6.1541E+02	5.5771E+02
	Avg	8.2023E+02	7.3761E+02	6.9806E+02	6.0361E+02
	Std	3.8181E+01	2.0022E+01	4.3788E+01	1.8110E+01
	Rank	4	3	2	1
F5	Best	6.1958E+02	6.0771E+02	6.1483E+02	6.0607E+02
	Avg	6.3039E+02	6.1221E+02	6.2463E+02	6.0972E+02
	Std	7.6101E+00	2.9763E+00	6.2291E+00	2.2628E+00
	Rank	4	2	3	1
F6	Best	1.0884E+03	9.3415E+02	1.0018E+03	7.8687E+02
	Avg	1.3111E+03	9.8446E+02	1.1671E+03	8.3890E+02
	Std	1.0891E+02	2.7279E+01	1.0292E+02	2.4067E+01
	Rank	4	2	3	1
F7	Best	1.0351E+03	9.6968E+02	9.1243E+02	8.5970E+02
	Avg	1.1228E+03	1.0314E+03	9.9458E+02	8.9724E+02
	Std	3.5855E+01	2.5817E+01	4.0128E+01	2.0849E+01
	Rank	4	3	2	1
F8	Best	3.7765E+03	1.2941E+03	2.3347E+03	1.1532E+03
	Avg	6.5377E+03	1.7660E+03	4.3391E+03	1.4005E+03
	Std	2.0190E+03	3.9625E+02	1.3097E+03	2.2722E+02
	Rank	4	2	3	1
F9	Best	9.9778E+03	8.4887E+03	5.9182E+03	5.4831E+03
	Avg	1.1309E+04	1.1217E+04	7.9591E+03	7.7795E+03
	Std	9.8513E+02	8.1240E+02	1.1166E+03	9.3272E+02
	Rank	4	3	2	1
F10	Best	1.7881E+03	1.2715E+03	1.2274E+03	1.1527E+03
	Avg	2.1319E+03	1.3562E+03	1.3300E+03	1.2128E+03
	Std	2.0001E+02	5.2641E+01	6.7027E+01	4.1216E+01
	Rank	4	3	2	1
F11	Best	1.4041E+08	6.8945E+06	2.7940E+03	4.1327E+03
	Avg	4.8286E+08	2.1737E+07	4.7765E+04	1.5975E+04
	Std	2.8754E+08	1.0257E+07	5.1218E+04	1.1160E+04
	Rank	4	3	2	1
F12	Best	3.6295E+05	4.1590E+04	2.8223E+03	3.0936E+03
	Avg	1.6642E+06	7.9114E+04	7.3454E+03	5.3383E+03
	Std	1.0710E+06	3.2822E+04	5.8072E+03	1.2717E+03
	Rank	4	3	2	1
F13	Best	1.7389E+04	1.5735E+03	1.6015E+03	1.4856E+03
	Avg	8.9412E+04	1.6390E+03	6.3425E+03	1.5356E+03
	Std	5.1102E+04	3.3141E+01	1.1487E+04	3.0610E+01
	Rank	4	2	3	1
F14	Best	3.7181E+04	2.8876E+03	2.0592E+03	1.6909E+03
	Avg	1.1255E+05	5.4574E+03	6.5099E+03	1.9934E+03
	Std	4.9934E+04	2.4046E+03	7.5176E+03	3.7555E+02
	Rank	4	2	3	1
F15	Best	2.9759E+03	2.7105E+03	2.1970E+03	1.9951E+03
	Avg	3.6347E+03	3.4912E+03	2.9689E+03	2.8382E+03
	Std	4.1664E+02	3.9127E+02	3.9824E+02	3.2203E+02
	Rank	4	3	2	1
F16	Best	2.4935E+03	2.2980E+03	2.4352E+03	2.1565E+03
	Avg	3.3451E+03	2.9045E+03	3.2025E+03	2.7520E+03
	Std	3.5810E+02	2.9639E+02	3.5740E+02	2.9992E+02
	Rank	4	2	3	1
F17	Best	2.8243E+05	2.3109E+03	2.1151E+03	1.9177E+03
	Avg	1.2801E+06	2.5223E+03	1.6403E+04	2.0624E+03
	Std	1.2393E+06	1.4627E+02	1.5293E+04	8.4575E+01
	Rank	4	2	3	1
F18	Best	4.7014E+04	2.1690E+03	2.4878E+03	2.0790E+03
	Avg	7.2180E+05	4.0444E+03	1.0994E+04	3.2304E+03
	Std	9.7924E+05	4.3045E+03	9.7495E+03	1.9629E+03
	Rank	4	2	3	1
F19	Best	2.5566E+03	2.6992E+03	2.4553E+03	2.6044E+03
	Avg	3.3377E+03	3.2167E+03	3.1775E+03	3.0635E+03
	Std	3.8043E+02	2.2747E+02	3.8183E+02	2.1865E+02
	Rank	4	3	2	1
F20	Best	2.5496E+03	2.5092E+03	2.4190E+03	2.3694E+03
	Avg	2.5968E+03	2.5454E+03	2.4660E+03	2.4097E+03
	Std	3.3005E+01	2.7836E+01	3.2274E+01	2.4251E+01
	Rank	4	3	2	1
F21	Best	1.0211E+04	1.0652E+04	7.3574E+03	6.8831E+03
	Avg	1.2394E+04	1.2771E+04	9.2005E+03	9.2190E+03
	Std	1.0235E+03	8.4468E+02	1.0346E+03	9.0983E+02
	Rank	3	4	1	2
F22	Best	2.9971E+03	2.9314E+03	2.8699E+03	2.7956E+03
	Avg	3.0880E+03	2.9789E+03	2.9529E+03	2.8426E+03
	Std	4.1045E+01	2.8823E+01	4.2158E+01	3.0601E+01
	Rank	4	3	2	1
F23	Best	3.1492E+03	3.0968E+03	3.0148E+03	2.9756E+03
	Avg	3.2546E+03	3.1550E+03	3.1214E+03	3.0240E+03
	Std	5.7388E+01	2.9093E+01	5.5210E+01	2.6631E+01
	Rank	4	3	2	1
F24	Best	3.3920E+03	3.1121E+03	2.9312E+03	2.9312E+03
	Avg	3.8937E+03	3.1558E+03	2.9508E+03	2.9591E+03
	Std	2.8965E+02	2.9339E+01	1.9186E+01	2.5971E+01
	Rank	4	3	1	2
F25	Best	6.5093E+03	5.6291E+03	5.4409E+03	4.5226E+03
	Avg	7.5054E+03	6.1546E+03	6.1714E+03	4.9901E+03
	Std	6.8584E+02	2.5020E+02	6.3425E+02	2.5223E+02
	Rank	4	2	3	1
F26	Best	3.3941E+03	3.3253E+03	3.1407E+03	3.1248E+03
	Avg	3.5723E+03	3.4673E+03	3.2466E+03	3.2457E+03
	Std	1.0277E+02	9.9072E+01	1.1530E+02	7.7985E+01
	Rank	4	3	2	1
F27	Best	3.8491E+03	3.3782E+03	3.2709E+03	3.2393E+03
	Avg	5.4813E+03	3.4497E+03	3.2990E+03	3.2776E+03
	Std	1.2630E+03	6.7619E+01	5.3081E+00	2.5224E+01
	Rank	4	3	2	1
F28	Best	4.3527E+03	3.9920E+03	3.8470E+03	3.4621E+03
	Avg	4.9837E+03	4.5116E+03	4.3621E+03	3.9035E+03
	Std	3.6870E+02	2.6028E+02	3.2239E+02	2.5257E+02
	Rank	4	3	2	1
F29	Best	1.4680E+07	4.1167E+06	3.5242E+03	3.4058E+03
	Avg	3.7992E+07	6.7810E+06	7.0962E+03	4.7423E+03
	Std	1.7559E+07	1.7294E+06	3.3999E+03	1.3096E+03
	Rank	4	3	2	1

**Table A8:** Experimental results of ERBMO with different strategies (D = 100).

No.	Index	RBMO	DRBMO	PRBMO	ERBMO
F1	Min	6.4004E+10	1.1980E+10	1.0000E+02	1.0000E+02
	Avg	7.7533E+10	1.6102E+10	1.0006E+02	1.0015E+02
	Std	1.1181E+10	3.0678E+09	3.4260E−01	7.6732E−01
	Rank	4	3	1	2
F2	Best	2.2049E+05	9.4000E+04	6.3357E+02	4.6408E+02
	Avg	2.7225E+05	1.2597E+05	1.6236E+03	1.1585E+03
	Std	2.5224E+04	1.5426E+04	9.7497E+02	8.4385E+02
	Rank	4	3	2	1
F3	Best	5.8067E+03	1.6677E+03	4.0004E+02	4.0000E+02
	Avg	9.0835E+03	2.2173E+03	4.5896E+02	4.2287E+02
	Std	2.0074E+03	3.0261E+02	3.7001E+01	3.1363E+01
	Rank	4	3	2	1
F4	Best	1.3255E+03	1.0874E+03	9.0196E+02	6.6317E+02
	Avg	1.4353E+03	1.1833E+03	1.0216E+03	7.4907E+02
	Std	5.5471E+01	5.0708E+01	6.4094E+01	4.1101E+01
	Rank	4	3	2	1
F5	Best	6.4482E+02	6.2221E+02	6.2996E+02	6.1613E+02
	Avg	6.5708E+02	6.2805E+02	6.4170E+02	6.2022E+02
	Std	7.4802E+00	3.1013E+00	5.7337E+00	2.3057E+00
	Rank	4	2	3	1
F6	Best	2.5144E+03	1.4108E+03	1.9872E+03	9.5490E+02
	Avg	2.9404E+03	1.5382E+03	2.4639E+03	1.0910E+03
	Std	2.6348E+02	5.9182E+01	2.6229E+02	5.8696E+01
	Rank	4	2	3	1
F7	Best	1.6161E+03	1.3901E+03	1.2189E+03	9.9800E+02
	Avg	1.7576E+03	1.4921E+03	1.3435E+03	1.0656E+03
	Std	7.2348E+01	4.8800E+01	7.0144E+01	4.0584E+01
	Rank	4	3	2	1
F8	Best	1.8162E+04	5.6359E+03	8.2256E+03	2.8378E+03
	Avg	3.1021E+04	7.6800E+03	1.4600E+04	3.8257E+03
	Std	6.4737E+03	1.3079E+03	2.7271E+03	6.9294E+02
	Rank	4	2	3	1
F9	Best	2.3380E+04	2.2395E+04	1.1988E+04	1.2538E+04
	Avg	2.5570E+04	2.5615E+04	1.5624E+04	1.5526E+04
	Std	1.3376E+03	1.2410E+03	1.4279E+03	1.3225E+03
	Rank	3	4	2	1
F10	Best	2.2241E+04	2.7723E+03	1.8273E+03	1.7001E+03
	Avg	3.3267E+04	3.6379E+03	2.3284E+03	1.9874E+03
	Std	8.0844E+03	4.3123E+02	5.5257E+02	1.4249E+02
	Rank	4	3	2	1
F11	Best	6.4645E+09	6.1661E+08	1.1950E+04	6.7055E+03
	Avg	8.8429E+09	9.3083E+08	1.0638E+05	3.7651E+04
	Std	1.7410E+09	2.2250E+08	1.2780E+05	3.7894E+04
	Rank	4	3	2	1
F12	Best	4.6843E+07	1.4017E+06	6.1158E+03	5.3695E+03
	Avg	2.1165E+08	4.4236E+06	1.3597E+04	7.7289E+03
	Std	1.4035E+08	1.4865E+06	6.8629E+03	2.0210E+03
	Rank	4	3	2	1
F13	Best	3.6439E+05	2.3829E+03	3.4672E+03	1.7030E+03
	Avg	2.0695E+06	2.8524E+03	4.6736E+04	1.9115E+03
	Std	9.5729E+05	3.6145E+02	3.6214E+04	1.2877E+02
	Rank	4	2	3	1
F14	Best	5.3399E+05	7.7685E+04	2.0382E+03	1.7502E+03
	Avg	1.0002E+07	1.4196E+05	8.7070E+03	4.6081E+03
	Std	2.0410E+07	5.7193E+04	9.2621E+03	4.2235E+03
	Rank	4	3	2	1
F15	Best	5.9416E+03	5.6567E+03	3.9399E+03	3.5791E+03
	Avg	7.6010E+03	6.9640E+03	5.2599E+03	4.8452E+03
	Std	8.4885E+02	6.3314E+02	6.7337E+02	6.3060E+02
	Rank	4	3	2	1
F16	Best	4.5737E+03	4.4959E+03	4.2514E+03	3.9440E+03
	Avg	6.0029E+03	5.1982E+03	5.5174E+03	4.6359E+03
	Std	6.1800E+02	4.6472E+02	6.2685E+02	4.2266E+02
	Rank	4	2	3	1
F17	Best	1.6493E+06	1.3300E+04	1.2225E+04	2.1322E+03
	Avg	4.0486E+06	2.2821E+04	5.0673E+04	4.1820E+03
	Std	1.4349E+06	5.7478E+03	2.8364E+04	1.4149E+03
	Rank	4	2	3	1
F18	Best	4.6066E+06	3.4270E+05	2.6514E+03	2.5730E+03
	Avg	2.7036E+07	8.2545E+05	7.8382E+03	6.7707E+03
	Std	1.3367E+07	5.1750E+05	5.6383E+03	3.9798E+03
	Rank	4	3	2	1
F19	Best	4.8187E+03	4.8372E+03	4.3413E+03	3.8702E+03
	Avg	5.8825E+03	5.4780E+03	5.2733E+03	4.8614E+03
	Std	5.1798E+02	3.9441E+02	5.0945E+02	4.0840E+02
	Rank	4	3	2	1
F20	Best	3.2207E+03	2.9083E+03	2.7709E+03	2.4894E+03
	Avg	3.3150E+03	3.0226E+03	2.9023E+03	2.5887E+03
	Std	5.7105E+01	5.4054E+01	7.1008E+01	4.4524E+01
	Rank	4	3	2	1
F21	Best	2.5472E+04	2.5705E+04	1.6063E+04	1.5187E+04
	Avg	2.7532E+04	2.7960E+04	1.7943E+04	1.7757E+04
	Std	1.3447E+03	1.0384E+03	1.2775E+03	1.3806E+03
	Rank	3	4	2	1
F22	Best	3.7979E+03	3.5175E+03	3.4127E+03	3.1471E+03
	Avg	3.9378E+03	3.6305E+03	3.5497E+03	3.2389E+03
	Std	8.7157E+01	6.1277E+01	8.5468E+01	5.7527E+01
	Rank	4	3	2	1
F23	Best	4.4315E+03	4.0482E+03	3.9662E+03	3.6283E+03
	Avg	4.6327E+03	4.2661E+03	4.2036E+03	3.8502E+03
	Std	1.2860E+02	1.0030E+02	1.2706E+02	9.6198E+01
	Rank	4	3	2	1
F24	Best	7.1903E+03	4.2770E+03	3.1062E+03	3.1056E+03
	Avg	1.0068E+04	4.6653E+03	3.2434E+03	3.2137E+03
	Std	1.3363E+03	2.3893E+02	6.5652E+01	5.8221E+01
	Rank	4	3	2	1
F25	Best	1.5960E+04	1.2438E+04	1.2237E+04	8.3505E+03
	Avg	1.9351E+04	1.3899E+04	1.5214E+04	9.7972E+03
	Std	1.5640E+03	5.7689E+02	1.5245E+03	5.9085E+02
	Rank	4	2	3	1
F26	Best	3.8458E+03	3.6632E+03	3.2000E+03	3.2000E+03
	Avg	4.0876E+03	3.7997E+03	3.5117E+03	3.5255E+03
	Std	1.2576E+02	7.1043E+01	2.3827E+02	1.1163E+02
	Rank	4	3	1	2
F27	Best	9.3188E+03	4.0891E+03	3.2618E+03	3.2344E+03
	Avg	1.4892E+04	4.8212E+03	3.2962E+03	3.2951E+03
	Std	3.6113E+03	4.5144E+02	1.2897E+01	1.7467E+01
	Rank	4	3	2	1
F28	Best	7.8634E+03	6.7059E+03	6.1036E+03	4.8663E+03
	Avg	9.2607E+03	7.5913E+03	6.9328E+03	5.8272E+03
	Std	7.2004E+02	4.9423E+02	5.2131E+02	4.6625E+02
	Rank	4	3	2	1
F29	Best	5.6716E+07	7.5145E+06	4.2342E+03	3.9250E+03
	Avg	1.7473E+08	1.7235E+07	9.2773E+03	5.5812E+03
	Std	1.0212E+08	7.7226E+06	4.4851E+03	1.3954E+03
	Rank	4	3	2	1

**Table A9:** Experimental results of ERBMO and competing algorithms (D = 10).

No.	Index	ERBMO	AE	LSHADE-SPACMA	SAO	ACGRIME	QIO	EPSCA	CFOA	EPSCA	CFOA	CFOA
F1	Min	1.0000E+02	8.7626E+03	9.9849E+03	9.6795E+06	1.8422E+03	1.0166E+05	1.4641E+02	1.6826E+07	2.7342E+02	8.0087E+06	6.8101E+05
	Avg	1.0004E+02	3.4021E+04	3.3073E+04	3.1200E+07	5.0537E+04	5.9250E+05	2.6645E+03	1.5933E+08	2.6356E+03	1.5354E+08	2.9322E+06
	Std	2.4024E−01	1.8702E+04	1.4866E+04	1.3280E+07	1.0192E+05	5.4654E+05	2.9650E+03	1.5054E+08	2.9658E+03	1.7581E+08	2.2236E+06
	Rank	1	5	4	9	6	7	3	11	2	10	8
F2	Min	3.0000E+02	6.1794E+02	3.0000E+02	6.1555E+02	3.4388E+02	6.5273E+02	3.1987E+02	5.9274E+02	8.2691E+02	7.0110E+02	8.2010E+02
	Avg	3.0000E+02	1.1874E+03	3.0104E+02	1.3116E+03	1.1183E+03	1.3868E+03	1.1140E+03	2.2425E+03	1.6012E+03	4.3447E+03	1.8638E+03
	Std	9.3142E−09	3.3857E+02	3.2456E+00	5.2871E+02	5.4262E+02	7.0350E+02	1.1726E+03	1.6145E+03	5.3491E+02	2.2826E+03	7.8426E+02
	Rank	1	5	2	6	4	7	3	10	8	11	9
F3	Min	4.0000E+02	4.0577E+02	4.0326E+02	4.0675E+02	4.0264E+02	4.0078E+02	4.0588E+02	4.0807E+02	4.0002E+02	4.0692E+02	4.0398E+02
	Avg	4.0000E+02	4.0631E+02	4.0417E+02	4.0861E+02	4.0715E+02	4.1054E+02	4.0901E+02	4.2362E+02	4.0567E+02	4.2631E+02	4.0690E+02
	Std	1.7662E−12	2.7777E−01	4.5597E−01	5.6142E−01	1.9867E+00	1.6186E+01	1.0263E+01	1.5239E+01	1.8633E+00	2.1077E+01	8.9873E−01
	Rank	1	4	2	7	6	9	8	10	3	11	5
F4	Min	5.0298E+02	5.1036E+02	5.2017E+02	5.1622E+02	5.0199E+02	5.1071E+02	5.0001E+02	5.0941E+02	5.0707E+02	5.1804E+02	5.1004E+02
	Avg	5.1078E+02	5.2910E+02	5.2756E+02	5.3877E+02	5.1556E+02	5.2844E+02	5.0970E+02	5.2298E+02	5.2321E+02	5.3464E+02	5.2311E+02
	Std	5.6951E+00	6.4744E+00	4.4897E+00	8.3720E+00	6.5061E+00	8.5895E+00	7.2428E+00	8.5082E+00	7.8352E+00	1.0360E+01	7.0930E+00
	Rank	2	9	7	11	3	8	1	4	6	10	5
F5	Min	6.0002E+02	6.0013E+02	6.0046E+02	6.0274E+02	6.0004E+02	6.0068E+02	6.0000E+02	6.0587E+02	6.0002E+02	6.0639E+02	6.0070E+02
	Avg	6.0017E+02	6.0023E+02	6.0107E+02	6.0397E+02	6.0051E+02	6.0168E+02	6.0001E+02	6.1542E+02	6.0011E+02	6.1371E+02	6.0176E+02
	Std	1.0257E−01	6.2402E−02	2.7348E−01	8.0155E−01	5.0704E−01	6.8327E−01	1.9164E−02	4.9757E+00	1.5621E−01	5.8246E+00	8.1023E−01
	Rank	3	4	6	9	5	7	1	11	2	10	8
F6	Min	7.1072E+02	7.3282E+02	7.3000E+02	7.4410E+02	7.1762E+02	7.3438E+02	7.1455E+02	7.2014E+02	7.2074E+02	7.2200E+02	7.2592E+02
	Avg	7.1951E+02	7.4136E+02	7.4336E+02	7.5677E+02	7.2629E+02	7.4684E+02	7.1961E+02	7.3484E+02	7.4311E+02	7.4416E+02	7.3887E+02
	Std	4.6670E+00	4.6647E+00	6.9261E+00	7.4088E+00	6.7336E+00	6.1477E+00	3.7103E+00	8.9816E+00	9.1558E+00	1.2796E+01	6.4404E+00
	Rank	1	6	8	11	3	10	2	4	7	9	5
F7	Min	8.0398E+02	8.1638E+02	8.1149E+02	8.1902E+02	8.0697E+02	8.1031E+02	8.0298E+02	8.0924E+02	8.0733E+02	8.1105E+02	8.1176E+02
	Avg	8.1104E+02	8.2786E+02	8.2885E+02	8.3091E+02	8.1651E+02	8.2149E+02	8.1019E+02	8.1831E+02	8.2567E+02	8.2810E+02	8.2153E+02
	Std	4.4138E+00	5.6623E+00	6.5405E+00	7.8644E+00	6.4412E+00	7.2380E+00	4.8627E+00	5.2511E+00	8.8941E+00	7.5282E+00	4.9614E+00
	Rank	2	8	10	11	3	5	1	4	7	9	6
F8	Min	9.0000E+02	9.0001E+02	9.0006E+02	9.0248E+02	9.0003E+02	9.0015E+02	9.0000E+02	9.0768E+02	9.0000E+02	9.1249E+02	9.0034E+02
	Avg	9.0004E+02	9.0005E+02	9.0053E+02	9.0570E+02	9.0369E+02	9.0076E+02	9.0024E+02	9.5988E+02	9.0004E+02	1.0155E+03	9.0255E+02
	Std	9.1327E−02	2.7097E−02	3.5611E−01	2.8828E+00	5.8632E+00	4.4637E−01	6.2761E−01	3.2584E+01	1.0963E−01	7.3932E+01	3.0046E+00
	Rank	2	3	5	9	8	6	4	10	1	11	7
F9	Min	1.1584E+03	1.9643E+03	1.6722E+03	1.4834E+03	1.0565E+03	1.8615E+03	1.0517E+03	1.3750E+03	1.6520E+03	1.3799E+03	1.7835E+03
	Avg	1.6804E+03	2.5550E+03	2.1581E+03	2.1611E+03	1.5137E+03	2.5106E+03	1.6433E+03	1.9486E+03	2.0742E+03	2.0117E+03	2.2085E+03
	Std	2.1666E+02	2.0079E+02	2.1576E+02	3.6027E+02	2.2576E+02	2.4094E+02	2.8742E+02	2.6743E+02	2.3988E+02	2.9047E+02	1.9841E+02
	Rank	3	11	7	8	1	10	2	4	6	5	9
F10	Min	1.1000E+03	1.1086E+03	1.1058E+03	1.1197E+03	1.1050E+03	1.1069E+03	1.1021E+03	1.1131E+03	1.1073E+03	1.1128E+03	1.1094E+03
	Avg	1.1023E+03	1.1128E+03	1.1116E+03	1.1321E+03	1.1204E+03	1.1142E+03	1.1133E+03	1.1785E+03	1.1137E+03	1.1897E+03	1.1192E+03
	Std	1.5750E+00	2.1400E+00	3.1231E+00	6.9992E+00	1.0561E+01	3.4890E+00	4.0840E+01	7.4731E+01	4.4698E+00	4.5982E+01	7.3135E+00
	Rank	1	3	2	9	8	6	4	10	5	11	7
F11	Min	1.2002E+03	4.2460E+04	3.0674E+03	1.2892E+05	1.8607E+04	2.6696E+04	2.8828E+03	8.9757E+03	8.4867E+03	7.5710E+03	1.0665E+05
	Avg	1.3987E+03	1.1048E+05	7.3378E+03	1.5774E+06	1.2975E+06	4.0815E+05	2.6234E+04	9.1623E+05	1.2733E+05	4.9252E+06	5.2996E+05
	Std	1.6017E+02	5.0410E+04	2.6726E+03	1.8860E+06	1.6991E+06	7.0829E+05	2.0969E+04	9.0534E+05	1.6888E+05	4.6075E+06	3.2333E+05
	Rank	1	4	2	10	9	6	3	8	5	11	7
F12	Min	1.3010E+03	1.7230E+03	1.3274E+03	2.8930E+03	1.5361E+03	1.4212E+03	1.3157E+03	1.7980E+03	1.4170E+03	1.9795E+03	1.9112E+03
	Avg	1.3070E+03	3.1968E+03	1.3639E+03	1.5743E+04	9.9025E+03	1.5837E+03	1.2068E+04	7.7430E+03	3.6232E+03	7.7778E+03	3.9235E+03
	Std	4.0517E+00	9.2401E+02	1.8358E+01	1.1539E+04	8.9077E+03	1.4613E+02	1.0116E+04	5.7220E+03	1.4070E+03	6.3959E+03	1.5274E+03
	Rank	1	4	2	11	9	3	10	7	5	8	6
F13	Min	1.4015E+03	1.4526E+03	1.4237E+03	1.4826E+03	1.4249E+03	1.4242E+03	1.4126E+03	1.4656E+03	1.4452E+03	1.4505E+03	1.4394E+03
	Avg	1.4133E+03	1.4911E+03	1.4304E+03	2.1885E+03	1.7619E+03	1.4308E+03	3.5884E+03	1.5289E+03	1.4681E+03	1.4854E+03	1.4814E+03
	Std	9.1865E+00	1.7371E+01	3.5543E+00	1.0068E+03	6.1621E+02	2.8454E+00	3.8576E+03	4.1378E+01	1.4277E+01	2.9436E+01	1.5280E+01
	Rank	1	7	2	10	9	3	11	8	4	6	5
F14	Min	1.5001E+03	1.5703E+03	1.5057E+03	1.7288E+03	1.5101E+03	1.5091E+03	1.5229E+03	1.6158E+03	1.5789E+03	1.5841E+03	1.5966E+03
	Avg	1.5027E+03	1.7683E+03	1.5099E+03	2.4891E+03	1.6644E+03	1.5194E+03	5.7867E+03	2.2352E+03	1.7732E+03	1.7702E+03	1.7654E+03
	Std	5.5833E+00	1.3618E+02	2.8875E+00	9.4365E+02	1.6689E+02	6.6691E+00	6.5779E+03	4.3312E+02	1.7020E+02	2.4225E+02	1.4698E+02
	Rank	1	6	2	10	4	3	11	9	8	7	5
F15	Min	1.6012E+03	1.6396E+03	1.6287E+03	1.6133E+03	1.6020E+03	1.6092E+03	1.6014E+03	1.6113E+03	1.6063E+03	1.6052E+03	1.6114E+03
	Avg	1.6106E+03	1.7118E+03	1.6598E+03	1.6768E+03	1.6519E+03	1.6847E+03	1.6654E+03	1.7289E+03	1.6726E+03	1.7772E+03	1.6467E+03
	Std	2.3817E+01	4.5827E+01	1.8899E+01	5.7653E+01	7.2970E+01	7.5834E+01	8.0531E+01	1.0419E+02	6.6855E+01	1.0634E+02	2.9222E+01
	Rank	1	9	4	7	3	8	5	10	6	11	2
F16	Min	1.7212E+03	1.7588E+03	1.7421E+03	1.7482E+03	1.7078E+03	1.7339E+03	1.7037E+03	1.7355E+03	1.7402E+03	1.7315E+03	1.7307E+03
	Avg	1.7419E+03	1.7873E+03	1.7616E+03	1.7684E+03	1.7313E+03	1.7617E+03	1.7439E+03	1.7616E+03	1.7607E+03	1.7557E+03	1.7614E+03
	Std	1.5076E+01	1.3806E+01	1.0023E+01	1.5261E+01	1.2574E+01	1.5928E+01	3.7500E+01	2.0662E+01	1.3132E+01	1.5808E+01	1.4986E+01
	Rank	2	11	7	10	1	9	3	8	5	4	6
F17	Min	1.8011E+03	2.6144E+03	1.8466E+03	6.5864E+03	1.9105E+03	1.8677E+03	1.9491E+03	2.4556E+03	2.2688E+03	2.0135E+03	2.8614E+03
	Avg	1.8171E+03	6.3476E+03	1.8809E+03	3.8657E+04	1.8594E+04	2.1602E+03	2.1340E+04	1.4286E+04	8.7029E+03	1.9578E+04	9.8111E+03
	Std	7.8714E+00	2.3316E+03	3.6423E+01	1.8677E+04	1.5521E+04	4.1721E+02	1.6781E+04	1.1927E+04	6.4955E+03	1.3699E+04	6.2814E+03
	Rank	1	4	2	11	8	3	10	7	5	9	6
F18	Min	1.9012E+03	1.9428E+03	1.9051E+03	1.9503E+03	1.9073E+03	1.9058E+03	1.9056E+03	1.9267E+03	1.9228E+03	1.9170E+03	1.9493E+03
	Avg	1.9028E+03	2.0887E+03	1.9078E+03	8.4059E+03	2.4790E+03	1.9091E+03	6.7428E+03	2.5352E+03	2.3559E+03	2.0247E+03	2.0832E+03
	Std	9.6429E−01	1.1742E+02	1.2892E+00	6.2283E+03	8.2008E+02	1.9800E+00	6.5177E+03	6.9147E+02	1.5190E+03	8.4778E+01	1.0993E+02
	Rank	1	6	2	11	8	3	10	9	7	4	5
F19	Min	2.0209E+03	2.0415E+03	2.0343E+03	2.0311E+03	2.0186E+03	2.0396E+03	2.0000E+03	2.0268E+03	2.0280E+03	2.0310E+03	2.0279E+03
	Avg	2.0427E+03	2.0790E+03	2.0510E+03	2.0671E+03	2.0247E+03	2.0694E+03	2.0583E+03	2.0740E+03	2.0562E+03	2.0817E+03	2.0569E+03
	Std	1.7319E+01	1.3076E+01	9.4392E+00	1.7398E+01	5.2007E+00	1.5124E+01	5.5260E+01	3.3914E+01	2.0683E+01	2.8728E+01	1.5020E+01
	Rank	2	10	3	7	1	8	6	9	4	11	5
F20	Min	2.2000E+03	2.2175E+03	2.2023E+03	2.2029E+03	2.2020E+03	2.2010E+03	2.2026E+03	2.2038E+03	2.2014E+03	2.2049E+03	2.2023E+03
	Avg	2.2458E+03	2.2991E+03	2.2382E+03	2.2697E+03	2.2421E+03	2.2097E+03	2.2803E+03	2.2307E+03	2.2730E+03	2.2136E+03	2.2195E+03
	Std	5.7394E+01	4.6206E+01	4.4731E+01	6.5891E+01	4.7620E+01	2.2804E+01	5.0668E+01	3.2587E+01	6.1275E+01	7.3464E+00	3.5468E+01
	Rank	7	11	5	8	6	1	10	4	9	2	3
F21	Min	2.2000E+03	2.3024E+03	2.3043E+03	2.2325E+03	2.2206E+03	2.2376E+03	2.2116E+03	2.2400E+03	2.2114E+03	2.2691E+03	2.2140E+03
	Avg	2.2979E+03	2.3048E+03	2.3061E+03	2.3041E+03	2.2905E+03	2.3058E+03	2.2958E+03	2.3181E+03	2.2972E+03	2.3225E+03	2.2886E+03
	Std	1.8491E+01	1.1752E+00	9.6956E−01	2.4091E+01	2.8245E+01	1.2993E+01	2.1484E+01	1.9882E+01	2.1253E+01	2.0735E+01	3.4296E+01
	Rank	5	7	9	6	2	8	3	10	4	11	1
F22	Min	2.6044E+03	2.6136E+03	2.6154E+03	2.6228E+03	2.6083E+03	2.6104E+03	2.6063E+03	2.6154E+03	2.6116E+03	2.6130E+03	2.6097E+03
	Avg	2.6160E+03	2.6265E+03	2.6255E+03	2.6383E+03	2.6171E+03	2.6235E+03	2.6163E+03	2.6311E+03	2.6244E+03	2.6330E+03	2.6215E+03
	Std	7.5567E+00	6.3421E+00	5.2719E+00	6.3690E+00	6.2089E+00	8.2103E+00	7.0100E+00	9.4801E+00	8.8440E+00	1.2392E+01	7.2683E+00
	Rank	1	8	7	11	3	5	2	9	6	10	4
F23	Min	2.5000E+03	2.6734E+03	2.5575E+03	2.5073E+03	2.5001E+03	2.5037E+03	2.5006E+03	2.5248E+03	2.5025E+03	2.5290E+03	2.5193E+03
	Avg	2.7089E+03	2.7493E+03	2.7326E+03	2.7307E+03	2.6877E+03	2.6731E+03	2.7362E+03	2.6738E+03	2.7357E+03	2.6199E+03	2.6153E+03
	Std	8.3482E+01	1.6311E+01	5.5786E+01	8.9468E+01	1.0520E+02	1.1540E+02	4.5155E+01	8.9026E+01	6.2530E+01	8.1470E+01	7.9714E+01
	Rank	6	11	8	7	5	3	10	4	9	2	1
F24	Min	2.8977E+03	2.8994E+03	2.8986E+03	2.9083E+03	2.8980E+03	2.8995E+03	2.8985E+03	2.9064E+03	2.8977E+03	2.9097E+03	2.8995E+03
	Avg	2.9088E+03	2.9447E+03	2.9323E+03	2.9432E+03	2.9282E+03	2.9262E+03	2.9285E+03	2.9473E+03	2.9234E+03	2.9526E+03	2.9195E+03
	Std	1.9632E+01	8.6282E+00	2.0075E+01	1.3716E+01	2.2284E+01	2.2696E+01	2.3724E+01	1.5333E+01	2.3382E+01	1.6514E+01	1.9585E+01
	Rank	1	9	7	8	5	4	6	10	3	11	2
F25	Min	2.9000E+03	2.9004E+03	2.9004E+03	2.8881E+03	2.9002E+03	2.9037E+03	2.9000E+03	2.9385E+03	2.8003E+03	2.9156E+03	2.9071E+03
	Avg	2.9028E+03	2.9014E+03	2.9007E+03	2.9521E+03	2.9399E+03	2.9172E+03	2.9821E+03	3.0373E+03	2.9253E+03	3.0268E+03	2.9286E+03
	Std	1.5377E+01	9.0652E−01	2.4032E−01	2.2809E+01	3.1014E+01	1.3031E+01	2.1078E+02	5.9112E+01	5.0556E+01	5.3244E+01	1.8238E+01
	Rank	3	2	1	8	7	4	9	11	5	10	6
F26	Min	3.0884E+03	3.0915E+03	3.0898E+03	3.0927E+03	3.0897E+03	3.0931E+03	3.0890E+03	3.0913E+03	3.0893E+03	3.0930E+03	3.0901E+03
	Avg	3.0912E+03	3.0948E+03	3.0909E+03	3.0957E+03	3.0920E+03	3.0998E+03	3.0918E+03	3.0986E+03	3.0919E+03	3.0986E+03	3.0935E+03
	Std	5.4627E+00	1.2491E+00	7.8545E−01	1.7629E+00	1.8831E+00	3.0894E+00	2.7522E+00	4.1809E+00	2.3787E+00	3.7061E+00	2.2323E+00
	Rank	2	7	1	8	5	11	3	10	4	9	6
F27	Min	3.1000E+03	3.1141E+03	3.1020E+03	3.1723E+03	3.1001E+03	3.1093E+03	3.1705E+03	3.1216E+03	3.1115E+03	3.1887E+03	3.1285E+03
	Avg	3.1355E+03	3.2379E+03	3.1425E+03	3.2798E+03	3.2413E+03	3.1902E+03	3.3447E+03	3.2463E+03	3.2769E+03	3.2546E+03	3.1728E+03
	Std	6.4819E+01	1.0768E+02	7.5876E+01	1.0380E+02	1.0376E+02	8.9554E+01	9.2866E+01	6.1892E+01	1.1831E+02	6.2508E+01	1.3378E+01
	Rank	1	5	2	10	6	4	11	7	9	8	3
F28	Min	3.1362E+03	3.1864E+03	3.1593E+03	3.1724E+03	3.1461E+03	3.1777E+03	3.1360E+03	3.1572E+03	3.1661E+03	3.1517E+03	3.1585E+03
	Avg	3.1604E+03	3.2163E+03	3.2159E+03	3.2314E+03	3.1824E+03	3.2204E+03	3.1997E+03	3.2111E+03	3.2099E+03	3.2075E+03	3.2017E+03
	Std	2.0189E+01	1.6112E+01	1.8789E+01	3.9051E+01	2.7484E+01	2.2763E+01	4.8544E+01	2.5878E+01	2.7987E+01	4.0325E+01	1.9602E+01
	Rank	1	9	8	11	2	10	3	7	6	5	4
F29	Min	3.2377E+03	5.3278E+04	3.6320E+03	1.8046E+04	3.9008E+03	1.4733E+04	5.4352E+03	4.7797E+03	6.6980E+03	4.6003E+03	1.1901E+04
	Avg	3.3417E+03	3.8369E+05	6.6092E+04	5.7088E+05	4.0480E+05	2.8430E+05	5.7058E+05	3.5802E+05	5.0761E+05	3.7757E+05	1.3663E+05
	Std	7.2574E+01	3.1207E+05	2.1741E+05	6.7634E+05	4.3521E+05	5.2254E+05	4.3337E+05	4.9146E+05	3.0622E+05	5.0902E+05	1.1289E+05
	Rank	1	7	2	11	8	4	10	5	9	6	3

**Table A10:** Experimental results of ERBMO and competing algorithms (D = 30).

No.	Index	ERBMO	AE	LSHADE-SPACMA	SAO	ACGRIME	QIO	EPSCA	CFOA	EPSCA	CFOA	CFOA
F1	Min	1.0000E+02	1.3641E+07	1.2549E+06	3.0437E+09	1.3449E+08	4.7464E+07	1.3915E+06	6.3210E+09	2.3236E+05	3.3003E+09	1.8358E+08
	Avg	1.0008E+02	2.1465E+07	2.4187E+06	4.2792E+09	4.3181E+08	1.8419E+08	5.1654E+06	1.0753E+10	1.2973E+06	8.9827E+09	4.4113E+08
	Std	4.3531E−01	5.7086E+06	6.7761E+05	7.6752E+08	2.1443E+08	7.8950E+07	4.5185E+06	2.1835E+09	8.7728E+05	2.6231E+09	1.5860E+08
	Rank	1	5	3	9	7	6	4	11	2	10	8
F2	Min	3.0000E+02	4.3734E+04	2.5595E+03	5.4658E+04	2.4045E+04	4.4551E+04	3.1258E+04	3.2880E+04	2.4934E+04	3.8200E+04	3.6009E+04
	Avg	3.0000E+02	6.0787E+04	1.5729E+04	6.4927E+04	3.4548E+04	6.5759E+04	7.5324E+04	4.6434E+04	4.7659E+04	5.8552E+04	5.6417E+04
	Std	6.8726E−06	1.0421E+04	2.6154E+04	5.8617E+03	6.1625E+03	1.2661E+04	2.1161E+04	7.7863E+03	9.6956E+03	1.2003E+04	1.1636E+04
	Rank	1	8	2	9	3	10	11	4	5	7	6
F3	Min	4.0000E+02	5.2246E+02	4.8867E+02	6.6055E+02	5.2710E+02	5.2702E+02	4.9007E+02	1.2041E+03	4.7700E+02	7.7686E+02	5.4313E+02
	Avg	4.0133E+02	5.3360E+02	4.9214E+02	8.2610E+02	5.8607E+02	5.8802E+02	5.2096E+02	2.0742E+03	5.1890E+02	1.1599E+03	5.9956E+02
	Std	1.9114E+00	6.5803E+00	2.4529E+00	7.8458E+01	3.2688E+01	3.5989E+01	1.9944E+01	5.0388E+02	2.4009E+01	3.2834E+02	2.5866E+01
	Rank	1	5	2	9	6	7	4	11	3	10	8
F4	Min	5.2885E+02	6.6413E+02	6.6459E+02	7.2330E+02	5.7203E+02	6.4935E+02	5.2185E+02	6.6545E+02	5.9916E+02	6.5063E+02	6.1688E+02
	Avg	5.5562E+02	6.8507E+02	6.9042E+02	7.5047E+02	6.1346E+02	6.9203E+02	5.4522E+02	7.1110E+02	6.6830E+02	7.1830E+02	6.4985E+02
	Std	1.2777E+01	9.6851E+00	1.0928E+01	1.4522E+01	2.1790E+01	2.3444E+01	1.1546E+01	2.8542E+01	3.3376E+01	3.4260E+01	2.0545E+01
	Rank	2	6	7	11	3	8	1	9	5	10	4
F5	Min	6.0239E+02	6.0206E+02	6.0183E+02	6.2326E+02	6.0353E+02	6.1119E+02	6.0051E+02	6.3563E+02	6.0203E+02	6.3336E+02	6.0641E+02
	Avg	6.0441E+02	6.0310E+02	6.0257E+02	6.2906E+02	6.0877E+02	6.1756E+02	6.0131E+02	6.4614E+02	6.0530E+02	6.4547E+02	6.1127E+02
	Std	1.3659E+00	6.9252E−01	3.6010E−01	3.3637E+00	2.8469E+00	4.4747E+00	5.5821E−01	4.7282E+00	2.4850E+00	6.8662E+00	2.2402E+00
	Rank	4	3	2	9	6	8	1	11	5	10	7
F6	Min	7.5036E+02	8.8210E+02	9.0597E+02	1.0028E+03	8.0651E+02	9.1096E+02	7.6401E+02	9.5148E+02	8.6526E+02	9.3472E+02	8.8282E+02
	Avg	7.7545E+02	9.1994E+02	9.2905E+02	1.0396E+03	8.5239E+02	9.5723E+02	7.8975E+02	1.0171E+03	9.3307E+02	1.0434E+03	9.1928E+02
	Std	1.5416E+01	1.4103E+01	1.1073E+01	2.3158E+01	2.5430E+01	1.9953E+01	1.8543E+01	3.5760E+01	2.6426E+01	5.7607E+01	1.9565E+01
	Rank	1	5	6	10	3	8	2	9	7	11	4
F7	Min	8.2487E+02	9.6529E+02	9.6642E+02	1.0197E+03	8.7029E+02	9.3578E+02	8.3021E+02	9.1487E+02	8.8725E+02	9.4385E+02	8.9651E+02
	Avg	8.5097E+02	9.8695E+02	9.9423E+02	1.0365E+03	8.9917E+02	9.7431E+02	8.4886E+02	9.8025E+02	9.6447E+02	1.0016E+03	9.4143E+02
	Std	1.4867E+01	8.8385E+00	1.0205E+01	1.2319E+01	1.6416E+01	2.4877E+01	1.2963E+01	2.6944E+01	3.1397E+01	2.9829E+01	1.9587E+01
	Rank	2	8	9	11	3	6	1	7	5	10	4
F8	Min	9.0836E+02	9.1032E+02	9.0828E+02	2.1061E+03	1.1255E+03	1.1276E+03	9.0628E+02	3.0914E+03	9.1427E+02	2.4518E+03	1.0615E+03
	Avg	9.3898E+02	9.2673E+02	9.1330E+02	2.8442E+03	1.9059E+03	1.5107E+03	9.4250E+02	4.0821E+03	1.0938E+03	4.3583E+03	1.3337E+03
	Std	2.6321E+01	1.0321E+01	3.6323E+00	4.9130E+02	5.9411E+02	3.7612E+02	2.8864E+01	7.7461E+02	1.2889E+02	8.9237E+02	1.7523E+02
	Rank	3	2	1	9	8	7	4	10	5	11	6
F9	Min	3.5113E+03	7.7933E+03	7.5691E+03	7.6160E+03	3.9934E+03	8.0431E+03	3.2367E+03	4.9824E+03	6.1614E+03	5.1172E+03	6.9196E+03
	Avg	4.8199E+03	8.4282E+03	8.3696E+03	8.4291E+03	5.0527E+03	8.5582E+03	4.6454E+03	6.5559E+03	7.9751E+03	6.0609E+03	7.8270E+03
	Std	6.8165E+02	3.0452E+02	3.3899E+02	3.4244E+02	5.6653E+02	2.6010E+02	5.3147E+02	7.5312E+02	7.4707E+02	5.2211E+02	3.8341E+02
	Rank	2	9	8	10	3	11	1	5	7	4	6
F10	Min	1.1249E+03	1.4121E+03	1.2012E+03	1.6507E+03	1.2344E+03	1.3498E+03	1.1442E+03	1.4945E+03	1.4446E+03	1.6631E+03	1.3824E+03
	Avg	1.1372E+03	1.4903E+03	1.2453E+03	1.9046E+03	1.3641E+03	1.4364E+03	1.2350E+03	2.7056E+03	1.5258E+03	2.6051E+03	1.5747E+03
	Std	1.0465E+01	4.9036E+01	2.2048E+01	1.5085E+02	7.1431E+01	5.5688E+01	4.7289E+01	8.6701E+02	5.9850E+01	5.7256E+02	8.3923E+01
	Rank	1	6	3	9	4	5	2	11	7	10	8
F11	Min	1.5608E+03	1.8991E+06	3.0841E+05	1.5343E+08	4.8347E+06	3.9818E+06	1.6005E+05	1.1979E+08	1.6348E+05	8.0616E+07	3.9584E+06
	Avg	2.5382E+03	3.4631E+06	5.7019E+05	4.1529E+08	2.2296E+07	1.2718E+07	1.6822E+06	5.6387E+08	2.0279E+06	4.2894E+08	1.5204E+07
	Std	4.8067E+02	7.3166E+05	1.4804E+05	1.3002E+08	1.8368E+07	7.5761E+06	1.5979E+06	2.8971E+08	1.4010E+06	3.2555E+08	9.4590E+06
	Rank	1	5	2	9	8	6	3	11	4	10	7
F12	Min	1.9505E+03	3.9817E+04	5.4506E+04	4.1257E+07	1.5205E+04	6.1968E+04	1.5730E+03	1.3983E+06	1.3684E+04	9.3125E+05	1.7260E+05
	Avg	3.7281E+03	5.6006E+04	9.7889E+04	1.2264E+08	8.7467E+04	1.4509E+05	2.0469E+04	5.3866E+07	4.5182E+04	3.1211E+07	5.8321E+05
	Std	9.3293E+02	1.0506E+04	2.8594E+04	5.0355E+07	5.8701E+04	6.6706E+04	2.2711E+04	6.5712E+07	2.7982E+04	5.5791E+07	4.5680E+05
	Rank	1	4	6	11	5	7	2	10	3	9	8
F13	Min	1.4330E+03	5.6331E+03	1.6792E+03	3.0032E+04	5.8621E+03	5.7658E+03	2.8589E+03	3.2084E+03	3.3277E+03	2.2130E+03	5.1521E+03
	Avg	1.4499E+03	2.0205E+04	1.8412E+03	1.6128E+05	9.7674E+04	3.5155E+04	6.5903E+04	5.0531E+04	2.2545E+04	7.3427E+04	2.2429E+04
	Std	1.1778E+01	8.7941E+03	1.1127E+02	1.1436E+05	1.0409E+05	1.9411E+04	8.3860E+04	5.5591E+04	1.6803E+04	6.6578E+04	1.1634E+04
	Rank	1	3	2	11	10	6	8	7	5	9	4
F14	Min	1.5347E+03	1.3238E+04	7.7559E+03	4.3698E+05	5.9460E+03	7.4198E+03	1.5905E+03	1.7199E+04	9.7630E+03	1.2169E+04	1.6868E+04
	Avg	1.6192E+03	1.8814E+04	1.4087E+04	2.5043E+06	1.8714E+04	2.5508E+04	8.5890E+03	2.9073E+05	2.5495E+04	3.1665E+05	1.0508E+05
	Std	4.7566E+01	4.3462E+03	4.4625E+03	2.0087E+06	1.4466E+04	1.0728E+04	1.0765E+04	4.0114E+05	1.2942E+04	9.3085E+05	8.0962E+04
	Rank	1	5	3	11	4	7	2	9	6	10	8
F15	Min	1.6147E+03	2.9066E+03	2.6247E+03	2.9538E+03	1.8899E+03	2.2876E+03	1.7921E+03	2.2168E+03	2.0615E+03	2.2938E+03	2.4269E+03
	Avg	2.1752E+03	3.2710E+03	3.1977E+03	3.5054E+03	2.4699E+03	3.0809E+03	2.2302E+03	2.8143E+03	2.9939E+03	3.1535E+03	2.9028E+03
	Std	3.0958E+02	1.7429E+02	1.9072E+02	2.0213E+02	2.4386E+02	3.4831E+02	3.3466E+02	3.2440E+02	3.3273E+02	3.7406E+02	2.1375E+02
	Rank	1	10	9	11	3	7	2	4	6	8	5
F16	Min	1.7818E+03	2.1089E+03	2.0173E+03	1.9319E+03	1.7751E+03	1.8330E+03	1.7478E+03	1.8697E+03	1.9244E+03	1.9947E+03	1.8453E+03
	Avg	2.0087E+03	2.3354E+03	2.2976E+03	2.2587E+03	1.9621E+03	2.0275E+03	2.0296E+03	2.2730E+03	2.0947E+03	2.3222E+03	2.0131E+03
	Std	1.4009E+02	7.7385E+01	1.3091E+02	1.9275E+02	1.1107E+02	1.1755E+02	2.1783E+02	1.9770E+02	1.1047E+02	1.8322E+02	1.2107E+02
	Rank	2	11	9	7	1	4	5	8	6	10	3
F17	Min	1.8249E+03	1.3064E+05	8.8787E+03	4.3005E+05	5.8233E+04	2.1953E+05	9.1897E+04	1.0060E+05	9.0439E+04	9.2361E+04	1.6583E+05
	Avg	1.8878E+03	3.0806E+05	2.0571E+04	2.3060E+06	5.4857E+05	8.8079E+05	1.4805E+06	7.2876E+05	4.7512E+05	7.8722E+05	6.4520E+05
	Std	3.5274E+01	1.0697E+05	5.5701E+03	1.4587E+06	3.5196E+05	5.4780E+05	1.4522E+06	6.3727E+05	3.5012E+05	6.1225E+05	5.0280E+05
	Rank	1	3	2	11	5	9	10	7	4	8	6
F18	Min	1.9321E+03	1.3676E+04	3.5507E+03	1.8843E+06	3.7821E+03	1.1654E+04	1.9173E+03	1.1807E+04	3.3412E+03	5.9999E+04	9.6290E+03
	Avg	1.9642E+03	2.8839E+04	7.7444E+03	8.5866E+06	6.7758E+04	3.3063E+04	9.5838E+03	1.5210E+06	1.3325E+04	4.1182E+06	2.7313E+05
	Std	2.0407E+01	1.2419E+04	2.0437E+03	4.6899E+06	6.4094E+04	1.7914E+04	1.1145E+04	2.8794E+06	1.0701E+04	4.2039E+06	2.2920E+05
	Rank	1	5	2	11	7	6	3	9	4	10	8
F19	Min	2.1543E+03	2.4857E+03	2.4772E+03	2.3310E+03	2.0863E+03	2.3536E+03	2.0363E+03	2.2305E+03	2.2421E+03	2.2264E+03	2.2387E+03
	Avg	2.3544E+03	2.7345E+03	2.7750E+03	2.5567E+03	2.2938E+03	2.6591E+03	2.3787E+03	2.4648E+03	2.6116E+03	2.5876E+03	2.4340E+03
	Std	1.2178E+02	8.9035E+01	1.2188E+02	1.6518E+02	1.2441E+02	1.5048E+02	1.8710E+02	1.2963E+02	1.8062E+02	1.1666E+02	1.0789E+02
	Rank	2	10	11	6	1	9	3	5	8	7	4
F20	Min	2.3312E+03	2.4584E+03	2.4579E+03	2.4930E+03	2.3597E+03	2.4435E+03	2.3303E+03	2.4146E+03	2.3876E+03	2.4381E+03	2.4104E+03
	Avg	2.3497E+03	2.4798E+03	2.4824E+03	2.5287E+03	2.3911E+03	2.4825E+03	2.3474E+03	2.4884E+03	2.4619E+03	2.5073E+03	2.4356E+03
	Std	1.5804E+01	1.1522E+01	1.2025E+01	1.2781E+01	1.8528E+01	2.2069E+01	1.2869E+01	2.8223E+01	3.2174E+01	2.9620E+01	1.5466E+01
	Rank	2	6	7	11	3	8	1	9	5	10	4
F21	Min	2.3000E+03	2.3222E+03	2.3151E+03	2.6613E+03	2.3704E+03	2.3563E+03	2.3145E+03	3.2506E+03	2.3062E+03	3.0211E+03	2.3534E+03
	Avg	2.4347E+03	2.3276E+03	2.3185E+03	2.8569E+03	2.4331E+03	2.3729E+03	3.9983E+03	3.8448E+03	2.5664E+03	3.6388E+03	2.4134E+03
	Std	7.3108E+02	3.8123E+00	1.4123E+00	1.0238E+02	3.5950E+01	1.2519E+01	1.7631E+03	4.1746E+02	1.3540E+03	3.7450E+02	2.5799E+01
	Rank	6	2	1	8	5	3	11	10	7	9	4
F22	Min	2.6730E+03	2.7479E+03	2.8076E+03	2.8652E+03	2.7151E+03	2.8135E+03	2.6761E+03	2.8053E+03	2.7383E+03	2.8241E+03	2.7642E+03
	Avg	2.7050E+03	2.8279E+03	2.8360E+03	2.9094E+03	2.7523E+03	2.8757E+03	2.6997E+03	2.9270E+03	2.8058E+03	2.9203E+03	2.7938E+03
	Std	1.8020E+01	2.7132E+01	1.2196E+01	1.8040E+01	2.0773E+01	3.2016E+01	1.5041E+01	5.2193E+01	3.6351E+01	4.9784E+01	2.1394E+01
	Rank	2	6	7	9	3	8	1	11	5	10	4
F23	Min	2.8422E+03	2.9057E+03	2.9673E+03	3.0451E+03	2.8957E+03	2.9781E+03	2.8385E+03	3.0162E+03	2.8885E+03	2.9891E+03	2.9074E+03
	Avg	2.8723E+03	2.9894E+03	3.0044E+03	3.0713E+03	2.9329E+03	3.0351E+03	2.8700E+03	3.0809E+03	2.9547E+03	3.0874E+03	2.9569E+03
	Std	1.6534E+01	2.9542E+01	1.1375E+01	1.4991E+01	2.4261E+01	3.3610E+01	1.7263E+01	3.4881E+01	4.7911E+01	5.3514E+01	2.0102E+01
	Rank	2	6	7	9	3	8	1	10	4	11	5
F24	Min	2.8754E+03	2.9004E+03	2.8875E+03	2.9986E+03	2.9433E+03	2.9092E+03	2.8901E+03	3.1774E+03	2.8886E+03	3.1303E+03	2.9281E+03
	Avg	2.8802E+03	2.9250E+03	2.8890E+03	3.0529E+03	2.9968E+03	2.9564E+03	2.9092E+03	3.2927E+03	2.9222E+03	3.2781E+03	2.9729E+03
	Std	2.4133E+00	1.1211E+01	8.2534E−01	3.1976E+01	2.5194E+01	2.2044E+01	1.3159E+01	5.7002E+01	2.3739E+01	8.5517E+01	2.1906E+01
	Rank	1	5	2	9	8	6	3	11	4	10	7
F25	Min	2.9000E+03	4.9235E+03	5.1767E+03	5.7550E+03	3.4099E+03	3.0321E+03	3.8666E+03	3.8389E+03	3.2593E+03	3.8667E+03	4.5698E+03
	Avg	3.9343E+03	5.3682E+03	5.4273E+03	6.2228E+03	4.3781E+03	5.1637E+03	4.1531E+03	6.4405E+03	4.9096E+03	5.6757E+03	5.0793E+03
	Std	5.0521E+02	1.6113E+02	1.0444E+02	2.1366E+02	4.8342E+02	1.0872E+03	2.1965E+02	7.8966E+02	5.9087E+02	8.6922E+02	2.2783E+02
	Rank	1	7	8	10	3	6	2	11	4	9	5
F26	Min	3.1194E+03	3.2322E+03	3.2137E+03	3.2410E+03	3.2205E+03	3.2975E+03	3.2023E+03	3.2739E+03	3.2051E+03	3.2624E+03	3.2394E+03
	Avg	3.1724E+03	3.2492E+03	3.2240E+03	3.2703E+03	3.2352E+03	3.3585E+03	3.2209E+03	3.3573E+03	3.2279E+03	3.3340E+03	3.2733E+03
	Std	1.2133E+01	8.4142E+00	5.0896E+00	1.6980E+01	1.0001E+01	3.6782E+01	1.0812E+01	4.0035E+01	1.2498E+01	3.8521E+01	1.8683E+01
	Rank	1	6	3	7	5	11	2	10	4	9	8
F27	Min	3.1000E+03	3.2761E+03	3.2200E+03	3.4805E+03	3.2931E+03	3.2823E+03	3.2603E+03	3.6433E+03	3.2236E+03	3.5557E+03	3.2648E+03
	Avg	3.1507E+03	3.2987E+03	3.2353E+03	3.5448E+03	3.3578E+03	3.3490E+03	3.2921E+03	3.9314E+03	3.2678E+03	3.8142E+03	3.3469E+03
	Std	6.0538E+01	1.1860E+01	1.1686E+01	4.8712E+01	3.4571E+01	3.9448E+01	2.8746E+01	2.0559E+02	2.2990E+01	2.0102E+02	3.7372E+01
	Rank	1	5	2	9	8	7	4	11	3	10	6
F28	Min	3.2093E+03	3.8819E+03	3.8195E+03	4.0371E+03	3.5468E+03	3.6969E+03	3.3149E+03	3.9742E+03	3.7639E+03	3.6742E+03	3.5898E+03
	Avg	3.6819E+03	4.1404E+03	4.0404E+03	4.4233E+03	3.7734E+03	4.0197E+03	3.7049E+03	4.4172E+03	3.9916E+03	4.1927E+03	3.9098E+03
	Std	1.7866E+02	1.1850E+02	1.2931E+02	2.1413E+02	1.3726E+02	2.0809E+02	2.3157E+02	1.9857E+02	1.4246E+02	2.3473E+02	1.6017E+02
	Rank	1	8	7	11	3	6	2	10	5	9	4
F29	Min	3.3716E+03	1.1347E+05	2.4643E+04	8.0575E+06	6.7421E+04	2.3598E+05	7.1409E+03	1.9710E+06	2.0814E+04	2.2402E+06	1.2635E+05
	Avg	3.8919E+03	2.6078E+05	4.3814E+04	2.4794E+07	1.2587E+06	9.9136E+05	1.6271E+04	7.1492E+06	1.2665E+05	1.7755E+07	2.3463E+06
	Std	5.4254E+02	1.1195E+05	1.2528E+04	1.0477E+07	1.0153E+06	6.5063E+05	5.7157E+03	4.0119E+06	1.1058E+05	1.8171E+07	1.7544E+06
	Rank	1	5	3	11	7	6	2	9	4	10	8

**Table A11:** Experimental results of ERBMO and competing algorithms (D = 50).

No.	Index	ERBMO	AE	LSHADE-SPACMA	SAO	ACGRIME	QIO	EPSCA	CFOA	EPSCA	CFOA	CFOA
F1	Min	1.0000E+02	1.8236E+08	8.6586E+06	1.2680E+10	2.3778E+09	7.5777E+08	1.0603E+08	2.1376E+10	4.1704E+07	1.6136E+10	1.7270E+09
	Avg	1.0061E+02	2.7227E+08	1.2702E+07	1.7084E+10	5.5197E+09	1.3227E+09	3.4295E+08	3.8500E+10	1.2502E+08	2.9296E+10	3.3787E+09
	Std	2.6101E+00	6.1078E+07	1.8111E+06	2.2468E+09	2.0447E+09	3.4529E+08	2.1934E+08	5.3931E+09	6.0019E+07	6.4440E+09	1.0130E+09
	Rank	1	4	2	9	8	6	5	11	3	10	7
F2	Min	3.0000E+02	1.0928E+05	1.8325E+04	1.0385E+05	6.7809E+04	1.1304E+05	7.8595E+04	8.4171E+04	9.2870E+04	9.0907E+04	1.0432E+05
	Avg	3.0192E+02	1.4294E+05	4.6201E+04	1.4107E+05	9.4995E+04	1.5037E+05	1.7696E+05	1.0622E+05	1.1617E+05	1.2611E+05	1.3473E+05
	Std	9.9523E+00	1.6003E+04	2.7359E+04	1.4450E+04	1.5369E+04	1.9297E+04	5.1296E+04	1.4629E+04	1.1267E+04	1.9610E+04	1.7977E+04
	Rank	1	9	2	8	3	10	11	4	5	6	7
F3	Min	4.0000E+02	6.8843E+02	5.5140E+02	1.5125E+03	8.0686E+02	7.8643E+02	6.0322E+02	5.0595E+03	5.8203E+02	2.0765E+03	8.4391E+02
	Avg	4.0574E+02	7.2376E+02	6.1607E+02	2.4138E+03	1.1410E+03	9.2463E+02	6.7570E+02	6.9198E+03	6.8196E+02	4.2923E+03	9.9964E+02
	Std	7.5705E+00	2.0575E+01	2.1510E+01	4.3102E+02	2.5978E+02	7.1598E+01	3.3559E+01	1.0300E+03	5.9834E+01	1.6162E+03	1.1334E+02
	Rank	1	5	2	9	8	6	3	11	4	10	7
F4	Min	5.5771E+02	8.4495E+02	8.2782E+02	9.4505E+02	7.1142E+02	8.0250E+02	5.9788E+02	8.4623E+02	7.1108E+02	8.8382E+02	7.7478E+02
	Avg	6.0361E+02	8.7932E+02	8.6858E+02	9.9748E+02	7.9725E+02	8.7977E+02	6.3386E+02	9.1669E+02	8.6003E+02	9.6717E+02	8.1865E+02
	Std	1.8110E+01	1.3444E+01	1.4100E+01	2.4604E+01	4.8135E+01	3.2667E+01	2.0973E+01	3.3235E+01	5.9483E+01	4.1275E+01	2.9216E+01
	Rank	1	7	6	11	3	8	2	9	5	10	4
F5	Min	6.0607E+02	6.0449E+02	6.0278E+02	6.3144E+02	6.1246E+02	6.2067E+02	6.0295E+02	6.5142E+02	6.0974E+02	6.4828E+02	6.1761E+02
	Avg	6.0972E+02	6.0629E+02	6.0314E+02	6.4408E+02	6.2037E+02	6.3233E+02	6.0503E+02	6.6419E+02	6.1713E+02	6.6142E+02	6.2241E+02
	Std	2.2628E+00	7.9388E−01	2.8713E−01	5.4041E+00	5.3920E+00	5.7245E+00	1.3874E+00	4.7213E+00	3.2061E+00	7.3327E+00	3.0349E+00
	Rank	4	3	1	9	6	8	2	11	5	10	7
F6	Min	7.8687E+02	1.1136E+03	1.0588E+03	1.3328E+03	1.0151E+03	1.1688E+03	8.6765E+02	1.2987E+03	1.0837E+03	1.3070E+03	1.0951E+03
	Avg	8.3890E+02	1.1537E+03	1.1200E+03	1.3990E+03	1.0773E+03	1.2524E+03	9.2777E+02	1.4329E+03	1.1923E+03	1.5399E+03	1.1631E+03
	Std	2.4067E+01	1.8846E+01	2.0559E+01	3.0173E+01	3.9778E+01	3.7338E+01	3.9884E+01	5.1707E+01	4.1754E+01	1.0245E+02	3.5505E+01
	Rank	1	5	4	9	3	8	2	10	7	11	6
F7	Min	8.5970E+02	1.1207E+03	1.1362E+03	1.2540E+03	1.0413E+03	1.1108E+03	8.8683E+02	1.1599E+03	9.9045E+02	1.1559E+03	1.0787E+03
	Avg	8.9724E+02	1.1799E+03	1.1687E+03	1.3065E+03	1.0967E+03	1.1823E+03	9.2346E+02	1.2392E+03	1.1647E+03	1.2646E+03	1.1293E+03
	Std	2.0849E+01	1.9706E+01	1.0645E+01	2.4493E+01	3.5409E+01	4.0366E+01	1.9229E+01	3.4294E+01	6.5248E+01	4.9349E+01	2.7634E+01
	Rank	1	7	6	11	3	8	2	9	5	10	4
F8	Min	1.1532E+03	1.1652E+03	9.2442E+02	7.2093E+03	2.8911E+03	3.2912E+03	1.0998E+03	1.0706E+04	1.7811E+03	1.0192E+04	1.9994E+03
	Avg	1.4005E+03	1.3122E+03	9.3858E+02	1.1264E+04	8.0266E+03	7.3626E+03	1.4455E+03	1.6311E+04	4.0613E+03	1.6320E+04	4.5035E+03
	Std	2.2722E+02	9.7517E+01	7.8686E+00	2.2868E+03	3.1844E+03	2.0235E+03	2.1524E+02	3.8825E+03	1.3272E+03	3.4737E+03	1.5437E+03
	Rank	3	2	1	9	8	7	4	10	5	11	6
F9	Min	5.4831E+03	1.3688E+04	1.3368E+04	1.2275E+04	6.9070E+03	1.3960E+04	5.0110E+03	9.1960E+03	1.2560E+04	9.8892E+03	1.2606E+04
	Avg	7.7795E+03	1.4609E+04	1.4741E+04	1.4345E+04	8.9908E+03	1.4745E+04	7.8802E+03	1.1606E+04	1.4592E+04	1.0980E+04	1.3648E+04
	Std	9.3272E+02	4.7301E+02	4.9679E+02	6.0709E+02	8.5393E+02	3.9849E+02	1.0900E+03	1.1181E+03	7.2185E+02	5.8959E+02	4.9391E+02
	Rank	1	9	10	7	3	11	2	5	8	4	6
F10	Min	1.1527E+03	2.4290E+03	1.3434E+03	4.3660E+03	1.6290E+03	1.7592E+03	1.3358E+03	3.6685E+03	2.6599E+03	4.1721E+03	2.4299E+03
	Avg	1.2128E+03	3.1285E+03	1.4086E+03	6.1266E+03	2.2397E+03	2.1448E+03	1.6567E+03	7.6786E+03	4.0830E+03	7.8565E+03	3.5748E+03
	Std	4.1216E+01	3.6063E+02	2.8705E+01	8.9433E+02	3.9474E+02	1.9388E+02	3.0601E+02	2.7035E+03	7.7664E+02	2.1019E+03	7.5354E+02
	Rank	1	6	2	9	5	4	3	10	8	11	7
F11	Min	4.1327E+03	2.4905E+07	4.3417E+06	1.7574E+09	5.2523E+07	7.1995E+07	3.1817E+06	3.9370E+09	3.8324E+06	1.2361E+09	9.7089E+07
	Avg	1.5975E+04	3.9748E+07	7.2307E+06	4.0642E+09	2.0270E+08	1.5353E+08	1.9194E+07	8.1728E+09	2.0224E+07	4.3781E+09	2.0590E+08
	Std	1.1160E+04	8.3975E+06	2.1540E+06	1.1401E+09	1.0847E+08	5.4730E+07	1.1756E+07	2.3844E+09	1.3925E+07	2.1469E+09	7.8709E+07
	Rank	1	5	2	9	7	6	3	11	4	10	8
F12	Min	3.0936E+03	4.3898E+05	3.2898E+05	4.9875E+08	1.0990E+05	1.1112E+06	4.2596E+03	1.5549E+08	3.3841E+04	1.2178E+08	1.7137E+06
	Avg	5.3383E+03	8.1050E+05	5.7119E+05	9.4631E+08	2.2065E+06	3.7811E+06	1.4201E+04	1.2695E+09	7.6559E+04	8.7903E+08	6.6443E+06
	Std	1.2717E+03	2.2713E+05	1.6559E+05	2.7691E+08	4.7844E+06	1.9995E+06	8.6170E+03	8.6283E+08	3.9887E+04	5.8253E+08	3.0385E+06
	Rank	1	5	4	10	6	7	2	11	3	9	8
F13	Min	1.4856E+03	6.7796E+04	2.9628E+03	5.0741E+05	1.7925E+05	6.9827E+04	2.4015E+04	5.2213E+04	3.5262E+04	7.3886E+04	5.9348E+04
	Avg	1.5356E+03	1.6892E+05	5.8202E+03	1.9762E+06	6.9782E+05	3.6615E+05	3.3279E+05	9.3149E+05	1.6890E+05	1.2316E+06	2.3437E+05
	Std	3.0610E+01	5.5783E+04	1.6298E+03	8.0761E+05	4.3947E+05	3.1268E+05	3.5580E+05	5.8157E+05	9.7399E+04	9.3847E+05	1.3731E+05
	Rank	1	4	2	11	8	7	6	9	3	10	5
F14	Min	1.6909E+03	4.9670E+04	5.9094E+04	3.6354E+07	9.3477E+03	6.1985E+04	1.7479E+03	6.8299E+05	6.5813E+03	1.1386E+06	9.0802E+04
	Avg	1.9934E+03	9.3808E+04	1.1287E+05	8.5701E+07	4.4796E+04	1.2517E+05	8.4787E+03	6.7106E+07	2.3140E+04	2.5612E+07	3.7724E+05
	Std	3.7555E+02	2.9406E+04	3.3123E+04	2.9674E+07	3.3307E+04	4.1307E+04	6.5134E+03	6.5916E+07	1.1175E+04	3.3869E+07	2.7759E+05
	Rank	1	5	6	11	4	7	2	10	3	9	8
F15	Min	1.9951E+03	3.7938E+03	4.2969E+03	4.5739E+03	2.4809E+03	2.8151E+03	1.8559E+03	3.2401E+03	2.8977E+03	3.7065E+03	3.1613E+03
	Avg	2.8382E+03	4.3544E+03	4.7648E+03	5.2951E+03	3.0935E+03	3.6244E+03	3.0155E+03	4.0829E+03	3.9700E+03	4.4020E+03	3.6451E+03
	Std	3.2203E+02	2.5821E+02	2.1861E+02	2.9398E+02	3.6866E+02	4.7788E+02	4.3291E+02	3.7533E+02	5.4737E+02	3.5934E+02	3.0558E+02
	Rank	1	8	10	11	3	4	2	7	6	9	5
F16	Min	2.1565E+03	3.2775E+03	3.4285E+03	3.3220E+03	2.4880E+03	2.4115E+03	2.0801E+03	3.2691E+03	2.5903E+03	2.7426E+03	2.4186E+03
	Avg	2.7520E+03	3.7283E+03	3.7512E+03	4.2021E+03	2.8738E+03	3.2034E+03	2.7036E+03	3.7880E+03	3.5534E+03	3.6387E+03	3.1272E+03
	Std	2.9992E+02	1.9686E+02	1.4787E+02	3.0164E+02	2.2226E+02	3.5736E+02	3.5405E+02	3.3724E+02	4.0156E+02	3.9729E+02	3.0252E+02
	Rank	2	8	9	11	3	5	1	10	6	7	4
F17	Min	1.9177E+03	9.7479E+05	4.2163E+04	2.3653E+06	7.6707E+05	7.2172E+05	2.0671E+05	9.6672E+05	4.0011E+05	1.0758E+06	6.1031E+05
	Avg	2.0624E+03	1.9789E+06	7.4142E+04	1.1661E+07	3.3200E+06	3.0333E+06	3.5099E+06	5.7399E+06	2.4948E+06	5.8972E+06	2.6888E+06
	Std	8.4575E+01	5.3327E+05	1.6304E+04	6.4526E+06	2.4313E+06	1.7728E+06	3.0976E+06	4.3882E+06	1.4558E+06	4.1609E+06	1.5400E+06
	Rank	1	3	2	11	7	6	8	9	4	10	5
F18	Min	2.0790E+03	5.2902E+04	2.7110E+04	2.8178E+07	3.4713E+03	5.5710E+04	2.0252E+03	6.1788E+05	3.3044E+03	3.1299E+06	6.4917E+04
	Avg	3.2304E+03	1.0164E+05	5.6441E+04	7.3144E+07	3.2233E+05	1.1908E+05	1.2873E+04	3.0769E+07	1.6478E+04	3.5702E+07	7.7785E+05
	Std	1.9629E+03	2.1584E+04	1.5530E+04	3.0208E+07	3.3709E+05	4.1635E+04	8.9188E+03	3.8770E+07	8.5618E+03	2.4237E+07	5.3374E+05
	Rank	1	5	4	11	7	6	2	9	3	10	8
F19	Min	2.6044E+03	3.4344E+03	3.4456E+03	3.2202E+03	2.5556E+03	3.0143E+03	2.3438E+03	2.7613E+03	3.1107E+03	2.8067E+03	2.7905E+03
	Avg	3.0635E+03	3.8898E+03	3.9864E+03	3.6955E+03	2.8707E+03	3.7924E+03	2.7739E+03	3.3609E+03	3.6991E+03	3.3072E+03	3.3510E+03
	Std	2.1865E+02	1.5170E+02	1.6242E+02	2.5285E+02	1.8570E+02	3.0056E+02	2.8055E+02	2.5830E+02	2.8009E+02	2.2162E+02	2.3224E+02
	Rank	3	10	11	7	2	9	1	6	8	4	5
F20	Min	2.3694E+03	2.6165E+03	2.6313E+03	2.7062E+03	2.4520E+03	2.6067E+03	2.3893E+03	2.6867E+03	2.5090E+03	2.7013E+03	2.5383E+03
	Avg	2.4097E+03	2.6660E+03	2.6714E+03	2.7780E+03	2.5230E+03	2.6694E+03	2.4210E+03	2.7595E+03	2.6277E+03	2.7777E+03	2.6081E+03
	Std	2.4251E+01	1.8364E+01	1.6453E+01	2.4449E+01	4.1097E+01	3.6782E+01	2.1271E+01	4.2627E+01	6.1915E+01	5.0433E+01	2.8291E+01
	Rank	1	6	8	11	3	7	2	9	5	10	4
F21	Min	6.8831E+03	5.8281E+03	5.9983E+03	4.2239E+03	3.4985E+03	2.6894E+03	7.7050E+03	9.1647E+03	1.3807E+04	7.1087E+03	4.8876E+03
	Avg	9.2190E+03	1.5880E+04	1.5848E+04	8.9031E+03	1.0578E+04	1.0629E+04	9.4253E+03	1.2757E+04	1.5871E+04	1.1955E+04	1.4565E+04
	Std	9.0983E+02	1.9842E+03	2.0404E+03	5.6226E+03	1.4766E+03	5.7369E+03	7.6808E+02	1.2737E+03	8.6224E+02	2.2077E+03	2.3184E+03
	Rank	2	11	9	1	4	5	3	7	10	6	8
F22	Min	2.7956E+03	2.9938E+03	3.0519E+03	3.1860E+03	2.9243E+03	3.1253E+03	2.8078E+03	3.2427E+03	2.9291E+03	3.2391E+03	2.9961E+03
	Avg	2.8426E+03	3.1059E+03	3.0961E+03	3.2669E+03	2.9799E+03	3.2512E+03	2.8577E+03	3.3701E+03	3.0800E+03	3.3488E+03	3.0649E+03
	Std	3.0601E+01	2.8390E+01	1.6622E+01	2.9369E+01	3.1614E+01	6.0502E+01	2.0247E+01	7.5236E+01	5.6817E+01	7.6936E+01	3.3763E+01
	Rank	1	7	6	9	3	8	2	11	5	10	4
F23	Min	2.9756E+03	3.1426E+03	3.2082E+03	3.3530E+03	3.0446E+03	3.3360E+03	2.9925E+03	3.3249E+03	3.0532E+03	3.3275E+03	3.1277E+03
	Avg	3.0240E+03	3.2304E+03	3.2578E+03	3.4060E+03	3.1439E+03	3.4343E+03	3.0206E+03	3.5210E+03	3.2266E+03	3.5365E+03	3.2037E+03
	Std	2.6631E+01	3.6795E+01	1.4930E+01	2.6397E+01	4.5616E+01	5.3958E+01	1.9096E+01	8.5592E+01	6.9686E+01	9.4501E+01	2.9246E+01
	Rank	2	6	7	8	3	9	1	10	5	11	4
F24	Min	2.9312E+03	3.1595E+03	3.0341E+03	4.1211E+03	3.4182E+03	3.2402E+03	3.0990E+03	4.8272E+03	3.0999E+03	4.8465E+03	3.2955E+03
	Avg	2.9591E+03	3.2100E+03	3.0495E+03	4.4592E+03	3.6402E+03	3.3830E+03	3.1635E+03	6.6238E+03	3.1859E+03	6.1537E+03	3.5107E+03
	Std	2.5971E+01	2.3759E+01	5.9714E+00	1.8557E+02	1.6559E+02	9.1183E+01	4.0847E+01	6.3731E+02	5.6779E+01	1.0040E+03	1.4307E+02
	Rank	1	5	2	9	8	6	3	11	4	10	7
F25	Min	4.5226E+03	6.5201E+03	6.9350E+03	8.2376E+03	4.4811E+03	4.3150E+03	4.4772E+03	1.0172E+04	4.4408E+03	7.0541E+03	6.3987E+03
	Avg	4.9901E+03	7.4808E+03	7.2078E+03	9.2391E+03	5.5138E+03	7.3174E+03	5.0619E+03	1.2076E+04	6.9818E+03	9.4360E+03	7.0269E+03
	Std	2.5223E+02	2.4238E+02	1.4525E+02	3.5022E+02	6.4256E+02	1.9064E+03	2.8763E+02	8.1218E+02	9.0721E+02	1.0625E+03	3.3182E+02
	Rank	1	8	6	9	3	7	2	11	4	10	5
F26	Min	3.1248E+03	3.4402E+03	3.2584E+03	3.5521E+03	3.4212E+03	3.8648E+03	3.3103E+03	3.7015E+03	3.3133E+03	3.6698E+03	3.5101E+03
	Avg	3.2457E+03	3.5300E+03	3.3007E+03	3.6680E+03	3.5465E+03	4.0767E+03	3.4290E+03	4.0888E+03	3.4596E+03	3.9199E+03	3.6518E+03
	Std	7.7985E+01	4.4535E+01	3.2904E+01	6.2281E+01	7.5861E+01	1.4808E+02	8.1444E+01	1.6802E+02	1.0015E+02	1.9569E+02	6.9491E+01
	Rank	1	5	2	8	6	10	3	11	4	9	7
F27	Min	3.2393E+03	3.4696E+03	3.2942E+03	4.1266E+03	3.7629E+03	3.5682E+03	3.4476E+03	5.4391E+03	3.3948E+03	4.6914E+03	3.7207E+03
	Avg	3.2776E+03	3.5633E+03	3.3227E+03	4.7011E+03	4.2981E+03	3.8730E+03	3.6060E+03	6.6486E+03	3.5263E+03	5.9314E+03	4.1410E+03
	Std	2.5224E+01	5.1547E+01	1.5981E+01	2.8296E+02	2.9004E+02	1.3656E+02	1.2241E+02	5.1273E+02	6.0625E+01	6.2909E+02	2.2348E+02
	Rank	1	4	2	9	8	6	5	11	3	10	7
F28	Min	3.4621E+03	4.8516E+03	4.6973E+03	5.5879E+03	3.8873E+03	4.3546E+03	3.6585E+03	5.4254E+03	4.1075E+03	5.3291E+03	4.0276E+03
	Avg	3.9035E+03	5.2563E+03	4.9086E+03	6.2528E+03	4.4656E+03	5.1013E+03	3.9983E+03	6.7942E+03	4.7547E+03	6.2869E+03	4.7416E+03
	Std	2.5257E+02	1.6137E+02	1.2593E+02	2.9460E+02	3.0675E+02	3.6064E+02	2.0154E+02	8.1901E+02	4.3329E+02	7.3600E+02	3.1469E+02
	Rank	1	8	6	9	3	7	2	11	5	10	4
F29	Min	3.4058E+03	1.0176E+07	3.0986E+06	1.3373E+08	2.5735E+07	2.2555E+07	9.7596E+05	8.5344E+07	4.4570E+06	8.7991E+07	4.4545E+07
	Avg	4.7423E+03	1.6403E+07	4.3744E+06	2.6909E+08	6.1553E+07	3.7415E+07	1.6991E+06	2.1215E+08	8.6560E+06	1.9241E+08	8.2111E+07
	Std	1.3096E+03	2.4829E+06	7.5768E+05	7.6641E+07	2.7071E+07	9.5130E+06	5.5509E+05	7.4930E+07	2.4807E+06	8.1199E+07	2.1762E+07
	Rank	1	5	3	11	7	6	2	10	4	9	8

**Table A12:** Experimental results of ERBMO and competing algorithms (D = 100).

No.	Index	ERBMO	AE	LSHADE-SPACMA	SAO	ACGRIME	QIO	EPSCA	CFOA	EPSCA	CFOA	CFOA
F1	Min	1.0000E+02	2.3911E+09	8.7455E+07	6.0688E+10	3.1476E+10	1.0502E+10	4.9398E+09	1.2251E+11	3.0634E+09	1.0467E+11	2.0888E+10
	Avg	1.0015E+02	3.6438E+09	1.2124E+08	7.8419E+10	4.7111E+10	1.4546E+10	9.1598E+09	1.4191E+11	5.6622E+09	1.3359E+11	3.1580E+10
	Std	7.6732E−01	4.8619E+08	1.8355E+07	8.6176E+09	8.5150E+09	2.2202E+09	2.1333E+09	8.6902E+09	1.5277E+09	1.7636E+10	5.2299E+09
	Rank	1	3	2	9	8	6	5	11	4	10	7
F2	Min	4.6408E+02	3.0273E+05	1.4269E+05	2.6783E+05	2.3581E+05	3.0977E+05	2.7598E+05	2.1301E+05	2.2005E+05	2.5097E+05	2.8819E+05
	Avg	1.1585E+03	3.3027E+05	3.2620E+05	3.2187E+05	2.5531E+05	3.6368E+05	5.1712E+05	2.7041E+05	2.8546E+05	3.0062E+05	3.6458E+05
	Std	8.4385E+02	1.3988E+04	8.2633E+04	1.8558E+04	1.2889E+04	2.8931E+04	8.6227E+04	2.3057E+04	2.2239E+04	2.7003E+04	3.3404E+04
	Rank	1	8	7	6	2	9	11	3	4	5	10
F3	Min	4.0000E+02	1.2403E+03	7.1199E+02	7.6380E+03	3.2207E+03	2.1421E+03	1.2393E+03	1.8432E+04	1.2707E+03	1.4837E+04	2.4501E+03
	Avg	4.2287E+02	1.3702E+03	7.4742E+02	1.0376E+04	5.4324E+03	2.8423E+03	1.5508E+03	2.5478E+04	1.5085E+03	2.3271E+04	3.5667E+03
	Std	3.1363E+01	5.8931E+01	1.6790E+01	1.2175E+03	1.1120E+03	3.7873E+02	1.6978E+02	3.7718E+03	1.3139E+02	4.2224E+03	6.1670E+02
	Rank	1	3	2	9	8	6	5	11	4	10	7
F4	Min	6.6317E+02	1.3527E+03	1.2776E+03	1.6429E+03	1.2307E+03	1.3866E+03	8.6692E+02	1.5845E+03	1.0943E+03	1.5570E+03	1.2709E+03
	Avg	7.4907E+02	1.4503E+03	1.3168E+03	1.7249E+03	1.3755E+03	1.4934E+03	9.5620E+02	1.6575E+03	1.4125E+03	1.7093E+03	1.3776E+03
	Std	4.1101E+01	3.4117E+01	1.9819E+01	4.0556E+01	8.6661E+01	6.8537E+01	4.7821E+01	4.1056E+01	1.2430E+02	8.9629E+01	5.4374E+01
	Rank	1	7	3	11	4	8	2	9	6	10	5
F5	Min	6.1613E+02	6.1231E+02	6.0368E+02	6.6061E+02	6.3431E+02	6.4499E+02	6.1186E+02	6.7517E+02	6.3084E+02	6.6807E+02	6.3626E+02
	Avg	6.2022E+02	6.1471E+02	6.0438E+02	6.6707E+02	6.4435E+02	6.5498E+02	6.1636E+02	6.8227E+02	6.4345E+02	6.7766E+02	6.4286E+02
	Std	2.3057E+00	1.2739E+00	2.3452E−01	3.8553E+00	6.2253E+00	4.5066E+00	2.3721E+00	3.8539E+00	5.9509E+00	5.2629E+00	3.3247E+00
	Rank	4	2	1	9	7	8	3	11	6	10	5
F6	Min	9.5490E+02	1.6964E+03	1.5770E+03	2.4794E+03	1.8153E+03	2.0080E+03	1.3794E+03	2.6854E+03	1.7996E+03	2.9310E+03	1.9156E+03
	Avg	1.0910E+03	1.7382E+03	1.6293E+03	2.6764E+03	2.1251E+03	2.1260E+03	1.5485E+03	2.9831E+03	2.0560E+03	3.2196E+03	2.1528E+03
	Std	5.8696E+01	2.2758E+01	2.1794E+01	8.7136E+01	1.7317E+02	6.7407E+01	7.7303E+01	1.1507E+02	7.7763E+01	1.6284E+02	1.0659E+02
	Rank	1	4	3	9	6	7	2	10	5	11	8
F7	Min	9.9800E+02	1.7036E+03	1.5839E+03	1.8819E+03	1.5117E+03	1.7011E+03	1.1955E+03	1.9016E+03	1.3685E+03	1.8538E+03	1.5755E+03
	Avg	1.0656E+03	1.7575E+03	1.6221E+03	2.0173E+03	1.6373E+03	1.7931E+03	1.2706E+03	2.0217E+03	1.7072E+03	2.0168E+03	1.6702E+03
	Std	4.0584E+01	2.1257E+01	1.8255E+01	4.1911E+01	6.5220E+01	5.2119E+01	3.8764E+01	5.7425E+01	1.5048E+02	8.7828E+01	5.1616E+01
	Rank	1	7	3	10	4	8	2	11	6	9	5
F8	Min	2.8378E+03	4.4494E+03	1.0560E+03	3.7823E+04	2.1893E+04	2.3893E+04	4.4333E+03	3.7188E+04	1.8729E+04	3.4145E+04	1.6698E+04
	Avg	3.8257E+03	5.5266E+03	1.1222E+03	5.1121E+04	3.5775E+04	3.8936E+04	6.5607E+03	4.7629E+04	3.0394E+04	4.7862E+04	2.7270E+04
	Std	6.9294E+02	7.3635E+02	4.0073E+01	5.0580E+03	5.9411E+03	7.7511E+03	1.1877E+03	8.1231E+03	5.7389E+03	6.8308E+03	6.0175E+03
	Rank	2	3	1	11	7	8	4	9	6	10	5
F9	Min	1.2538E+04	3.0419E+04	3.1131E+04	3.0346E+04	1.9104E+04	3.0408E+04	1.5134E+04	2.4093E+04	2.8918E+04	2.3636E+04	2.8809E+04
	Avg	1.5526E+04	3.1653E+04	3.2064E+04	3.1372E+04	2.0960E+04	3.1656E+04	1.8543E+04	2.6258E+04	3.1309E+04	2.5166E+04	2.9989E+04
	Std	1.3225E+03	5.0112E+02	4.1796E+02	4.9760E+02	1.0709E+03	6.2966E+02	1.3921E+03	1.3518E+03	8.7534E+02	9.1882E+02	6.5361E+02
	Rank	1	9	11	8	3	10	2	5	7	4	6
F10	Min	1.7001E+03	5.2551E+04	4.3400E+03	5.0906E+04	2.3406E+04	5.7158E+04	1.0456E+04	3.9034E+04	5.5690E+04	6.2751E+04	5.3771E+04
	Avg	1.9874E+03	7.4548E+04	5.8008E+03	8.0486E+04	3.7732E+04	8.2637E+04	2.2335E+04	6.4008E+04	7.6940E+04	9.0395E+04	7.3249E+04
	Std	1.4249E+02	9.4467E+03	1.5670E+03	1.2234E+04	8.7712E+03	1.1468E+04	7.7952E+03	1.1168E+04	1.0719E+04	2.1368E+04	1.2024E+04
	Rank	1	7	2	9	4	10	3	5	8	11	6
F11	Min	6.7055E+03	6.0692E+08	6.3539E+07	1.3949E+10	1.3764E+09	1.0350E+09	2.7476E+08	3.3246E+10	2.1187E+08	2.1277E+10	1.6318E+09
	Avg	3.7651E+04	7.7740E+08	1.0210E+08	1.9823E+10	4.3143E+09	1.9506E+09	5.7617E+08	5.0489E+10	4.6274E+08	3.5651E+10	2.6324E+09
	Std	3.7894E+04	7.3625E+07	1.9896E+07	2.9259E+09	1.7583E+09	5.1383E+08	1.8051E+08	8.7552E+09	1.5552E+08	8.3613E+09	6.4965E+08
	Rank	1	5	2	9	8	6	4	11	3	10	7
F12	Min	5.3695E+03	4.0310E+06	9.8269E+05	2.1453E+09	1.1119E+07	1.5788E+07	4.1447E+04	3.9259E+09	4.2401E+04	1.3900E+09	2.1192E+07
	Avg	7.7289E+03	6.1658E+06	1.3388E+06	2.9769E+09	1.0885E+08	2.7336E+07	1.4435E+05	8.1425E+09	9.5632E+04	3.0609E+09	4.2227E+07
	Std	2.0210E+03	1.5380E+06	2.5429E+05	4.1199E+08	1.2074E+08	7.8748E+06	1.2279E+05	2.1103E+09	2.5569E+04	1.0400E+09	1.1683E+07
	Rank	1	5	4	9	8	6	3	11	2	10	7
F13	Min	1.7030E+03	1.3872E+06	6.8978E+04	4.1631E+06	2.2709E+06	1.0853E+06	6.6247E+05	1.1975E+06	1.3042E+06	2.6970E+06	1.4886E+06
	Avg	1.9115E+03	2.9427E+06	1.2551E+05	1.2978E+07	5.9662E+06	3.3571E+06	2.9075E+06	9.7907E+06	3.3275E+06	1.0481E+07	4.7958E+06
	Std	1.2877E+02	7.2146E+05	2.3110E+04	4.7374E+06	2.6014E+06	1.5878E+06	1.5133E+06	3.7745E+06	1.3185E+06	6.5477E+06	1.7105E+06
	Rank	1	4	2	11	8	6	3	9	5	10	7
F14	Min	1.7502E+03	3.4028E+05	2.8511E+05	3.4710E+08	7.1551E+04	7.2798E+05	4.6098E+03	6.8468E+08	1.8741E+04	2.3202E+08	5.4953E+05
	Avg	4.6081E+03	7.4588E+05	5.1086E+05	7.5819E+08	6.6898E+06	2.0921E+06	1.0398E+04	1.7063E+09	4.7657E+04	6.3984E+08	3.8709E+06
	Std	4.2235E+03	2.1366E+05	1.0385E+05	1.9205E+08	2.6457E+07	6.2969E+05	5.3930E+03	6.1793E+08	1.6332E+04	3.4981E+08	2.0790E+06
	Rank	1	5	4	10	8	6	2	11	3	9	7
F15	Min	3.5791E+03	8.4095E+03	8.8620E+03	1.0157E+04	5.0350E+03	6.2430E+03	4.4349E+03	9.3330E+03	5.4542E+03	8.7891E+03	6.4157E+03
	Avg	4.8452E+03	9.5644E+03	9.6179E+03	1.1453E+04	6.3747E+03	7.6007E+03	5.2204E+03	1.0734E+04	7.1909E+03	1.0088E+04	7.5584E+03
	Std	6.3060E+02	4.0867E+02	2.9028E+02	4.1547E+02	6.2952E+02	8.4998E+02	5.6119E+02	6.7744E+02	1.1174E+03	8.0216E+02	6.1389E+02
	Rank	1	7	8	11	3	6	2	10	4	9	5
F16	Min	3.9440E+03	5.3142E+03	6.3172E+03	7.8852E+03	3.5160E+03	4.7991E+03	3.3306E+03	6.8607E+03	4.0466E+03	6.7722E+03	4.8283E+03
	Avg	4.6359E+03	6.7501E+03	6.8528E+03	9.0637E+03	5.0840E+03	6.2540E+03	4.3951E+03	1.3263E+04	5.6436E+03	8.4148E+03	5.5667E+03
	Std	4.2266E+02	3.7151E+02	2.2504E+02	4.0497E+02	5.8503E+02	8.4690E+02	5.3664E+02	6.8242E+03	7.5510E+02	1.3432E+03	3.8936E+02
	Rank	2	7	8	10	3	6	1	11	5	9	4
F17	Min	2.1322E+03	2.2425E+06	2.1830E+05	1.0364E+07	1.6010E+06	2.2379E+06	1.1980E+06	2.3932E+06	1.5863E+06	3.3058E+06	3.1638E+06
	Avg	4.1820E+03	3.4351E+06	2.9837E+05	2.1624E+07	6.1244E+06	4.1342E+06	6.6348E+06	9.6484E+06	4.0786E+06	1.5381E+07	6.4965E+06
	Std	1.4149E+03	4.2681E+05	4.5623E+04	4.9905E+06	2.5606E+06	1.2364E+06	5.2728E+06	5.4984E+06	1.5605E+06	6.8002E+06	2.6789E+06
	Rank	1	3	2	11	6	5	8	9	4	10	7
F18	Min	2.5730E+03	1.3948E+06	5.2712E+05	5.1915E+08	7.8054E+05	1.6931E+06	2.9973E+03	7.6875E+08	8.8741E+04	1.7645E+08	3.7636E+06
	Avg	6.7707E+03	1.9965E+06	7.2985E+05	7.2337E+08	9.0274E+06	3.6529E+06	7.4964E+03	1.7302E+09	3.1477E+05	7.2326E+08	8.6202E+06
	Std	3.9798E+03	3.0779E+05	1.1209E+05	1.4187E+08	1.5783E+07	1.2356E+06	3.8398E+03	7.4318E+08	1.7473E+05	4.1106E+08	3.5374E+06
	Rank	1	5	4	10	8	6	2	11	3	9	7
F19	Min	3.8702E+03	6.5924E+03	6.8374E+03	6.2686E+03	4.2247E+03	6.2682E+03	2.9747E+03	5.0242E+03	5.4118E+03	5.1595E+03	5.4032E+03
	Avg	4.8614E+03	7.1607E+03	7.4560E+03	7.2279E+03	5.0679E+03	7.2603E+03	4.5179E+03	5.9202E+03	7.0700E+03	5.9992E+03	6.4086E+03
	Std	4.0840E+02	2.1249E+02	2.1432E+02	2.9938E+02	4.8671E+02	3.9275E+02	5.0190E+02	3.8750E+02	4.7700E+02	3.8439E+02	4.9377E+02
	Rank	2	8	11	9	3	10	1	4	7	5	6
F20	Min	2.4894E+03	3.1709E+03	3.1095E+03	3.4564E+03	2.9358E+03	3.2647E+03	2.6617E+03	3.5779E+03	3.0425E+03	3.4882E+03	3.0426E+03
	Avg	2.5887E+03	3.2276E+03	3.1451E+03	3.5235E+03	3.0733E+03	3.3936E+03	2.7775E+03	3.7263E+03	3.2870E+03	3.6483E+03	3.1472E+03
	Std	4.4524E+01	2.6184E+01	1.8198E+01	4.1158E+01	6.6858E+01	6.0700E+01	5.3462E+01	1.0382E+02	1.0616E+02	8.8378E+01	4.5695E+01
	Rank	1	6	4	9	3	8	2	11	7	10	5
F21	Min	1.5187E+04	3.2825E+04	3.3344E+04	1.0709E+04	7.6792E+03	3.1311E+04	1.9002E+04	2.5546E+04	3.1531E+04	2.6152E+04	2.9960E+04
	Avg	1.7757E+04	3.3967E+04	3.4284E+04	3.1120E+04	2.3444E+04	3.4005E+04	2.1211E+04	2.8555E+04	3.3495E+04	2.7764E+04	3.2198E+04
	Std	1.3806E+03	5.0751E+02	5.2948E+02	6.7872E+03	3.2115E+03	1.0238E+03	9.2178E+02	1.9925E+03	7.1353E+02	7.9724E+02	9.9034E+02
	Rank	1	9	11	6	3	10	2	5	8	4	7
F22	Min	3.1471E+03	3.6800E+03	3.6405E+03	3.9775E+03	3.3607E+03	4.2180E+03	3.1361E+03	4.3409E+03	3.5372E+03	4.3105E+03	3.6621E+03
	Avg	3.2389E+03	3.7537E+03	3.6859E+03	4.1316E+03	3.5360E+03	4.4538E+03	3.2685E+03	4.5391E+03	3.7276E+03	4.6370E+03	3.7618E+03
	Std	5.7527E+01	3.6219E+01	2.0736E+01	5.0525E+01	7.2694E+01	1.1268E+02	5.3618E+01	1.1186E+02	1.1626E+02	1.2816E+02	5.2952E+01
	Rank	1	6	4	8	3	9	2	10	5	11	7
F23	Min	3.6283E+03	4.1171E+03	4.0318E+03	4.6488E+03	4.0017E+03	4.9881E+03	3.6922E+03	5.3198E+03	4.0647E+03	5.0984E+03	4.1877E+03
	Avg	3.8502E+03	4.1792E+03	4.1077E+03	4.7804E+03	4.1327E+03	5.4103E+03	3.7991E+03	5.7721E+03	4.3078E+03	5.7392E+03	4.3233E+03
	Std	9.6198E+01	3.1842E+01	2.6113E+01	6.4043E+01	8.0294E+01	2.1648E+02	5.6084E+01	2.1718E+02	1.2324E+02	2.6453E+02	6.2001E+01
	Rank	2	5	3	8	4	9	1	11	6	10	7
F24	Min	3.1056E+03	4.0264E+03	3.4864E+03	7.8337E+03	5.5937E+03	4.4938E+03	4.1948E+03	1.1468E+04	3.9807E+03	1.0442E+04	5.3623E+03
	Avg	3.2137E+03	4.2088E+03	3.5359E+03	9.4348E+03	6.6505E+03	5.0254E+03	4.4459E+03	1.3228E+04	4.2218E+03	1.3124E+04	5.8947E+03
	Std	5.8221E+01	1.0854E+02	2.1125E+01	6.6578E+02	6.2672E+02	2.4358E+02	1.5482E+02	9.2884E+02	1.5509E+02	1.3659E+03	3.5744E+02
	Rank	1	3	2	9	8	6	5	11	4	10	7
F25	Min	8.3505E+03	1.4092E+04	1.3577E+04	1.9881E+04	1.4033E+04	1.4063E+04	9.5010E+03	2.6309E+04	1.3457E+04	2.1162E+04	1.5103E+04
	Avg	9.7972E+03	1.4816E+04	1.3961E+04	2.0947E+04	1.8646E+04	2.0223E+04	1.0738E+04	3.2072E+04	1.5813E+04	2.6010E+04	1.6910E+04
	Std	5.9085E+02	3.4461E+02	2.0927E+02	6.7607E+02	3.0105E+03	1.7812E+03	5.1083E+02	2.2491E+03	1.2971E+03	2.9748E+03	1.3079E+03
	Rank	1	4	3	9	7	8	2	11	5	10	6
F26	Min	3.2000E+03	3.6199E+03	3.3783E+03	4.2860E+03	3.6027E+03	4.4790E+03	3.4930E+03	4.6917E+03	3.5152E+03	4.7203E+03	4.0329E+03
	Avg	3.5255E+03	3.7227E+03	3.4185E+03	4.5518E+03	3.9114E+03	4.9672E+03	3.6128E+03	5.5050E+03	3.7853E+03	5.3412E+03	4.2085E+03
	Std	1.1163E+02	6.2166E+01	1.6875E+01	1.4835E+02	1.3603E+02	3.0775E+02	7.8399E+01	2.9465E+02	1.2279E+02	3.4983E+02	1.0153E+02
	Rank	2	4	1	8	6	9	3	11	5	10	7
F27	Min	3.2344E+03	4.5412E+03	3.5172E+03	1.0628E+04	7.2589E+03	5.3633E+03	4.8277E+03	1.6301E+04	4.2645E+03	1.2267E+04	6.6247E+03
	Avg	3.2951E+03	4.8676E+03	3.5636E+03	1.2247E+04	8.7724E+03	5.8548E+03	6.1929E+03	1.8490E+04	4.9666E+03	1.6932E+04	7.8766E+03
	Std	1.7467E+01	2.0410E+02	2.1920E+01	6.8272E+02	9.0443E+02	4.3017E+02	2.0029E+03	1.0456E+03	4.4698E+02	2.0735E+03	7.1597E+02
	Rank	1	3	2	9	8	5	6	11	4	10	7
F28	Min	4.8663E+03	9.0457E+03	8.2191E+03	1.0692E+04	7.1605E+03	8.3679E+03	5.5291E+03	1.2771E+04	7.2867E+03	1.0302E+04	7.6773E+03
	Avg	5.8272E+03	9.7321E+03	8.7596E+03	1.2429E+04	8.4749E+03	9.5723E+03	6.2923E+03	1.5952E+04	8.4619E+03	1.3596E+04	8.5603E+03
	Std	4.6625E+02	2.9632E+02	2.5066E+02	6.7872E+02	6.8799E+02	6.2666E+02	5.2094E+02	2.1277E+03	5.9942E+02	2.0855E+03	4.9847E+02
	Rank	1	8	6	9	4	7	2	11	3	10	5
F29	Min	3.9250E+03	1.7493E+07	1.8040E+06	1.4654E+09	8.2437E+07	3.6324E+07	3.8460E+05	1.7071E+09	4.6388E+06	6.3400E+08	3.3205E+07
	Avg	5.5812E+03	2.8239E+07	3.0420E+06	2.3636E+09	1.6764E+08	6.1361E+07	1.2782E+06	5.8808E+09	1.2073E+07	2.1427E+09	1.1459E+08
	Std	1.3954E+03	4.9238E+06	6.0811E+05	4.5615E+08	7.2228E+07	1.7517E+07	7.1330E+05	2.5769E+09	4.2901E+06	8.0010E+08	4.6165E+07
	Rank	1	5	3	10	8	6	2	11	4	9	7
